# Associations of sarcopenia with the risk of incident respiratory disease and the role of inflammation and metabolism: a prospective cohort study

**DOI:** 10.1016/j.jnha.2026.100919

**Published:** 2026-07-02

**Authors:** Xingqi Cao, Xixuan Cai, Jia Zhang, Jingkai Yan, Yanjie Zhao, Xueqing Jia, Kaili Sun, Zuyun Liu, Liying Chen

**Affiliations:** a Department of General Practice, Sir Run Run Shaw Hospital, Zhejiang University School of Medicine, Hangzhou, 310016 Zhejiang, China; b Second Affiliated Hospital, School of Public Health, Zhejiang Key Laboratory of Intelligent Preventive Medicine, Zhejiang University School of Medicine, Hangzhou, 310058 Zhejiang, China; c Center for Aging and Health Studies, Zhejiang University, Hangzhou, 310058 Zhejiang, China

**Keywords:** Sarcopenia, Respiratory disease, Lung function, Inflammation, Metabolite

## Abstract

**Objectives:**

To explore the complex associations of sarcopenia with lung function, and risks of incident respiratory disease (including its subtypes: chronic obstructive pulmonary disease [COPD], asthma, and interstitial lung disease [ILD]), as well as to explore the potential inflammatory and metabolic pathways.

**Design:**

Prospective cohort study.

**Setting and participants:**

We assembled data from 317,628 adults enrolled in the UK Biobank.

**Measurements:**

Sarcopenia status was defined using the European Working Group on Sarcopenia in Older People 2 criteria. Lung function was assessed via a spirometer. The incident respiratory disease was ascertained through linked hospital data over a median follow-up of 14 years.

**Results:**

Both probable sarcopenia and confirmed/severe sarcopenia were consistently associated with poorer lung function and a higher risk of incident respiratory disease. For instance, compared with their non-sarcopenic counterparts, participants with probable sarcopenia exhibited a significantly higher risk (P < 0.001) of respiratory disease (hazard ratio [HR] = 1.30; 95% confidence interval [CI]: 1.26, 1.36), COPD (HR = 1.37; 95% CI: 1.24, 1.50), asthma (HR = 1.35; 95% CI: 1.23, 1.48), and ILD (HR = 1.74; 95% CI: 1.48, 2.04). Furthermore, inflammatory markers and metabolites partially mediated the associations between probable sarcopenia and incident respiratory disease, with C-reactive protein (5.2 %–12.9 %) and albumin (2.6%–8.0%) showing relatively higher mediation proportions.

**Conclusion:**

Sarcopenia, even at the probable stage, was significantly associated with an increased risk of incident respiratory disease, and inflammatory and metabolic mechanisms may underlie these associations. Our findings highlight the importance of early prevention and management of sarcopenia for preserving respiratory health.

## 1 Introduction

Chronic respiratory diseases, including chronic obstructive pulmonary disease (COPD), asthma, and interstitial lung disease (ILD), remain the leading cause of morbidity and mortality, accounting for about 7.0% of global deaths [1,2]. According to the Global Burden of Disease Study 2019, there were more than 500 million people living with chronic respiratory diseases [1,3]. Thereby, identifying high-risk populations susceptible to respiratory diseases is important for developing preventive strategies.

Sarcopenia, characterized by systemic loss of muscle mass and decline in muscle strength or physical function associated with aging, is a progressive and generalized skeletal muscle disorder [4]. Emerging evidence has recognized sarcopenia as a potent contributor to increased morbidity and mortality [[5], [6], [7]]. Muscle mass and strength, two key components of sarcopenia, have been demonstrated to be positively associated with lung function in previous studies [[8], [9], [10], [11]]. To date, few studies suggest associations of sarcopenia with the risk of incident respiratory disease [[12], [13], [14]], but show disadvantages in small sample size [14] and a maximum follow-up period of 7 years [[12], [13], [14]]. A higher proportion of sarcopenia has also been reported in patients with COPD and asthma, relative to the general population [[15], [16], [17]]. Thus, whether sarcopenia exhibits differential effect on respiratory disease and its major subtypes remains unclear.

Of note, how sarcopenia results in the onset of respiratory disease remains to be another question. Mounting evidence further reinforces that this linkage is rooted in a widespread systemic inflammatory state. Chronic low-grade inflammation facilitates muscle catabolism through nuclear factor kappa-B (NF-κB) activation, impaired autophagy, and anabolic resistance [18,19]. Hence, sarcopenia is frequently accompanied by chronic low-grade inflammation, manifested by elevated levels of inflammatory markers, such as C-reactive protein (CRP), tumor necrosis factor-α (TNF-α), and interleukin-6 [20]. Importantly, such systemic inflammation often accumulates with multimorbidity, and is thought to be an important contributor to respiratory disease [[21], [22], [23]]. These findings prompt us to evaluate the role of inflammation in the associations between sarcopenia and respiratory disease. Additionally, metabolic disorders caused by sarcopenia may also be involved in the pathological processes of respiratory disease [[24], [25], [26]]. As a promising tool to help understand the pathophysiology of disease, plasma metabolomics may provide new insight into potential mechanisms linking sarcopenia and pulmonary diseases.

Therefore, we leverage data from the UK Biobank (UKB) to systematically explore the cross-sectional associations of sarcopenia with lung function, as well as the prospective associations with incident respiratory disease and its subtypes (including COPD, asthma, and ILD). Furthermore, we explored the mediating role of inflammatory markers and metabolites in the associations between sarcopenia and incident respiratory disease. The present study could deepen the understanding of complex associations of sarcopenia with respiratory disease, and provide clues for guiding precise prevention of respiratory health issues.

## 2 Materials and methods

### 2.1 Study participants

This study used data from the UKB, a prospective cohort study with over 500,000 adults at recruitment in 2006–2010 [27]. UKB was approved by the North West Multi-Center Research Ethics Committee [28]. Written informed consent was obtained from all participants. Of 499,036 adults aged 40–69 years at baseline, we further excluded those with prevalent respiratory disease (N = 86,482), cancer (N = 24,595), and cardiovascular disease (CVD; N = 31,515), and with missing data on lung function assessment (N = 26,628), sarcopenia component assessment (N = 208), and covariates (e.g., ethnicity, alcoholic consumption; N = 11,980). Finally, a total of 317,628 adults were included in our study (Supplementary Fig. S1).

### 2.2 Assessment of sarcopenia status

Following the European Working Group on sarcopenia in Older People 2 (EWGSOP2) criteria [6], we assessed sarcopenia using three components, including measured handgrip strength (HGS) and muscle mass index (MMI), and self-reported physical performance.

HGS at baseline was measured by a trained research nurse using a Jamar handheld dynamometer. Weak HGS was defined using the maximum value of both hands, with the cut-point being <27 kg for males and <16 kg for females. Additionally, we considered those unable to perform HGS measurement in either hand because of health reasons (e.g., arthritis, stroke, and so on) as having weak HGS. Participants with weak HGS were identified as having probable sarcopenia.

MMI was calculated as appendicular lean mass (ALM) (kg) divided by the squared standing height (m). We used measured fat-free mass (FFM) that assessed through bioelectrical impedance analysis (BIA; Tanita BC418MA, Tokyo, Japan) to calculate ALM with the following equation: ALM (kg) = (0.958 × [Appendicular FFM) (kg)] − (0.166 × *G*) − 0.038, with *G* taking value 0 if female and 1 if male [7]. Standing height (m) was measured using a Seca 202 device. Males with MMI < 7 kg/m^2^ and females with MMI < 5.5 kg/m^2^ were defined as having low MMI. Participants with weak HGS and low MMI were identified as having confirmed sarcopenia.

UKB did not measure objective physical performance. As a surrogate marker, we considered participants who self-reported walking pace as slow, or being unable to walk as poor physical performance. Participants with weak HGS, low MMI, and poor physical performance were identified as having severe sarcopenia. Due to the small number of confirmed but not severe sarcopenia (N = 358), and severe sarcopenia (N = 69), we combined the populations of probable, confirmed, and severe sarcopenia for primary analysis.

### 2.3 Assessment of outcomes

We obtained incident respiratory disease through linked hospital admissions records using the International Classification of Diseases 9th (ICD-9) (all respiratory disease: 415–416, 466–516, 518–519; COPD: 4912, 496; asthma: 493; and ILD: 5019, 5159), and 10th (ICD-10) edition (all respiratory disease: J08-J98, I26-I27; COPD: J44; asthma: J45-J46; and ILD: J60-J64, J66, J702, J703, J704, J84) codes. We considered the incident respiratory disease recorded up to Oct 31, 2022. We calculated the time-to-event from the recruitment date to the date of the first occurrence of the interest event, death, loss to follow-up, or end of follow-up, whichever came first.

At baseline, lung function, including forced expiratory volume in 1 s (FEV1), forced vital capacity (FVC), and peak expiratory flow (PEF), were measured using a Vitalograph Pneumotrac 6800 spirometer (Maids Moreton, UK). Participants were asked to record two blows within approximately 6 min. If the difference in FEV1 and FVC between the first two blows was ≥5%, a third blow was required. We calculated the average value of all available records for each indicator. Then, raw scores of the averaged FVC, FEV1, and PEF measures were converted to z-scores. To account for the influence of factors unrelated to lung function, we also calculated the FEV1/FVC ratio using the maximal values of FEV1 and FVC. Finally, a comprehensive lung function score was created by averaging these z-scores of FEV1, FVC, and PEF.

### 2.4 Covariates

Covariates included age, sex (female, and male), ethnicity (White, Mixed, South Asian, Black, Chinese, and others), educational attainment (high, intermediate, and low), occupational status (working, retired, and others), Townsend deprivation index (TDI), smoking status (never, previous, and current smoker), alcoholic consumption (never or special occasions only, one to three times per month, one to four times per week, and daily or almost daily), healthy diet (yes, and no), regular exercise (yes, and no), sleep duration (in hours), body mass index (BMI) in kg/m^2^, and waist circumference (WC) in cm.

### 2.5 Inflammatory markers and metabolites

Data of plasma inflammatory markers, and metabolites were obtained from baseline blood tests. Inflammatory markers were measured using the Beckman Coulter LH750 Hematology Analyzer. Our study included CRP, and count or percentage of leukocyte, neutrophil, monocyte, lymphocyte, and platelet. Specific ratios, including systemic immune-inflammation index (SII, neutrophil count × platelet/lymphocyte count), neutrophil-to-lymphocyte ratio (NLR, neutrophil count/lymphocyte count), platelet-to-lymphocyte ratio (PLR, platelet/lymphocyte count), and lymphocyte-to-monocyte ratio (LMR, lymphocyte count/monocyte count), were constructed to reflect the systemic inflammatory status. Metabolites were measured with a high-throughput nuclear magnetic resonance (NMR) metabolomics platform, and a total of 168 metabolites in absolute levels were available. All the inflammatory markers and metabolites were transformed using natural logarithmic transformation (ln[x] for inflammatory markers, and ln[x+1] for metabolites), and then converted into z-score for the following statistical analyses.

### 2.6 Statistical analyses

Basic characteristics of the analytic sample in total and by sarcopenia status were described by median (inter-quartile ranges [IQR]) and number (percentage), and differences were compared using the Mann–Whitney U test and Chi-square test for continuous and categorical variables, respectively.

We used general linear regression models to estimate the associations of probable sarcopenia with lung function. Coefficient (i.e., β) and corresponding 95% confidence intervals (CIs) were documented. The differences in cumulative incidence of respiratory disease among categories by sarcopenia status were compared through Kaplan-Meier curves. Cox proportional hazard regression models were used to estimate the associations of probable sarcopenia with the risk of respiratory disease. The Schoenfeld residuals test was used to verify the proportional hazard assumption, and no significant violation was found. Hazard ratios (HRs) and 95% CIs were documented. In both cross-sectional and prospective analyses, two models were performed. Model 1 was adjusted for age, and sex; and model 2 was further adjusted for ethnicity, educational attainment, occupational status, TDI, smoking status, alcohol consumption, healthy diet, regular exercise, sleep duration, BMI, and WC based on model 1. For comparison, we classified participants into three groups, that is, non-sarcopenia, probable sarcopenia, and confirmed/severe sarcopenia, and then estimated their associations with lung function and risk of respiratory disease. Additionally, we estimated the associations of sarcopenia components (i.e., per standard deviation [SD] increment in HGS and MMI, low MMI, and low physical performance) with lung function and risk of respiratory disease. To test the robustness of the associations of probable sarcopenia with lung function and respiratory disease, we first performed stratification analyses by age (<60 years, and ≥60 years), and sex (female, and male), and quantified multiplicative interactions by adding a product term of probable sarcopenia and stratification variable in the models. Second, (1) to account for the influence of lung function at baseline, we further adjusted for z-scores of FEV1, FVC, and PEF at baseline; (2) to reduce the impact of reverse causality, we excluded those who were lost to follow-up, deceased, or diagnosed with incident respiratory disease, within the first 2 years, and we then repeated the primary analyses for incident respiratory disease.

Next, we performed inflammatory and metabolic mechanism analyses for incident respiratory disease. Age, sex, ethnicity, educational attainment, occupational status, TDI, smoking status, alcohol consumption, healthy diet, regular exercise, sleep duration, BMI, and WC were adjusted in all models. First, the associations of probable sarcopenia with inflammatory markers and metabolites were estimated using the general linear regression model. Then, the associations of inflammatory markers and metabolites with the risk of respiratory disease were estimated using the Cox proportional hazard regression model. False discovery rate (FDR) adjusted P < 0.05 indicated statistically significant. Finally, we performed mediation analyses for those variables significantly and consistently associated with probable sarcopenia and the incidence risk of respiratory disease. The R package “mediation” with 1000 simulations was used, and mediation proportions and corresponding 95% CIs were documented.

We conducted all statistical analyses using SAS version 9.4 (SAS Institute, Cary, NC, USA), and R version 4.3.1 (2023-06-16). A two-tailed P < 0.05 indicated statistical significance.

## 3 Results

### 3.1 Basic characteristics

Among 317,628 participants, the median age was 57.1 (IQR: 49.6, 62.8) years, and the majority were White (94.8%). During a median of 14 years of follow-up, 14.6% (46,199/317,628) of participants developed incident respiratory disease, including 6,272 COPD cases, 7,586 asthma cases, and 1,811 ILD cases. Compared with participants who were non-sarcopenic, those with probable sarcopenia were more likely to be older and female, and to have lower educational attainment, and higher TDI. The detailed baseline characteristics of the study participants are shown in Table 1.

**Table 1:** Baseline characteristics of the study participants in total and by sarcopenia status.

Variables	Total (N = 317,628)	Non-sarcopenia (N = 304,232)	Probable sarcopenia (N = 13,396)	P-value
Age in years	57.1 (49.6, 62.8)	56.9 (49.4, 62.6)	61.4 (55.1, 65.5)	<0.001
Sex				<0.001
Female	174,959 (55.1)	166,602 (54.8)	8,357 (62.4)	
Male	142,669 (44.9)	137,630 (45.2)	5,039 (37.6)	
Ethnicity				<0.001
White	301,010 (94.8)	289,021 (95.0)	11,989 (89.5)	
Mixed	1,880 (0.6)	1,793 (0.6)	87 (0.6)	
South Asian	5,825 (1.8)	4,988 (1.6)	837 (6.2)	
Black	5,026 (1.6)	4,827 (1.6)	199 (1.5)	
Chinese	1,079 (0.3)	997 (0.3)	82 (0.6)	
Others	2,808 (0.9)	2,606 (0.9)	202 (1.5)	
Educational attainment				<0.001
High	110,528 (34.8)	107,068 (35.2)	3,460 (25.8)	
Intermediate	105,968 (33.4)	101,777 (33.5)	4,191 (31.3)	
Low	101,132 (31.8)	95,387 (31.4)	5,745 (42.9)	
Occupational status				<0.001
Working	198,875 (62.6)	193,331 (63.5)	5,544 (41.4)	
Retired	94,310 (29.7)	88,210 (29.0)	6,100 (45.5)	
Other^a^	24,443 (7.7)	22,691 (7.5)	1,752 (13.1)	
Townsend deprivation index	−2.3 (−3.7, 0.2)	−2.3 (−3.7, 0.2)	−1.6 (−3.3, 1.4)	<0.001
Smoking status				
Non-smoker	181,641 (57.2)	173,772 (57.1)	7,869 (58.7)	<0.001
Ever-smoker	104,336 (32.8)	100,080 (32.9)	4,256 (31.8)	
Current smoker	31,651 (10.0)	30,380 (10.0)	1,271 (9.5)	
Alcohol consumption				<0.001
Never or special occasions only	55,982 (17.6)	51,904 (17.1)	4,078 (30.4)	
1−3 times per month	35,318 (11.1)	33,849 (11.1)	1,469 (11.0)	
1−4 times per week	160,504 (50.5)	154,862 (50.9)	5,642 (42.1)	
Daily or almost daily	65,824 (20.7)	63,617 (20.9)	2,207 (16.5)	
Healthy diet				<0.001
No	63,494 (20.0)	60,577 (19.9)	2,917 (21.8)	
Yes	254,134 (80.0)	243,655 (80.1)	10,479 (78.2)	
Regular exercise				<0.001
No	139,421 (43.9)	132,177 (43.4)	7,244 (54.1)	
Yes	178,207 (56.1)	172,055 (56.6)	6,152 (45.9)	
Sleep duration in hours	7.0 (7.0, 8.0)	7.0 (7.0, 8.0)	7.0 (6.0, 8.0)	0.334
Body mass index in kg/m^2^	26.5 (23.9, 29.5)	26.4 (23.9, 29.5)	26.8 (24.1, 30.2)	<0.001
Waist circumference in cm	89.0 (80.0, 98.0)	89.0 (79.0, 98.0)	90.0 (80.0, 99.0)	<0.001
FEV1 z-score	−0.1 (−0.7, 0.6)	−0.1 (−0.7, 0.7)	−0.7 (−1.2, −0.1)	<0.001
FVC z-score	−0.1 (−0.7, 0.6)	−0.1 (−0.7, 0.7)	−0.7 (−1.2, −0.0)	<0.001
PEF z-score	−0.1 (−0.7, 0.6)	−0.1 (−0.7, 0.7)	−0.6 (−1.2, −0.0)	<0.001
FEV1/FVC ratio (%)	77.3 (73.5, 80.7)	77.4 (73.5, 80.7)	76.8 (72.5, 80.4)	<0.001
Lung function score	−0.1 (−0.7, 0.6)	−0.1 (-0.6, 0.6)	−0.7 (−1.1, −0.1)	<0.001

FEV1, forced expiratory volume in one second; FVC, forced vital capacity; PEF, peak expiratory flow.

The data are expressed as numbers and percentages for categorical variables and medians and inter-quartile range (IQR) for continuous variables. The P values were generated using χ^2^ and Kruskal–Wallis test for categorical and continuous variables, respectively.

a Other includes unemployed, student, volunteer, and so on.

### 3.2 Associations of probable sarcopenia with lung function and incident respiratory disease

**Fig. 1:**
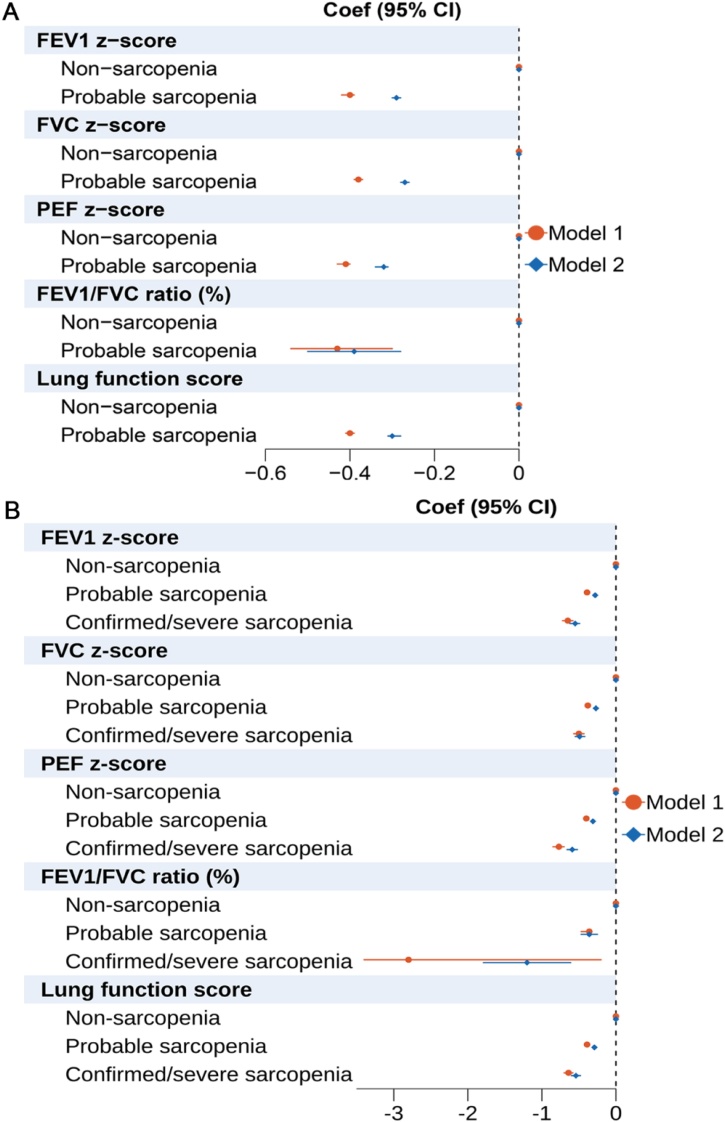
Cross-sectional associations of sarcopenia with lung function. (A) Associations of probable sarcopenia with lung function. (B) Associations of probable sarcopenia, and confirmed/severe sarcopenia with lung function. Model 1 was adjusted for age and sex. Model 2 was further adjusted for ethnicity, educational attainment, occupational status, Townsend deprivation index, smoking status, alcohol consumption, healthy diet, regular exercise, sleep duration, body mass index, and waist circumference based on model 1. The dot indicates coefficient (i.e., β), and the line indicates corresponding 95% CI. Lung function score was calculated by averaging z-scores of FEV1, FVC, and PEF. FEV1, forced expiratory volume in one second; FVC, forced vital capacity; PEF, peak expiratory flow; CI, confidence interval.

**Fig. 2:**
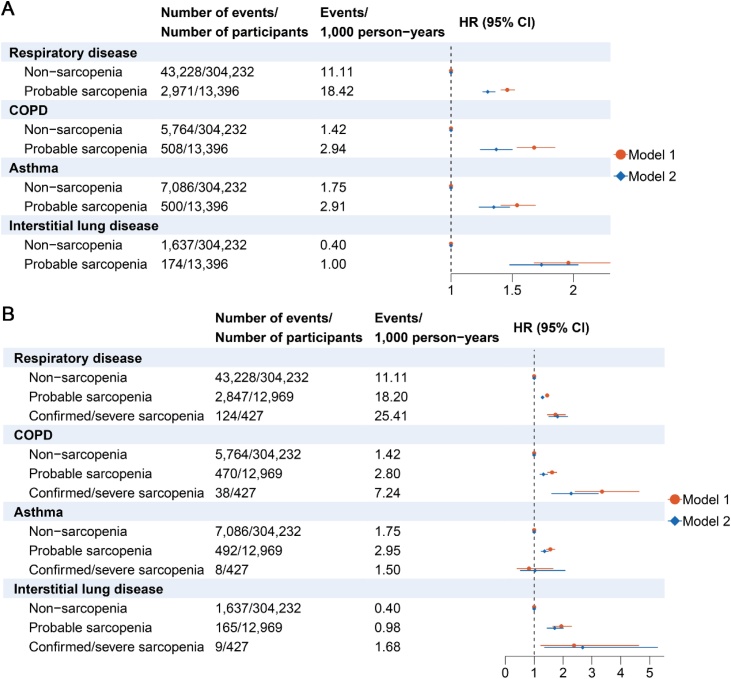
Associations of sarcopenia with the risk of incident respiratory disease. (A) Associations of probable sarcopenia with the risk of incident respiratory disease. (B) Associations of probable sarcopenia, and confirmed/severe sarcopenia with the risk of incident respiratory disease. Model 1 was adjusted for age and sex. Model 2 was further adjusted for ethnicity, educational attainment, occupational status, Townsend deprivation index, smoking status, alcohol consumption, healthy diet, regular exercise, sleep duration, body mass index, and waist circumference based on model 1. The dot indicates HR, and the line indicates 95% CI. HR, hazard ratio; CI, confidence interval; COPD, chronic obstructive pulmonary disease.

Those with probable sarcopenia tended to have poorer lung function (Supplementary Fig. S2). Compared with participants who were non-sarcopenia, those with probable sarcopenia had a decrease in FEV1 z-score (β = −0.29; 95% CI: −0.30, −0.28; P < 0.001), FVC z-score (β = −0.27; 95% CI: -0.28, -0.26; P < 0.001), PEF z-score (β = −0.32; 95% CI: −0.34, −0.31; P < 0.001), FEV1/FVC ratio (β = −0.39%; 95% CI: −0.50%, −0.28%; P < 0.001), and overall lung function score (β = −0.30; 95% CI: −0.31, −0.28; P < 0.001) after adjusting for age, sex, ethnicity, educational attainment, occupational status, TDI, smoking status, alcohol consumption, healthy diet, regular exercise, sleep duration, BMI, and WC (Fig. 1A, and Supplementary Table S1). The Kaplan–Meier curves demonstrated the higher cumulative incidences for incident respiratory disease in participants with probable sarcopenia (Supplementary Fig. S3). The 10-year unadjusted cumulative incidences of respiratory disease were 9.00% (95% CI: 8.90%, 9.10%), and 15.14% (95% CI: 14.53%, 15.75%) for non-sarcopenic and probable sarcopenic participants, respectively (Supplementary Table S2). For participants who had probable sarcopenia compared to non-sarcopenic participants, the absolute risk difference was 6.14% (95% CI: 5.47%, 6.82%). Associations of probable sarcopenia with incident respiratory disease are shown in Fig. 2A. In the age- and sex-adjusted model, probable sarcopenia was significantly associated with higher risks of incident respiratory disease (all P < 0.001). After further adjusting for additional covariates, these associations remained statistically significant. When comparing probable sarcopenia participants with their non-sarcopenia counterparts, the multivariable-adjusted HRs were 1.30 (95% CI: 1.26, 1.36; P < 0.001) for respiratory disease, 1.37 (95% CI: 1.24, 1.50; P < 0.001) for COPD, 1.35 (95% CI: 1.23, 1.48; P < 0.001) for asthma, and 1.74 (95% CI: 1.48, 2.04; P < 0.001) for ILD (Supplementary Table S3).

When classifying participants into non-sarcopenia, probable sarcopenia, and confirmed/severe sarcopenia, we observed significant associations of probable sarcopenia, and confirmed/severe sarcopenia with lung function (Fig. 1B, and Supplementary Table S4). Similarly, higher cumulative incidences of respiratory disease were observed for those with probable sarcopenia, and confirmed/severe sarcopenia (except asthma) (Supplementary Fig. S4, and Table S5). Also, significant associations were found for incident respiratory disease (Fig. 2B, and Supplementary Table S6), except for the association between confirmed/severe sarcopenia and incident asthma. For instance, in the multivariable adjusted model, compared with non-sarcopenic participants, the risk of respiratory disease, COPD, and ILD for those with confirmed/severe sarcopenia increased by 81% (95% CI: 1.51, 2.16; P < 0.001), 128% (95% CI: 1.61, 3.22; P < 0.001), and 168% (95% CI: 1.36, 5.27; P = 0.004), respectively.

**Fig. 3:**
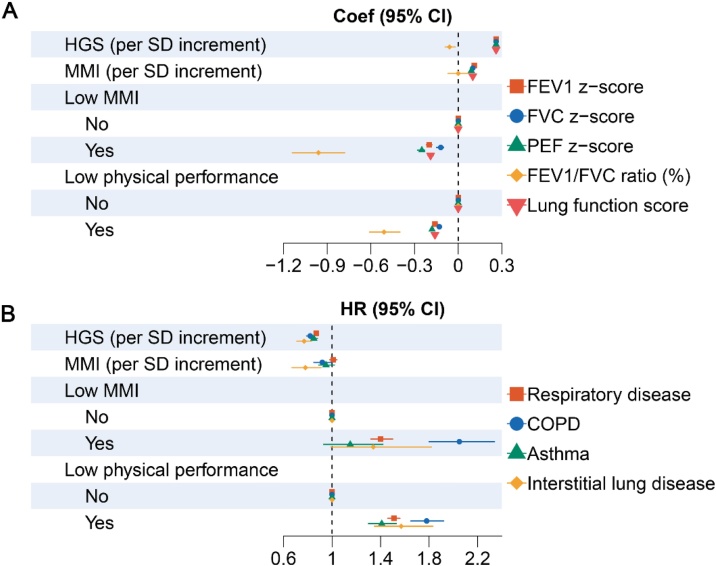
Associations of sarcopenia components with lung function and the risk of incident respiratory disease. (A) Cross-sectional associations of sarcopenia components with lung function. (B) Prospective associations of sarcopenia components with the risk of incident respiratory disease. The models were adjusted for age, sex, ethnicity, educational attainment, occupational status, Townsend deprivation index, smoking status, alcohol consumption, healthy diet, regular exercise, sleep duration, body mass index, and waist circumference. The dot indicates coefficient (or HR), and the line indicates 95% CI. CI, confidence interval; HGS, handgrip strength; SD, standard deviation; MMI, muscle mass index; FEV1, forced expiratory volume in one second; FVC, forced vital capacity; PEF, peak expiratory flow; HR, hazard ratio; COPD, chronic obstructive pulmonary disease.

Almost all sarcopenia components were significantly associated with lung function and incident respiratory disease (Fig. 3, and Supplementary Tables S7-S8). Particularly, per SD-increment in HGS and low physical performance showed consistent associations with all lung function indicators and incident respiratory disease (all P < 0.05). When stratifying by age, and sex, the results remained robust in subgroups (Supplementary Tables S9-S12). Specifically, associations of probable sarcopenia with FVE1 z-score, FVC z-score, PEF z-score, overall lung function score, and incident respiratory disease and COPD were more pronounced in middle-aged adults and males. Furthermore, the significant associations of probable sarcopenia with incident respiratory disease remained unchanged when further adjusting for z-scores of FEV1, FVC, and PEF at baseline (Supplementary Table S13), and excluding the participants with less than 2 years of follow-up (Supplementary Table S14).

### 3.3 Mediation analyses of inflammatory markers and metabolites on the associations of probable sarcopenia with incident respiratory disease

**Fig. 4:**
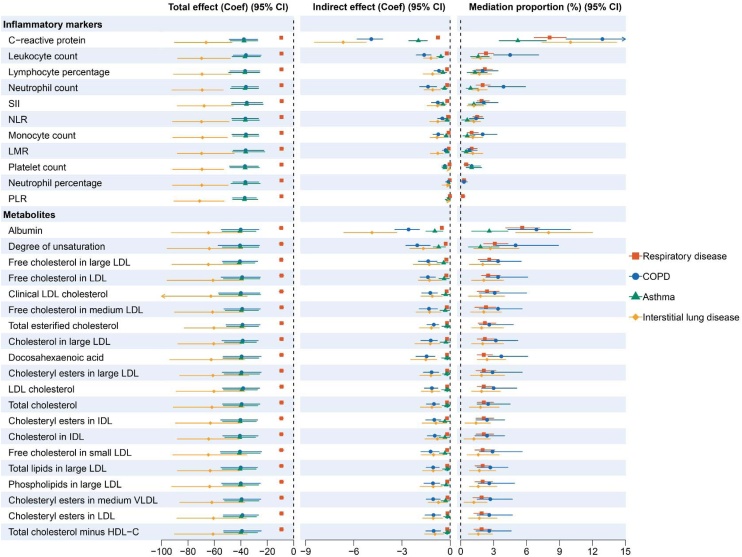
Mediation proportions of probable sarcopenia in respiratory disease attributed to inflammatory markers and metabolites. The forest plot shows the mediation results of all inflammatory markers, and top 20 metabolites that have significant effect on respiratory disease. For the mediation proportion, we only presented the significant results. The models were adjusted for age, sex, ethnicity, educational attainment, occupational status, Townsend deprivation index, smoking status, alcohol consumption, healthy diet, regular exercise, sleep duration, body mass index, and waist circumference. The dot indicates total effect (i.e., β), indirect effect (i.e., β), and mediation proportion, respectively; the line indicates corresponding 95% CI. CI, confidence interval; SII, systemic immune-inflammation index; NLR, neutrophil-to-lymphocyte ratio; LMR, lymphocyte-to-monocyte ratio; PLR, platelet-to-lymphocyte ratio; LDL, low-density lipoprotein; IDL, Intermediate Density Lipoprotein; VLDL, very low-density lipoprotein; HDL-C, high-density lipoprotein cholesterol; COPD, chronic obstructive pulmonary disease.

As shown in Supplementary Tables S15-S16, probable sarcopenia was significantly associated with 11 inflammatory markers (e.g., CRP, SII, and NLR), and 125 metabolites. In addition, there were 11 inflammatory markers and 134 metabolites that showed significant association with incident respiratory disease (Supplementary Tables S17 and S18). Finally, we conducted mediation analyses for 11 inflammatory markers and 86 metabolites, and found that the associations of probable sarcopenia with incident respiratory disease were partially mediated by most of these inflammatory markers and metabolites (Supplementary Tables S19-S26). Among all inflammatory markers and metabolites, CRP (respiratory disease: 8.1%; COPD: 12.9%; asthma: 5.2%; ILD: 10.0%) and albumin (respiratory disease: 5.6%; COPD: 6.9%; asthma: 2.6%; ILD: 8.0%) appeared to partially mediate the most of respiratory disease risk for those who were probable sarcopenia, respectively (Fig. 4).

## 4 Discussion

In this large population-based cohort study, we comprehensively explored the associations of sarcopenia with lung function and incident respiratory disease. Our results suggested that sarcopenia was significantly associated with poorer lung function, as well as increased risk of respiratory disease, including its major subtypes (i.e., COPD, asthma, and ILD). Meanwhile, we also observed that individual components of sarcopenia, particularly HGS and low physical performance, act as pronounced predictors for lung function and incident respiratory disease. Furthermore, we demonstrated that inflammatory markers and metabolites partially mediated the associations between probable sarcopenia and incident respiratory disease, revealing the potential pathways that link sarcopenia to respiratory disease.

Cumulative evidence suggests that sarcopenia is positively associated with poorer lung function in individuals with COPD [15] or idiopathic pulmonary fibrosis [29]. For instance, a recent meta-analysis involving 11 studies showed that participants with sarcopenia had poorer FEV1 than non-sarcopenic participants among those with COPD [15]. The present study extended such estimations into general adults, and observed consistent results. This strengthened the evidence that deterioration in muscle mass and strength may modify the risk of lung function impairment, aligning with observations from previous research that demonstrated significant associations between sarcopenia components (e.g., HGS, MMI) and lung function, although with a relatively small sample size [[8], [9], [10],^30^]. Lung function indicators are generally used as outcome measures for risk estimation, as lung function impairment typically precedes the onset of incident respiratory disease. From the aspect of health implications, our study supports the implementation of sarcopenia assessment to identify those who are at high risk of respiratory disease before their occurrence, offering a potential window of opportunity for early intervention.

To our awareness, research exploring the association of sarcopenia with respiratory disease remains limited [12,14]. These two prospective studies had short follow-up (<7 years) with a primary focus on general respiratory disease [12,14], although one study included COPD [12]. Additionally, a recent Mendelian randomization analysis provided evidence of a causal effect of sarcopenia on 11 respiratory diseases, such as COPD and asthma [31]. Based on a large population with a median follow-up of 14 years, our study identified consistent detrimental effects of sarcopenia on respiratory disease and its subtypes, including COPD, asthma, and ILD. The robust results across different population subgroups in the present study further strengthen their public implications. Although still observational evidence, these findings echo the need for more actions to prevent and manage sarcopenia [32], which may further promote respiratory health.

Importantly, the findings of this study suggested that middle-aged and older adults in the early stage of sarcopenia might be the potential subgroup to be targeted. Firstly, despite the relatively low prevalence of probable sarcopenia (4.1%) and confirmed/severe sarcopenia (0.1%), both conditions showed a positive association with lung function and incident respiratory disease. Second, HGS, a practical and inexpensive way used to define probable sarcopenia, was also found to be linearly associated with lung function and incident respiratory disease. Based on these observations, we recommend integrating HGS measurements into routine clinical workflow in respiratory clinics, which could help identify high-risk individuals who might otherwise be missed, facilitating timely interventions to maintain respiratory health. Notably, there has been growing research on nonpharmacological interventions (e.g., exercise, and nutrition supplementation) targeting sarcopenia, with studies observing significant improvements in skeletal muscle strength [[33], [34], [35]]. Taking advantage of the potential intervention window to successfully delay or reverse sarcopenia may therefore hold great significance for protecting respiratory health. Nevertheless, the cost-effectiveness of interventional programs targeting sarcopenia remains unclear. More interventional studies are expected to offer promising avenues for incorporating sarcopenia management into respiratory practice.

Our study provides additional evidence regarding the mechanisms underlying the associations between sarcopenia and respiratory disease. Existing evidence indicates that sarcopenia is associated with higher levels of CRP — a well-recognized clinical marker of acute systemic inflammation [20,36]. Inflammation represents the primary pathological feature of most respiratory diseases and, critically, contributes to disease progression and elevated mortality [[21], [22], [23]]. Our observation that inflammatory markers partially mediate the sarcopenia-respiratory disease association, with CRP accounting for the largest mediation proportion, supports the underlying inflammatory pathway. Furthermore, several studies have reported that metabolite levels (e.g., isoleucine, lysine, and carnitine) differed by sarcopenia status [25,37]. Metabolic alterations have also been documented in those with respiratory disease [26,38,39]. In the present study, we further demonstrated that sarcopenia may lead to respiratory disease partially through changes in metabolites. These observations highlight that nutritional support is essential for sarcopenia management, which may help mitigate the incidence of respiratory disease. Of particular interest, albumin accounted for the largest proportion of the mediating effect. Albumin is not merely a marker of malnutrition [40], but also an indicator of systemic inflammatory burden [41]. A high CRP/low albumin phenotype signifies a state of ongoing or heightened systemic inflammation. On the one hand, this state increases pulmonary microvascular endothelial permeability, promoting alveolar edema and facilitating neutrophil entry into the airspace [42]. On the other hand, the primed inflammatory environment can induce oxidative stress, directly damaging alveolar epithelial cells, particularly type II pneumocytes that are critical for regeneration [43]. Ultimately, persistent systemic inflammation creates a vicious cycle that predisposes the lung parenchyma to injury or impaired repair. Overall, the results suggest that regulating inflammation and metabolic disturbance may help mitigate the adverse impact of sarcopenia on respiratory health, though our findings still require further validation.

Major strengths of the present study included the large sample size of UK middle-aged and older participants, the prospective design with a long follow-up period, and a comprehensive demonstration of the associations of sarcopenia and its components with lung function and incident respiratory disease, as well as potential inflammatory and metabolic pathways. Nevertheless, some limitations should be noted. First, dual-energy X-ray absorptiometry is the most effective method for evaluating lean or fat skeletal muscle mass due to its accuracy and repeatability [4]. In the UKB, muscle mass was assessed using bioelectrical impedance analysis, which showed good correlation with dual-energy X-ray absorptiometry measurements [7], and made a lot of sense in many clinics or community settings. Second, physical performance was assessed through self-reported walking pace, which may lead to recall bias. Due to the small sample size of the confirmed/severe subgroup, the misclassified cases could significantly change the observed effect sizes or widen confidence intervals, potentially masking genuine associations or creating spurious ones. Thus, the associations of confirmed/severe sarcopenia with outcomes should be interpreted with caution. Despite the strong correlation of self-reported walking pace with gait speed measurement [44], further studies with objectively measured gait speed were warranted to verify our findings. Third, the majority of UKB participants were White, and they tended to be healthier [45], so our sample was not representative of the general UK population. While the robustness of our results has been confirmed by several sensitivity analyses, more research on other populations is still needed. Finally, although we considered many covariates in our study, there may be other neglected confounding factors.

## 5 Conclusions

Sarcopenia was associated with poorer lung function and increased risks of incident respiratory disease. Additionally, we demonstrated the underlying pathway that links sarcopenia to respiratory disease through inflammation and metabolism. The findings underscore the importance of preventive programs targeting sarcopenia, even probable sarcopenia, aiming at regulating inflammation and metabolism, and further achieving better respiratory health.

## CRediT authorship contribution statement

**Xingqi Cao:** Conceptualization, Data curation, Formal analysis, Funding acquisition, Methodology, Software, Visualization, Writing - original draft. **Xixuan Cai:** Methodology, Writing - original draft. **Jia Zhang:** Methodology, Writing - review & editing. **Jingkai Yan:** Software, Writing - review & editing. **Yanjie Zhao:** Methodology, Writing - review & editing. **Xueqing Jia:** Methodology, Writing - review & editing. **Kaili Sun:** Methodology, Writing - review & editing. **Zuyun Liu:** Conceptualization, Data curation, Funding acquisition, Methodology, Supervision, Writing - review & editing. **Liying Chen:** Conceptualization, Methodology, Supervision, Writing - review & editing.

## Ethics approval and consent to participate

UK Biobank was approved by the North West Multi-center Research Ethics Committee (11/NW/0382). Informed consent was obtained from all participants.

## Declaration of Generative AI and AI-assisted technologies in the writing process

The authors declare that generative AI and AI-assisted technologies were not used in the writing, analysis, or preparation of this manuscript.

## Funding

This work was supported by grants from the National Key R&D Program of China (2025YFC3608000), 10.13039/501100001809National Natural Science Foundation of China (72374180, 82504422), “Pioneer” and “Leading Goose” R&D Programs of Zhejiang Province (2026C02A1147, 2025C02104), Zhejiang Key Laboratory of Intelligent Preventive Medicine (2020E10004), and Zhejiang University School of Public Health Interdisciplinary Research Innovation Team Development Project. The funders had no role in the study design; data collection, analysis, or interpretation; in the writing of the report; or in the decision to submit the article for publication.

## Data availability

The data that support the findings of this study are openly available in UK Biobank at [https://www.ukbiobank.ac.uk/].

## Declaration of competing interest

The authors declare no conflicts of interest.
