# Supplementary material for: Associations of sarcopenia with the risk of incident respiratory disease and the role of inflammation and metabolism: a prospective cohort study
Source: J Nutr Health Aging. 2026 Jul 2;30(8):100919. doi: 10.1016/j.jnha.2026.100919 (PMC13352055; doi:10.1016/j.jnha.2026.100919)
Supplement: Supplementary file 1 [file mmc1.docx]

**Supplementary Materials**

[Supplementary Fig. S1. Flow chart of the analytic sample. 3](#_Toc231309049)

[Supplementary Fig. S2. Distribution of lung function by sarcopenia. 4](#_Toc231309050)

[Supplementary Fig. S3. Kaplan-Meier curves of cumulative incidence of respiratory disease by two categories of sarcopenia. 5](#_Toc231309051)

[Supplementary Fig. S4. Kaplan-Meier curves of cumulative incidence of respiratory disease by three categories of sarcopenia. 6](#_Toc231309052)

[Supplementary Table S1. Cross-sectional associations of probable sarcopenia with lung function at baseline. 7](#_Toc231309053)

[Supplementary Table S2. Cumulative incidences and risk differences at 5- and 10- year of respiratory disease among two categories of sarcopenia. 8](#_Toc231309054)

[Supplementary Table S3. Associations of probable sarcopenia with the risk of respiratory disease. 9](#_Toc231309055)

[Supplementary Table S4. Cross-sectional associations of probable and confirmed/severe sarcopenia with lung function at baseline. 10](#_Toc231309056)

[Supplementary Table S5. Cumulative incidences and risk differences at 5- and 10- year of respiratory disease among three categories of sarcopenia. 11](#_Toc231309057)

[Supplementary Table S6. Associations of probable and confirmed/severe sarcopenia with the risk of respiratory disease (N=317,628). 12](#_Toc231309058)

[Supplementary Table S7. Cross-sectional associations of sarcopenia component with lung function at baseline. 13](#_Toc231309059)

[Supplementary Table S8. Associations of sarcopenia component with the risk of respiratory disease. 15](#_Toc231309060)

[Supplementary Table S9. Cross-sectional associations of probable sarcopenia with lung function level at baseline stratified by age categories. 17](#_Toc231309061)

[Supplementary Table S10. Cross-sectional associations of probable sarcopenia with lung function at baseline stratified by sex. 18](#_Toc231309062)

[Supplementary Table S11. Associations of probable sarcopenia with the risk of respiratory disease stratified by age categories. 19](#_Toc231309063)

[Supplementary Table S12. Associations of probable sarcopenia with the risk of respiratory disease stratified by sex. 20](#_Toc231309064)

[Supplementary Table S13. Associations of probable sarcopenia with the risk of respiratory disease after further adjusting for lung function at baseline. 21](#_Toc231309065)

[Supplementary Table S14. Associations of probable sarcopenia with risk of respiratory disease after excluding those who were followed up for less than 2 years. 22](#_Toc231309066)

[Supplementary Table S15. Associations of probable sarcopenia with blood inflammatory markers. 23](#_Toc231309067)

[Supplementary Table S16. Associations of probable sarcopenia with metabolites (N=166,721). 24](#_Toc231309068)

[Supplementary Table S17. Associations of inflammatory markers with the risk of respiratory disease. 29](#_Toc231309069)

[Supplementary Table S18. Associations of metabolites with the risk of respiratory disease (N=166,721). 30](#_Toc231309070)

[Supplementary Table S19. Mediation proportion of probable sarcopenia in respiratory disease attributed to blood inflammatory markers. 38](#_Toc231309071)

[Supplementary Table S20. Mediation proportion of probable sarcopenia in COPD attributed to blood inflammatory markers. 39](#_Toc231309072)

[Supplementary Table S21. Mediation proportion of probable sarcopenia in asthma attributed to blood inflammatory markers. 40](#_Toc231309073)

[Supplementary Table S22. Mediation proportion of probable sarcopenia in interstitial lung disease attributed to blood inflammatory markers. 41](#_Toc231309074)

[Supplementary Table S23. Mediation proportion of probable sarcopenia in respiratory disease attributed to metabolites (N=166,721). 42](#_Toc231309075)

[Supplementary Table S24. Mediation proportion of probable sarcopenia in COPD attributed to metabolites (N=166,721). 45](#_Toc231309076)

[Supplementary Table S25. Mediation proportion of probable sarcopenia in asthma attributed to metabolites (N=166,721). 48](#_Toc231309077)

[Supplementary Table S26. Mediation proportion of probable sarcopenia in interstitial lung disease attributed to metabolites (N=166,721). 51](#_Toc231309078)


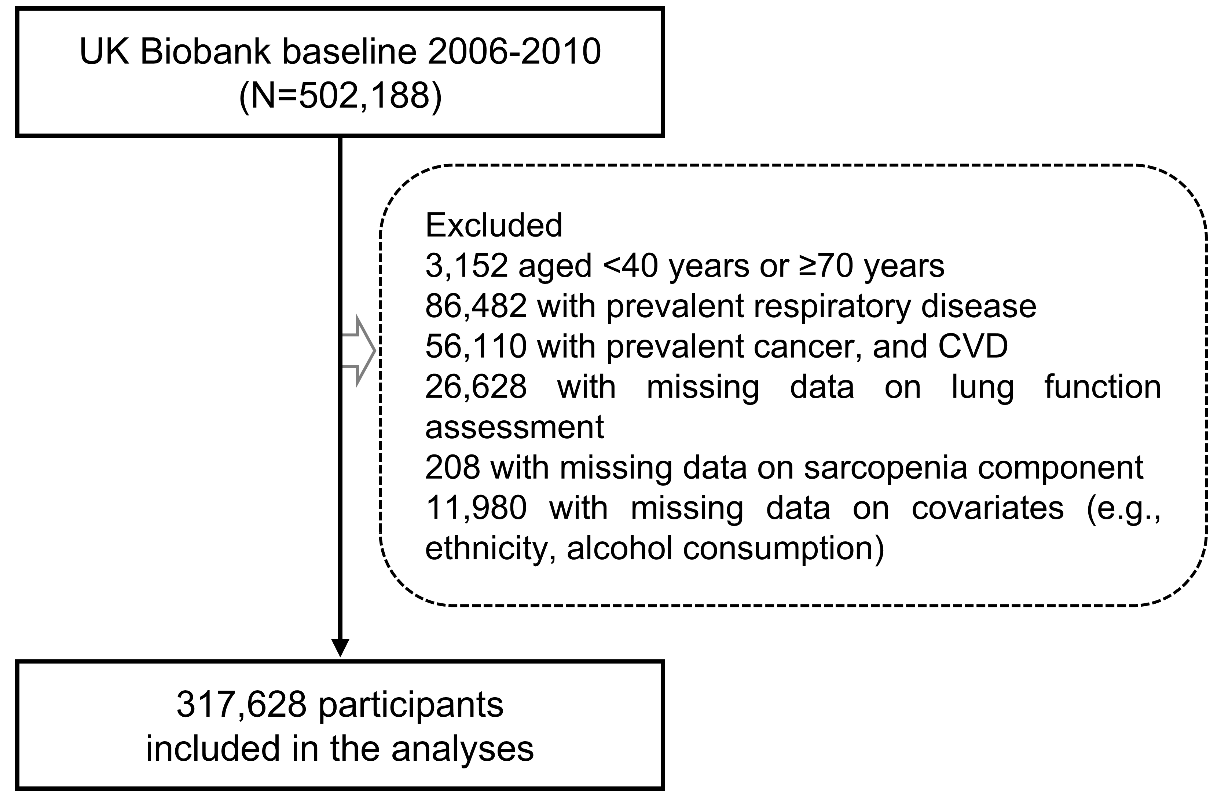


# **Supplementary Fig. S1. Flow chart of the analytic sample.**

CVD, cardiovascular disease.


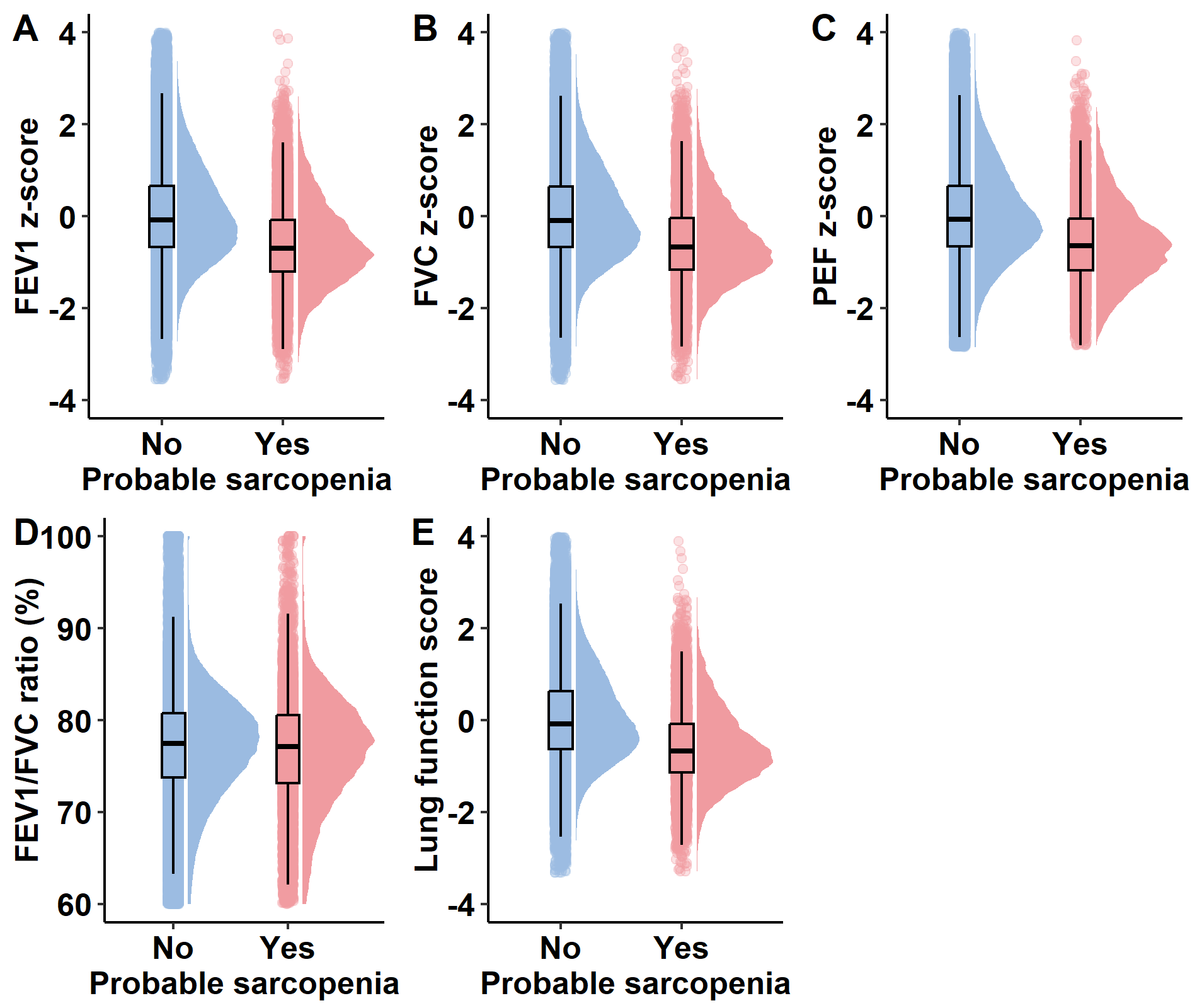


# **Supplementary Fig. S2. Distribution of lung function by sarcopenia.**

Distribution of FEV1 z-score (A), FVC z-score (B), PEF z-score (C), FEV1/FVC ratio (D), and lung function score (E) by sarcopenia status. FEV1, forced expiratory volume in one second; FVC, forced vital capacity; PEF, peak expiratory flow; CI, confidence interval.


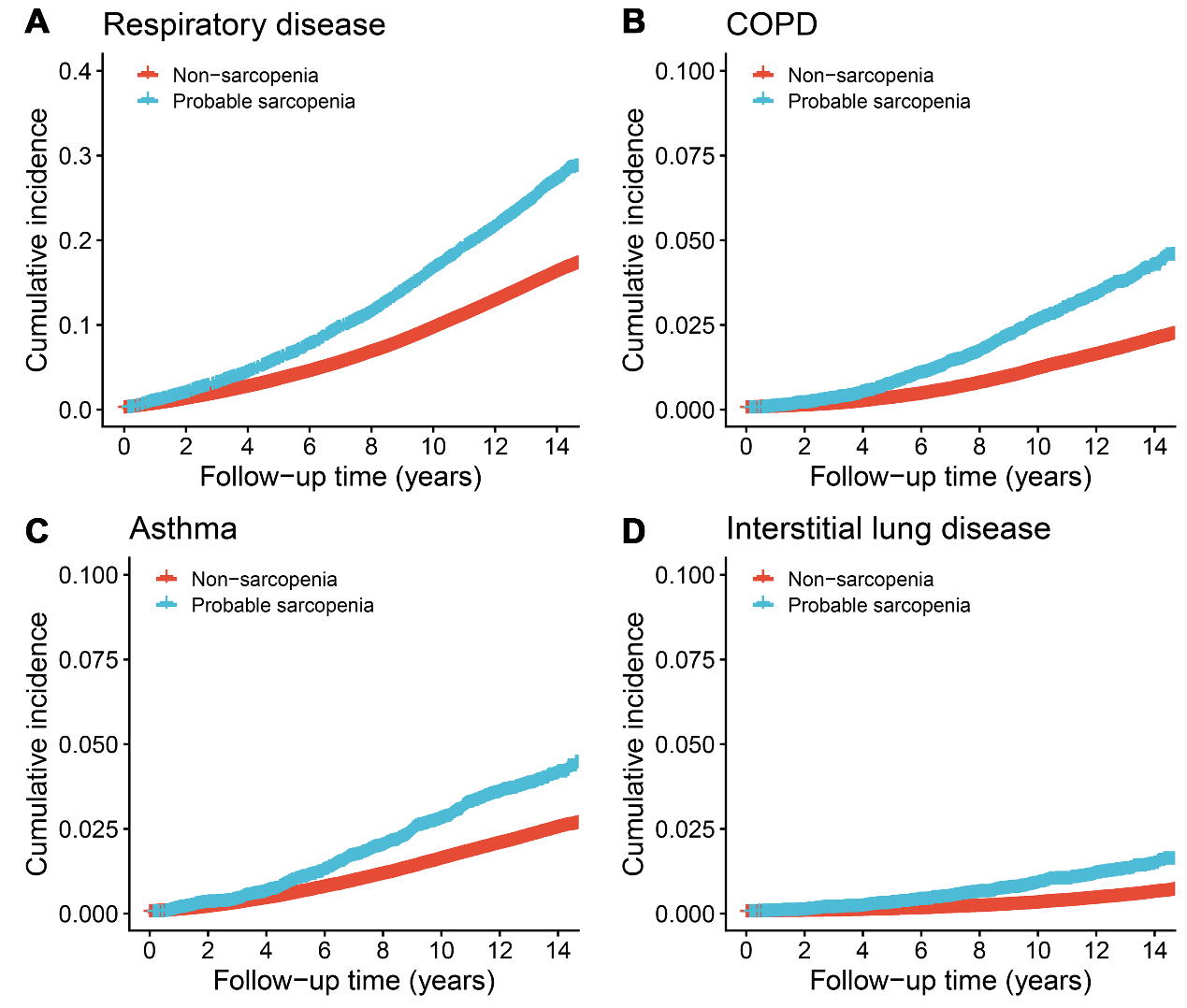


# **Supplementary Fig. S3. Kaplan-Meier curves of cumulative incidence of respiratory disease by two categories of sarcopenia.**

Kaplan-Meier curves of cumulative incidence of respiratory disease (A), COPD (B), asthma (C), and interstitial lung disease (D) by two categories of sarcopenia. The models were unadjusted. COPD, chronic obstructive pulmonary disease.


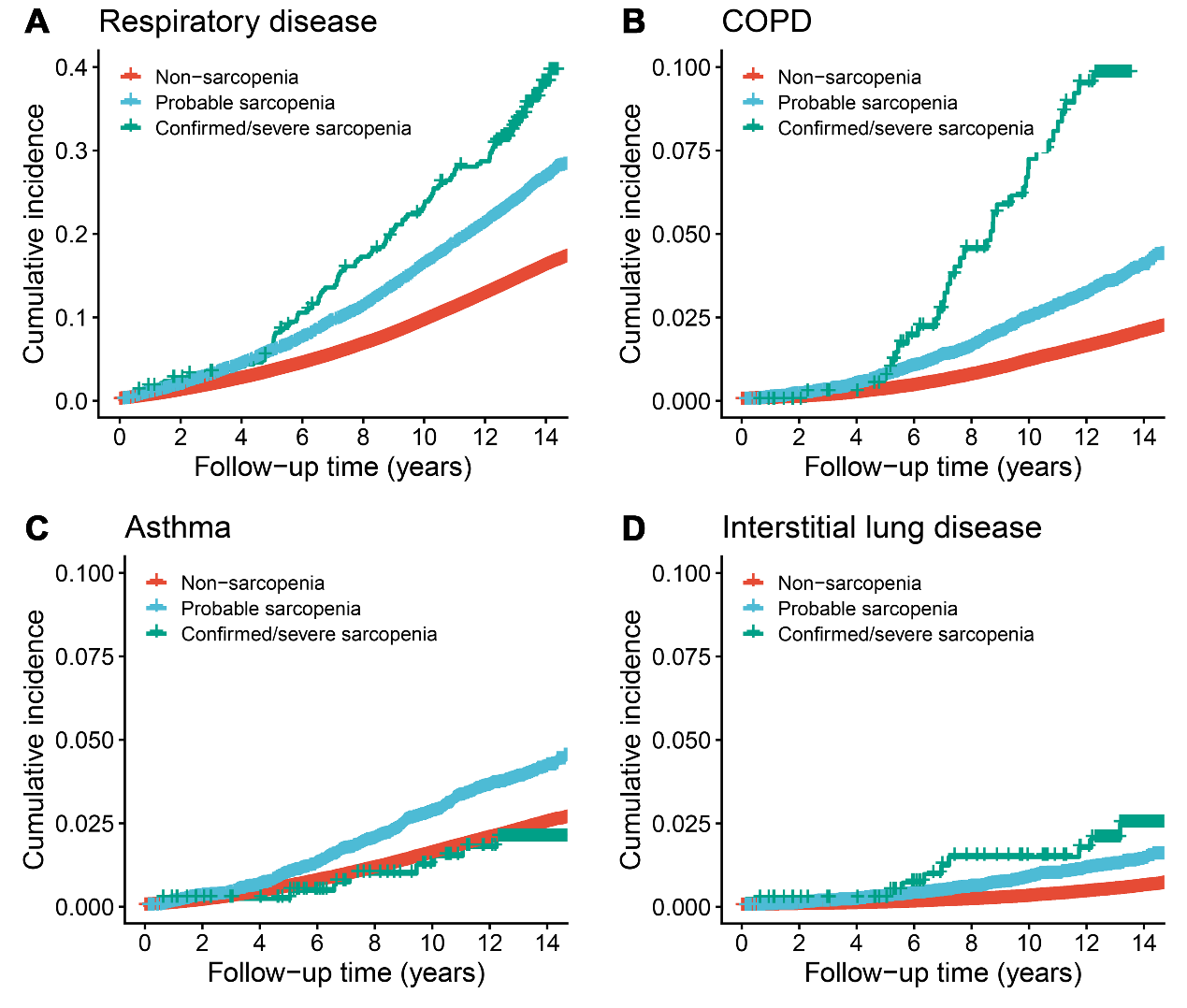


# **Supplementary Fig. S4. Kaplan-Meier curves of cumulative incidence of respiratory disease by three categories of sarcopenia.**

Kaplan-Meier curves of cumulative incidence of respiratory disease (A), COPD (B), asthma (C), and interstitial lung disease (D) by three categories of sarcopenia. The models were unadjusted. COPD, chronic obstructive pulmonary disease.

# **Supplementary Table S1. Cross-sectional associations of probable sarcopenia with lung function at baseline.**

|  | **Model 1** | | **Model 2** | |
| --- | --- | --- | --- | --- |
|  | **β (95% CI)** | **P** | **β (95% CI)** | **P** |
| **FEV1 z-score** |  |  |  |  |
| Non-sarcopenia | Ref. | — | Ref. | — |
| Probable sarcopenia | -0.40 (-0.42, -0.39) | <0.001 | -0.29 (-0.30, -0.28) | <0.001 |
| **FVC z-score** |  |  |  |  |
| Non-sarcopenia | Ref. | — | Ref. | — |
| Probable sarcopenia | -0.38 (-0.39, -0.37) | <0.001 | -0.27 (-0.28, -0.26) | <0.001 |
| **PEF z-score** |  |  |  |  |
| Non-sarcopenia | Ref. | — | Ref. | — |
| Probable sarcopenia | -0.41 (-0.43, -0.40) | <0.001 | -0.32 (-0.34, -0.31) | <0.001 |
| **FEV1/FVC ratio (%)** |  |  |  |  |
| Non-sarcopenia | Ref. | — | Ref. | — |
| Probable sarcopenia | -0.43 (-0.54, -0.32) | <0.001 | -0.39 (-0.50, -0.28) | <0.001 |
| **Lung function score** |  |  |  |  |
| Non-sarcopenia | Ref. | — | Ref. | — |
| Probable sarcopenia | -0.40 (-0.41, -0.39) | <0.001 | -0.30 (-0.31, -0.28) | <0.001 |

CI, confidence interval; FEV1, forced expiratory volume in one second; FVC, forced vital capacity; PEF, peak expiratory flow.

Model 1 was adjusted for age, and sex.

Model 2 was further adjusted for ethnicity, educational attainment, occupational status, Townsend deprivation index, smoking status, alcohol consumption, healthy diet, regular exercise, sleep duration, body mass index, and waist circumference based on model 1.

# **Supplementary Table S2. Cumulative incidences and risk differences at 5- and 10- year of respiratory disease among two categories of sarcopenia.**

|  | **Cumulative incidence (%) (95% CI)** | | **Risk difference (%) (95% CI)** | |
| --- | --- | --- | --- | --- |
|  | **5-year** | **10-year** | **5-year** | **10-year** |
| **Respiratory disease** |  |  |  |  |
| Non-sarcopenia | 3.29 (3.23, 3.36) | 9.00 (8.90, 9.10) | Ref. | Ref. |
| Probable sarcopenia | 5.64 (5.25, 6.04) | 15.14 (14.53, 15.75) | 2.35 (1.92, 2.79) | 6.14 (5.47, 6.82) |
| **COPD** |  |  |  |  |
| Non-sarcopenia | 0.29 (0.27, 0.31) | 1.14 (1.11, 1.18) | Ref. | Ref. |
| Probable sarcopenia | 0.72 (0.58, 0.87) | 2.55 (2.28, 2.82) | 0.43 (0.28, 0.58) | 1.41 (1.13, 1.69) |
| **Asthma** |  |  |  |  |
| Non-sarcopenia | 0.55 (0.52, 0.58) | 1.55 (1.51, 1.60) | Ref. | Ref. |
| Probable sarcopenia | 0.96 (0.79, 1.12) | 2.73 (2.45, 3.00) | 0.41 (0.24, 0.58) | 1.17 (0.88, 1.46) |
| **Interstitial lung disease** | |  |  |  |
| Non-sarcopenia | 0.07 (0.06, 0.08) | 0.26 (0.24, 0.28) | Ref. | Ref. |
| Probable sarcopenia | 0.27 (0.18, 0.36) | 0.85 (0.69, 1.01) | 0.20 (0.11, 0.29) | 0.59 (0.43, 0.75) |

CI, confidence interval; COPD, chronic obstructive pulmonary disease.

The models were unadjusted.

# **Supplementary Table S3. Associations of probable sarcopenia with the risk of respiratory disease.**

|  | **No of events/No of participants** | **Events/1,000 person-years** | **Model 1** | | **Model 2** | |
| --- | --- | --- | --- | --- | --- | --- |
|  |  |  | **HR (95% CI)** | **P** | **HR (95% CI)** | **P** |
| **Respiratory disease** |  |  |  |  |  |  |
| Non-sarcopenia | 43,228/304,232 | 11.11 | Ref. | — | Ref. | — |
| Probable sarcopenia | 2,971/13,396 | 18.42 | 1.46 (1.41, 1.52) | <0.001 | 1.30 (1.26, 1.36) | <0.001 |
| **COPD** |  |  |  |  |  |  |
| Non-sarcopenia | 5,764/304,232 | 1.42 | Ref. | — | Ref. | — |
| Probable sarcopenia | 508/13,396 | 2.94 | 1.68 (1.54, 1.85) | <0.001 | 1.37 (1.24, 1.50) | <0.001 |
| **Asthma** |  |  |  |  |  |  |
| Non-sarcopenia | 7,086/304,232 | 1.75 | Ref. | — | Ref. | — |
| Probable sarcopenia | 500/13,396 | 2.91 | 1.54 (1.41, 1.69) | <0.001 | 1.35 (1.23, 1.48) | <0.001 |
| **Interstitial lung disease** |  |  |  |  |  |  |
| Non-sarcopenia | 1,637/304,232 | 0.40 | Ref. | — | Ref. | — |
| Probable sarcopenia | 174/13,396 | 1.00 | 1.96 (1.68, 2.30) | <0.001 | 1.74 (1.48, 2.04) | <0.001 |

HR, hazard ratio; CI, confidence interval; COPD, chronic obstructive pulmonary disease.

Model 1 was adjusted for age, and sex.

Model 2 was further adjusted for ethnicity, educational attainment, occupational status, Townsend deprivation index, smoking status, alcohol consumption, healthy diet, regular exercise, sleep duration, body mass index, and waist circumference based on model 1.

# **Supplementary Table S4. Cross-sectional associations of probable and confirmed/severe sarcopenia with lung function at baseline.**

|  | **Model 1** | | **Model 2** | |
| --- | --- | --- | --- | --- |
|  | **β (95% CI)** | **P** | **β (95% CI)** | **P** |
| **FEV1 z-score** |  |  |  |  |
| Non-sarcopenia | Ref. | — | Ref. | — |
| Probable sarcopenia | -0.39 (-0.41, -0.38) | <0.001 | -0.28 (-0.30, -0.27) | <0.001 |
| Confirmed/Severe sarcopenia | -0.65 (-0.72, -0.58) | <0.001 | -0.55 (-0.62, -0.49) | <0.001 |
| **FVC z-score** |  |  |  |  |
| Non-sarcopenia | Ref. | — | Ref. | — |
| Probable sarcopenia | -0.38 (-0.39, -0.36) | <0.001 | -0.27 (-0.28, -0.25) | <0.001 |
| Confirmed/Severe sarcopenia | -0.50 (-0.57, -0.43) | <0.001 | -0.49 (-0.55, -0.42) | <0.001 |
| **PEF z-score** |  |  |  |  |
| Non-sarcopenia | Ref. | — | Ref. | — |
| Probable sarcopenia | -0.40 (-0.41, -0.39) | <0.001 | -0.31 (-0.33, -0.30) | <0.001 |
| Confirmed/Severe sarcopenia | -0.77 (-0.85, -0.70) | <0.001 | -0.59 (-0.66, -0.52) | <0.001 |
| **FEV1/FVC ratio** |  |  |  |  |
| Non-sarcopenia | Ref. | — | Ref. | — |
| Probable sarcopenia | -0.36 (-0.47, -0.24) | <0.001 | -0.36 (-0.47, -0.25) | <0.001 |
| Confirmed/Severe sarcopenia | -2.80 (-3.40, -2.20) | <0.001 | -1.20 (-1.79, -0.61) | <0.001 |
| **Lung function score** |  |  |  |  |
| Non-sarcopenia | Ref. | — | Ref. | — |
| Probable sarcopenia | -0.39 (-0.40, -0.38) | <0.001 | -0.29 (-0.30, -0.28) | <0.001 |
| Confirmed/Severe sarcopenia | -0.64 (-0.70, -0.58) | <0.001 | -0.54 (-0.60, -0.48) | <0.001 |

CI, confidence interval; FEV1, forced expiratory volume in one second; FVC, forced vital capacity; PEF, peak expiratory flow.

Model 1 was adjusted for age, and sex.

Model 2 was further adjusted for ethnicity, educational attainment, occupational status, Townsend deprivation index, smoking status, alcohol consumption, healthy diet, regular exercise, sleep duration, body mass index, and waist circumference based on model 1.

# **Supplementary Table S5. Cumulative incidences and risk differences at 5- and 10- year of respiratory disease among three categories of sarcopenia.**

|  | **Cumulative incidence (%) (95% CI)** | | **Risk difference (%) (95% CI)** | |
| --- | --- | --- | --- | --- |
|  | **5-year** | **10-year** | **5-year** | **10-year** |
| **Respiratory disease** |  |  |  |  |
| Non-sarcopenia | 3.29 (3.23, 3.36) | 9.00 (8.90, 9.10) | Ref. | Ref. |
| Probable sarcopenia | 5.63 (5.23, 6.02) | 14.96 (14.34, 15.58) | 2.33 (1.92, 2.75) | 5.96 (5.28, 6.65) |
| Confirmed/severe sarcopenia | 6.17 (3.85, 8.44) | 20.76 (16.76, 24.58) | 2.88 (0.49, 5.27) | 11.76 (5.28, 16.19) |
| **COPD** |  |  |  |  |
| Non-sarcopenia | 0.29 (0.27, 0.31) | 1.14 (1.11, 1.18) | Ref. | Ref. |
| Probable sarcopenia | 0.72 (0.58, 0.87) | 2.41 (2.14, 2.68) | 0.43 (0.28, 0.58) | 1.27 (0.99, 1.54) |
| Confirmed/severe sarcopenia | 0.72 (0.00, 1.53) | 6.99 (4.46, 9.46) | 0.43 (-0.39, 1.25) | 5.85 (0.99, 8.47) |
| **Asthma** |  |  |  |  |
| Non-sarcopenia | 0.55 (0.52, 0.58) | 1.55 (1.51, 1.60) | Ref. | Ref. |
| Probable sarcopenia | 0.98 (0.81, 1.15) | 2.77 (2.49, 3.06) | 0.43 (0.26, 0.60) | 1.22 (0.92, 1.52) |
| Confirmed/severe sarcopenia | 0.23 (0.00, 0.69) | 1.24 (0.15, 2.32) | -0.32 (-0.78, 0.15) | -0.31 (0.92, 0.81) |
| **Interstitial lung disease** | |  |  |  |
| Non-sarcopenia | 0.07 (0.06, 0.08) | 0.26 (0.24, 0.28) | Ref. | Ref. |
| Probable sarcopenia | 0.27 (0.18, 0.36) | 0.83 (0.67, 0.99) | 0.20 (0.11, 0.30) | 0.57 (0.41, 0.73) |
| Confirmed/severe sarcopenia | 0.23 (0.00, 0.69) | 1.48 (0.30, 2.65) | 0.17 (-0.30, 0.63) | 1.22 (0.41, 2.44) |

CI, confidence interval; COPD, chronic obstructive pulmonary disease.

The models were unadjusted.

# **Supplementary Table S6. Associations of probable and confirmed/severe sarcopenia with the risk of respiratory disease (N=317,628).**

|  | **No of events/No of participants** | **Events/1,000 person-years** | **Model 1** | | **Model 2** | |
| --- | --- | --- | --- | --- | --- | --- |
|  |  |  | **HR (95% CI)** | **P** | **HR (95% CI)** | **P** |
| **Respiratory disease** |  |  |  |  |  |  |
| Non-sarcopenia | 43,228/304,232 | 11.11 | Ref. | — | Ref. | — |
| Probable sarcopenia | 2,847/12,969 | 18.20 | 1.45 (1.40, 1.51) | <0.001 | 1.29 (1.24, 1.34) | <0.001 |
| Confirmed/Severe sarcopenia | 124/427 | 25.41 | 1.74 (1.46, 2.08) | <0.001 | 1.81 (1.51, 2.16) | <0.001 |
| **COPD** |  |  |  |  |  |  |
| Non-sarcopenia | 5,764/304,232 | 1.42 | Ref. | — | Ref. | — |
| Probable sarcopenia | 470/12,969 | 2.80 | 1.62 (1.47, 1.78) | <0.001 | 1.32 (1.20, 1.46) | <0.001 |
| Confirmed/Severe sarcopenia | 38/427 | 7.24 | 3.35 (2.42, 4.63) | <0.001 | 2.28 (1.61, 3.22) | <0.001 |
| **Asthma** |  |  |  |  |  |  |
| Non-sarcopenia | 7,086/304,232 | 1.75 | Ref. | — | Ref. | — |
| Probable sarcopenia | 492/12,969 | 2.95 | 1.56 (1.43, 1.71) | <0.001 | 1.36 (1.24, 1.49) | <0.001 |
| Confirmed/Severe sarcopenia | 8/427 | 1.50 | 0.82 (0.41, 1.65) | 0.584 | 1.03 (0.52, 2.07) | 0.924 |
| **Interstitial lung disease** |  |  |  |  |  |  |
| Non-sarcopenia | 1,637/304,232 | 0.40 | Ref. | — | Ref. | — |
| Probable sarcopenia | 165/12,969 | 0.98 | 1.94 (1.65, 2.29) | <0.001 | 1.71 (1.45, 2.01) | <0.001 |
| Confirmed/Severe sarcopenia | 9/427 | 1.68 | 2.38 (1.23, 4.62) | 0.010 | 2.68 (1.36, 5.27) | 0.004 |

HR, hazard ratio; CI, confidence interval; COPD, chronic obstructive pulmonary disease.

Model 1 was adjusted for age, and sex.

Model 2 was further adjusted for ethnicity, educational attainment, occupational status, Townsend deprivation index, smoking status, alcohol consumption, healthy diet, regular exercise, sleep duration, body mass index, and waist circumference based on model 1.

# **Supplementary Table S7. Cross-sectional associations of sarcopenia component with lung function at baseline.**

|  | **Model 1** | | **Model 2** | |
| --- | --- | --- | --- | --- |
|  | **β (95% CI)** | **P** | **β (95% CI)** | **P** |
| **FEV1 z-score** | | | | |
| **HGS (per SD increment)** | 0.30 (0.30, 0.30) | <0.001 | 0.26 (0.26, 0.26) | <0.001 |
| **MMI (per SD increment)** | -0.09 (-0.10, -0.09) | <0.001 | 0.11 (0.10, 0.12) | <0.001 |
| **Low MMI** |  |  |  |  |
| No | Ref. | — | Ref. | — |
| Yes | -0.13 (-0.15, -0.11) | <0.001 | -0.20 (-0.22, -0.18) | <0.001 |
| **Low physical performance** |  |  |  |  |
| No | Ref. | — | Ref. | — |
| Yes | -0.37 (-0.38, -0.36) | <0.001 | -0.16 (-0.17, -0.14) | <0.001 |
| **FVC z-score** | | | | |
| **HGS (per SD increment)** | 0.29 (0.29, 0.30) | <0.001 | 0.26 (0.25, 0.26) | <0.001 |
| **MMI (per SD increment)** | -0.15 (-0.15, -0.14) | <0.001 | 0.10 (0.09, 0.11) | <0.001 |
| **Low MMI** |  |  |  |  |
| No | Ref. | — | Ref. | — |
| Yes | 0.02 (-0.01, 0.04) | 0.145 | -0.12 (-0.15, -0.10) | <0.001 |
| **Low physical performance** |  |  |  |  |
| No | Ref. | — | Ref. | — |
| Yes | -0.36 (-0.37, -0.35) | <0.001 | -0.13 (-0.14, -0.12) | <0.001 |
| **PEF z-score** | | | | |
| **HGS (per SD increment)** | 0.30 (0.29, 0.30) | <0.001 | 0.26 (0.26, 0.26) | <0.001 |
| **MMI (per SD increment)** | 0.01 (0.00, 0.01) | 0.002 | 0.09 (0.08, 0.10) | <0.001 |
| **Low MMI** |  |  |  |  |
| No | Ref. | — | Ref. | — |
| Yes | -0.31 (-0.33, -0.29) | <0.001 | -0.25 (-0.28, -0.23) | <0.001 |
| **Low physical performance** |  |  |  |  |
| No | Ref. | — | Ref. | — |
| Yes | -0.31 (-0.32, -0.29) | <0.001 | -0.18 (-0.19, -0.17) | <0.001 |
| **FEV1/FVC ratio (%)** | | | | |
| **HGS (per SD increment)** | 0.03 (-0.01, 0.06) | 0.096 | -0.06 (-0.09, -0.02) | 0.001 |
| **MMI (per SD increment)** | 0.99 (0.96, 1.02) | <0.001 | 0.00 (-0.07, 0.07) | 0.929 |
| **Low MMI** |  |  |  |  |
| No | Ref. | — | Ref. | — |
| Yes | -2.41 (-2.59, -2.23) | <0.001 | -0.96 (-1.14, -0.78) | <0.001 |
| **Low physical performance** |  |  |  |  |
| No | Ref. | — | Ref. | — |
| Yes | -0.17 (-0.27, -0.07) | 0.001 | -0.51 (-0.61, -0.40) | <0.001 |
| **Lung function score** | | | | |
| **HGS (per SD increment)** | 0.30 (0.29, 0.30) | <0.001 | 0.26 (0.26, 0.26) | <0.001 |
| **MMI (per SD increment)** | -0.08 (-0.08, -0.07) | <0.001 | 0.10 (0.09, 0.11) | <0.001 |
| **Low MMI** |  |  |  |  |
| No | Ref. | — | Ref. | — |
| Yes | -0.14 (-0.16, -0.12) | <0.001 | -0.19 (-0.21, -0.17) | <0.001 |
| **Low physical performance** |  |  |  |  |
| No | Ref. | — | Ref. | — |
| Yes | -0.34 (-0.35, -0.33) | <0.001 | -0.16 (-0.17, -0.15) | <0.001 |

CI, confidence interval; FEV1, forced expiratory volume in one second; HGS, handgrip strength; SD, standard deviation; MMI, muscle mass index; FVC, forced vital capacity; PEF, peak expiratory flow.

Model 1 was adjusted for age, and sex.

Model 2 was further adjusted for ethnicity, educational attainment, occupational status, Townsend deprivation index, smoking status, alcohol consumption, healthy diet, regular exercise, sleep duration, body mass index, and waist circumference.

# **Supplementary Table S8. Associations of sarcopenia component with the risk of respiratory disease.**

|  | **Model 1** | | **Model 2** | |
| --- | --- | --- | --- | --- |
|  | **HR (95% CI)** | **P** | **HR (95% CI)** | **P** |
| **Respiratory disease** | | | | |
| **HGS (per SD increment) (N=317,534)** | 0.83 (0.82, 0.85) | <0.001 | 0.87 (0.86, 0.88) | <0.001 |
| **MMI (per SD increment) (N=314,065)** | 1.23 (1.21, 1.25) | <0.001 | 1.01 (0.98, 1.04) | 0.738 |
| **Low MMI (N=314,065)** |  |  |  |  |
| No (44,469/309,269) | Ref. | — | Ref. | — |
| Yes (1,008/4,796) | 1.17 (1.10, 1.25) | <0.001 | 1.40 (1.32, 1.50) | <0.001 |
| **Low physical performance (N=317,016)** |  |  |  |  |
| No (41,473/300,217) | Ref. | — | Ref. | — |
| Yes (4603/16,799) | 2.06 (2.00, 2.12) | <0.001 | 1.51 (1.46, 1.56) | <0.001 |
| **COPD** | | | | |
| **HGS (per SD increment) (N=317,534)** | 0.74 (0.71, 0.77) | <0.001 | 0.82 (0.79, 0.85) | <0.001 |
| **MMI (per SD increment) (N=314,065)** | 1.11 (1.07, 1.16) | <0.001 | 0.92 (0.85, 1.00) | 0.059 |
| **Low MMI (N=314,065)** |  |  |  |  |
| No (5,888/309,269) | Ref. | — | Ref. | — |
| Yes (287/4,796) | 2.21 (1.96, 2.50) | <0.001 | 2.05 (1.80, 2.34) | <0.001 |
| **Low physical performance (N=317,016)** |  |  |  |  |
| No (5,251/300,217) | Ref. | — | Ref. | — |
| Yes (1,000/16,799) | 3.21 (2.99, 3.43) | <0.001 | 1.78 (1.65,1.92) | <0.001 |
| **Asthma** | | | | |
| **HGS (per SD increment) (N=317,534)** | 0.81 (0.78, 0.84) | <0.001 | 0.85 (0.82, 0.88) | <0.001 |
| **MMI (per SD increment) (N=314,065)** | 1.39 (1.35, 1.43) | <0.001 | 0.95 (0.89, 1.02) | 0.168 |
| **Low MMI (N=314,065)** |  |  |  |  |
| No (7,399/309,269) | Ref. | — | Ref. | — |
| Yes (92/4,796) | 0.80 (0.65, 0.98) | 0.030 | 1.15 (0.93, 1.42) | 0.190 |
| **Low physical performance (N=317,016)** |  |  |  |  |
| No (6,788/300,217) | Ref. | — | Ref. | — |
| Yes (779/16,799) | 2.06 (1.92, 2.22) | <0.001 | 1.41 (1.30, 1.53) | <0.001 |
| **Interstitial lung disease** | | | | |
| **HGS (per SD increment) (N=317,534)** | 0.73 (0.68, 0.79) | <0.001 | 0.77 (0.71, 0.83) | <0.001 |
| **MMI (per SD increment) (N=314,065)** | 1.26 (1.18, 1.34) | <0.001 | 0.78 (0.67, 0.91) | 0.002 |
| **Low MMI (N=314,065)** |  |  |  |  |
| No (1,735/309,269) | Ref. | — | Ref. | — |
| Yes (46/4,796) | 1.01 (0.75, 1.36) | 0.951 | 1.34 (0.99, 1.82) | 0.058 |
| **Low physical performance (N=317,016)** |  |  |  |  |
| No (1,580/300,217) | Ref. | — | Ref. | — |
| Yes (228/16,799) | 2.32 (2.02, 2.67) | <0.001 | 1.57 (1.35, 1.83) | <0.001 |

CI, confidence interval; HGS, handgrip strength; SD, standard deviation; MMI, muscle mass index; COPD, chronic obstructive pulmonary disease.

Model 1 was adjusted for age, and sex.

Model 2 was further adjusted for ethnicity, educational attainment, occupational status, Townsend deprivation index, smoking status, alcohol consumption, healthy diet, regular exercise, sleep duration, body mass index, and waist circumference.

# **Supplementary Table S9. Cross-sectional associations of probable sarcopenia with lung function level at baseline stratified by age categories.**

|  |  | **β (95% CI)** | **P** | **Interactive P value** |
| --- | --- | --- | --- | --- |
| **FEV1 z-score** |  |  |  | <0.001 |
| <60 years | Non-sarcopenia | Ref. | — |  |
|  | Probable sarcopenia | -0.36 (-0.38, -0.34) | <0.001 |  |
| ≥60years | Non-sarcopenia | Ref. | — |  |
|  | Probable sarcopenia | -0.25 (-0.26, -0.23) | <0.001 |  |
| **FVC z-score** |  |  |  | <0.001 |
| <60 years | Non-sarcopenia | Ref. | — |  |
|  | Probable sarcopenia | -0.33 (-0.35, -0.31) | <0.001 |  |
| ≥60years | Non-sarcopenia | Ref. | — |  |
|  | Probable sarcopenia | -0.23 (-0.25, -0.21) | <0.001 |  |
| **PEF z-score** |  |  |  | <0.001 |
| <60 years | Non-sarcopenia | Ref. | — |  |
|  | Probable sarcopenia | -0.38 (-0.41, -0.36) | <0.001 |  |
| ≥60years | Non-sarcopenia | Ref. | — |  |
|  | Probable sarcopenia | -0.27 (-0.29, 0.26) | <0.001 |  |
| **FEV1/FVC ratio (%)** |  |  |  | 0.623 |
| <60 years | Non-sarcopenia | Ref. | — |  |
|  | Probable sarcopenia | -0.44 (-0.60, -0.28) | <0.001 |  |
| ≥60years | Non-sarcopenia | Ref. | — |  |
|  | Probable sarcopenia | -0.31 (-0.47, -0.16) | <0.001 |  |
| **Lung function score** |  |  |  | <0.001 |
| <60 years | Non-sarcopenia | Ref. | — |  |
|  | Probable sarcopenia | -0.36 (-0.37, -0.34) | <0.001 |  |
| ≥60years | Non-sarcopenia | Ref. | — |  |
|  | Probable sarcopenia | -0.25 (-0.26, -0.24) | <0.001 |  |

CI, confidence interval; FEV1, forced expiratory volume in one second; FVC, forced vital capacity; PEF, peak expiratory flow.

The models were adjusted for age, sex, ethnicity, educational attainment, occupational status, Townsend deprivation index, smoking status, alcohol consumption, healthy diet, regular exercise, sleep duration, body mass index, and waist circumference.

# **Supplementary Table S10. Cross-sectional associations of probable sarcopenia with lung function at baseline stratified by sex.**

|  |  | **β (95% CI)** | **P** | **Interactive P value** |
| --- | --- | --- | --- | --- |
| **FEV1 z-score** |  |  |  | <0.001 |
| Female | Non-sarcopenia | Ref. | — |  |
|  | Probable sarcopenia | -0.22 (-0.23, -0.21) | <0.001 |  |
| Male | Non-sarcopenia | Ref. | — |  |
|  | Probable sarcopenia | -0.42 (-0.45, -0.40) | <0.001 |  |
| **FVC z-score** |  |  |  | <0.001 |
| Female | Non-sarcopenia | Ref. | — |  |
|  | Probable sarcopenia | -0.20 (-0.22, -0.19) | <0.001 |  |
| Male | Non-sarcopenia | Ref. | — |  |
|  | Probable sarcopenia | -0.40 (-0.42, -0.37) | <0.001 |  |
| **PEF z-score** |  |  |  | <0.001 |
| Female | Non-sarcopenia | Ref. | — |  |
|  | Probable sarcopenia | -0.23 (-0.24, -0.22) | <0.001 |  |
| Male | Non-sarcopenia | Ref. | — |  |
|  | Probable sarcopenia | -0.48 (-0.50, -0.45) | <0.001 |  |
| **FEV1/FVC ratio (%)** |  |  |  | 0.820 |
| Female | Non-sarcopenia | Ref. | — |  |
|  | Probable sarcopenia | -0.35 (-0.48, -0.21) | <0.001 |  |
| Male | Non-sarcopenia | Ref. | — |  |
|  | Probable sarcopenia | -0.44 (-0.62, -0.26) | <0.001 |  |
| **Lung function score** |  |  |  | <0.001 |
| Female | Non-sarcopenia | Ref. | — |  |
|  | Probable sarcopenia | -0.22 (-0.23, -0.21) | <0.001 |  |
| Male | Non-sarcopenia | Ref. | — |  |
|  | Probable sarcopenia | -0.43 (-0.45, -0.41) | <0.001 |  |

CI, confidence interval; FEV1, forced expiratory volume in one second; FVC, forced vital capacity; PEF, peak expiratory flow.

The models were adjusted for age, ethnicity, educational attainment, occupational status, Townsend deprivation index, smoking status, alcohol consumption, healthy diet, regular exercise, sleep duration, body mass index, and waist circumference.

# **Supplementary Table S11. Associations of probable sarcopenia with the risk of respiratory disease stratified by age categories.**

|  |  | **No of events/No of participants** | **HR (95% CI)** | **P** | **Interactive P value** |
| --- | --- | --- | --- | --- | --- |
| **Respiratory disease** |  |  |  |  | 0.012 |
| <60 years | Non-sarcopenia | 19,933/187,915 | Ref. | — |  |
|  | Probable sarcopenia | 976/5,645 | 1.40 (1.31, 1.49) | <0.001 |  |
| ≥60years | Non-sarcopenia | 23,295/116,317 | Ref. | — |  |
|  | Probable sarcopenia | 1,995/7,751 | 1.26 (1.20, 1.32) | <0.001 |  |
| **COPD** |  |  |  |  | 0.002 |
| <60 years | Non-sarcopenia | 2,010/187,915 | Ref. | — |  |
|  | Probable sarcopenia | 153/5,645 | 1.67 (1.40, 1.98) | <0.001 |  |
| ≥60years | Non-sarcopenia | 3,754/116,317 | Ref. | — |  |
|  | Probable sarcopenia | 355/7,751 | 1.27 (1.14, 1.42) | <0.001 |  |
| **Asthma** |  |  |  |  | 0.210 |
| <60 years | Non-sarcopenia | 3,951/187,915 | Ref. | — |  |
|  | Probable sarcopenia | 209/5,645 | 1.44 (1.25, 1.66) | <0.001 |  |
| ≥60years | Non-sarcopenia | 3,135/116,317 | Ref. | — |  |
|  | Probable sarcopenia | 291/7,751 | 1.28 (1.13, 1.44) | <0.001 |  |
| **Interstitial lung disease** |  |  |  |  | 0.868 |
| <60 years | Non-sarcopenia | 448/187,915 | Ref. | — |  |
|  | Probable sarcopenia | 31/5,645 | 1.81 (1.25, 2.62) | 0.002 |  |
| ≥60years | Non-sarcopenia | 1,189/116,317 | Ref. | — |  |
|  | Probable sarcopenia | 143/7,751 | 1.73 (1.44, 2.07) | <0.001 |  |

HR, hazard ratio; CI, confidence interval; COPD, chronic obstructive pulmonary disease.

The models were adjusted for age, sex, ethnicity, educational attainment, occupational status, Townsend deprivation index, smoking status, alcohol consumption, healthy diet, regular exercise, sleep duration, body mass index, and waist circumference.

# **Supplementary Table S12. Associations of probable sarcopenia with the risk of respiratory disease stratified by sex.**

|  |  | **No of events/No of participants** | **HR (95% CI)** | **P** | **Interactive P value** |
| --- | --- | --- | --- | --- | --- |
| **Respiratory disease** |  |  |  |  | 0.876 |
| Female | Non-sarcopenia | 20,611/166,602 | Ref. | — |  |
|  | Probable sarcopenia | 1,699/8,357 | 1.32 (1.26, 1.39) | <0.001 |  |
| Male | Non-sarcopenia | 22,617/137,630 | Ref. | — |  |
|  | Probable sarcopenia | 1,272/5,039 | 1.30 (1.22, 1.37) | <0.001 |  |
| **COPD** |  |  |  |  | 0.327 |
| Female | Non-sarcopenia | 2,516/166,602 | Ref. | — |  |
|  | Probable sarcopenia | 280/8,357 | 1.44 (1.27, 1.63) | <0.001 |  |
| Male | Non-sarcopenia | 3,248/137,630 | Ref. | — |  |
|  | Probable sarcopenia | 228/5,039 | 1.29 (1.12, 1.48) | <0.001 |  |
| **Asthma** |  |  |  |  | 0.136 |
| Female | Non-sarcopenia | 4,139/166,602 | Ref. | — |  |
|  | Probable sarcopenia | 351/8,357 | 1.44 (1.29, 1.61) | <0.001 |  |
| Male | Non-sarcopenia | 2,947/137,630 | Ref. | — |  |
|  | Probable sarcopenia | 149/5,039 | 1.18 (1.00, 1.40) | 0.050 |  |
| **Interstitial lung disease** |  |  |  |  | 0.506 |
| Female | Non-sarcopenia | 611/166,602 | Ref. | — |  |
|  | Probable sarcopenia | 87/8,357 | 1.92 (1.52, 2.41) | <0.001 |  |
| Male | Non-sarcopenia | 1,026/137,630 | Ref. | — |  |
|  | Probable sarcopenia | 87/5,039 | 1.64 (1.31, 2.06) | <0.001 |  |

HR, hazard ratio; CI, confidence interval; COPD, chronic obstructive pulmonary disease.

The models were adjusted for age, ethnicity, educational attainment, occupational status, Townsend deprivation index, smoking status, alcohol consumption, healthy diet, regular exercise, sleep duration, body mass index, and waist circumference.

# **Supplementary Table S13. Associations of probable sarcopenia with the risk of respiratory disease after further adjusting for lung function at baseline.**

|  | **No of events/No of participants** | **HR (95% CI)** | **P** |
| --- | --- | --- | --- |
| **Respiratory disease** |  |  |  |
| Non-sarcopenia | 43,228/304,232 | Ref. | — |
| Probable sarcopenia | 2,971/13,396 | 1.23 (1.18, 1.28) | <0.001 |
| **COPD** |  |  |  |
| Non-sarcopenia | 5,764/304,232 | Ref. | — |
| Probable sarcopenia | 508/13,396 | 1.09 (0.99, 1.21) | 0.071 |
| **Asthma** |  |  |  |
| Non-sarcopenia | 7,086/304,232 | Ref. | — |
| Probable sarcopenia | 500/13,396 | 1.24 (1.13, 1.36) | <0.001 |
| **Interstitial lung disease** |  |  |  |
| Non-sarcopenia | 1,637/304,232 | Ref. | — |
| Probable sarcopenia | 174/13,396 | 1.63 (1.38, 1.92) | <0.001 |

HR, hazard ratio; CI, confidence interval; COPD, chronic obstructive pulmonary disease.

The models were adjusted for age, sex, ethnicity, educational attainment, occupational status, Townsend deprivation index, smoking status, alcohol consumption, healthy diet, regular exercise, sleep duration, body mass index, waist circumference, forced expiratory volume in one second, forced vital capacity, and peak expiratory flow.

# **Supplementary Table S14. Associations of probable sarcopenia with risk of respiratory disease after excluding those who were followed up for less than 2 years.**

|  | **No of events/No of participants** | **HR (95% CI)** | **P** |
| --- | --- | --- | --- |
| **Respiratory disease** |  |  |  |
| Non-sarcopenia | 40,088/300,538 | Ref. | — |
| Probable sarcopenia | 2,735/13,114 | 1.30 (1.25, 1.35) | <0.001 |
| **COPD** |  |  |  |
| Non-sarcopenia | 5,581/303,279 | Ref. | — |
| Probable sarcopenia | 488/13,311 | 1.37 (1.24, 1.50) | <0.001 |
| **Asthma** |  |  |  |
| Non-sarcopenia | 6,654/303,027 | Ref. | — |
| Probable sarcopenia | 462/13,292 | 1.33 (1.21, 1.46) | <0.001 |
| **Interstitial lung disease** |  |  |  |
| Non-sarcopenia | 1,577/303,404 | Ref. | — |
| Probable sarcopenia | 166/13,322 | 1.73 (1.46, 2.03) | <0.001 |

HR, hazard ratio; CI, confidence interval; COPD, chronic obstructive pulmonary disease.

The models were adjusted for age, sex, ethnicity, educational attainment, occupational status, Townsend deprivation index, smoking status, alcohol consumption, healthy diet, regular exercise, sleep duration, body mass index, and waist circumference.

# **Supplementary Table S15. Associations of probable sarcopenia with blood inflammatory markers.**

|  | **Non-sarcopenia** | **Probable sarcopenia** | |
| --- | --- | --- | --- |
|  | **β (95% CI)** | **β (95% CI)** | **FDR** |
| Leukocyte count | Ref. | 0.014 (0.010, 0.019) | <0.001 |
| Neutrophil count | Ref. | 0.020 (0.014, 0.025) | <0.001 |
| Neutrophil percentage | Ref. | 0.005 (0.003, 0.008) | <0.001 |
| Monocyte count | Ref. | 0.016 (0.009, 0.022) | <0.001 |
| Monocyte percentage | Ref. | 0.001 (-0.005, 0.007) | 0.765 |
| Lymphocyte count | Ref. | -0.004 (-0.010, 0.001) | 0.113 |
| Lymphocyte percentage | Ref. | -0.019 (-0.023, -0.014) | <0.001 |
| C-reactive protein | Ref. | 0.180 (0.164, 0.197) | <0.001 |
| Platelet count | Ref. | 0.012 (0.008, 0.017) | <0.001 |
| SII | Ref. | 0.036 (0.028, 0.045) | <0.001 |
| NLR | Ref. | 0.024 (0.017, 0.031) | <0.001 |
| PLR | Ref. | 0.017 (0.010, 0.023) | <0.001 |
| LMR | Ref. | -0.020 (-0.027, -0.013) | <0.001 |

CI, confidence interval; FDR, false discovery rate; SII, systemic immune-inflammation index; NLR, neutrophil-to-lymphocyte ratio; PLR, platelet-to-lymphocyte ratio; LMR, lymphocyte-to-monocyte ratio.

The models were adjusted for age, sex, ethnicity, educational attainment, occupational status, Townsend deprivation index, smoking status, alcohol consumption, healthy diet, regular exercise, sleep duration, body mass index, and waist circumference.

# **Supplementary Table S16. Associations of probable sarcopenia with metabolites (N=166,721).**

|  | **Non-sarcopenia** | **Probable sarcopenia** | |
| --- | --- | --- | --- |
|  | **β (95% CI)** | **β (95% CI)** | **FDR** |
| Total cholesterol | Ref. | -0.074 (-0.097, -0.051) | <0.001 |
| Total cholesterol minus HDL-C | Ref. | -0.066 (-0.090, -0.042) | <0.001 |
| Remnant cholesterol (non-HDL, non-LDL -cholesterol) | Ref. | -0.056 (-0.079, -0.032) | <0.001 |
| VLDL cholesterol | Ref. | -0.022 (-0.046, 0.002) | 0.088 |
| Clinical LDL cholesterol | Ref. | -0.077 (-0.101, -0.054) | <0.001 |
| LDL cholesterol | Ref. | -0.072 (-0.096, -0.048) | <0.001 |
| HDL cholesterol | Ref. | -0.049 (-0.069, -0.029) | <0.001 |
| Total triglycerides | Ref. | 0.033 (0.011, 0.056) | 0.006 |
| Triglycerides in VLDL | Ref. | 0.029 (0.007, 0.052) | 0.015 |
| Triglycerides in LDL | Ref. | 0.050 (0.026, 0.073) | <0.001 |
| Triglycerides in HDL | Ref. | 0.036 (0.013, 0.060) | 0.004 |
| Total phospholipids in lipoprotein particles | Ref. | -0.044 (-0.066, -0.021) | <0.001 |
| Phospholipids in VLDL | Ref. | 0.005 (-0.018, 0.029) | 0.686 |
| Phospholipids in LDL | Ref. | -0.065 (-0.089, -0.041) | <0.001 |
| Phospholipids in HDL | Ref. | -0.026 (-0.047, -0.005) | 0.020 |
| Total esterified cholesterol | Ref. | -0.076 (-0.099, -0.053) | <0.001 |
| Cholesteryl esters in VLDL | Ref. | -0.031 (-0.055, -0.007) | 0.016 |
| Cholesteryl esters in LDL | Ref. | -0.066 (-0.090, -0.042) | <0.001 |
| Cholesteryl esters in HDL | Ref. | -0.051 (-0.071, -0.031) | <0.001 |
| Total free cholesterol | Ref. | -0.066 (-0.089, -0.043) | <0.001 |
| Free cholesterol in VLDL | Ref. | -0.006 (-0.029, 0.018) | 0.676 |
| Free cholesterol in LDL | Ref. | -0.084 (-0.108, -0.061) | <0.001 |
| Free cholesterol in HDL | Ref. | -0.04 (-0.061, -0.020) | <0.001 |
| Total lipids in lipoprotein particles | Ref. | -0.044 (-0.068, -0.021) | <0.001 |
| Total lipids in VLDL | Ref. | 0.008 (-0.015, 0.031) | 0.543 |
| Total lipids in LDL | Ref. | -0.066 (-0.089, -0.042) | <0.001 |
| Total lipids in HDL | Ref. | -0.035 (-0.055, -0.014) | 0.002 |
| Total concentration of lipoprotein particles | Ref. | -0.053 (-0.075, -0.031) | <0.001 |
| Concentration of VLDL particles | Ref. | -0.002 (-0.025, 0.022) | 0.913 |
| Concentration of LDL particles | Ref. | -0.049 (-0.073, -0.024) | <0.001 |
| Concentration of HDL particles | Ref. | -0.048 (-0.07, -0.026) | <0.001 |
| Average diameter for VLDL particles | Ref. | 0.017 (-0.004, 0.038) | 0.149 |
| Average diameter for LDL particles | Ref. | -0.019 (-0.043, 0.004) | 0.129 |
| Average diameter for HDL particles | Ref. | -0.022 (-0.043, -0.002) | 0.039 |
| Phosphoglycerides | Ref. | -0.031 (-0.053, -0.008) | 0.011 |
| Total cholines | Ref. | -0.040 (-0.063, -0.018) | 0.001 |
| Phosphatidylcholines | Ref. | -0.036 (-0.058, -0.014) | 0.002 |
| Sphingomyelins | Ref. | -0.054 (-0.077, -0.032) | <0.001 |
| Apolipoprotein B | Ref. | -0.050 (-0.074, -0.026) | <0.001 |
| Apolipoprotein A1 | Ref. | -0.038 (-0.059, -0.017) | 0.001 |
| Total fatty acids | Ref. | 0.003 (-0.021, 0.026) | 0.847 |
| Degree of unsaturation | Ref. | -0.091 (-0.113, -0.069) | <0.001 |
| Omega-3 fatty acids | Ref. | -0.049 (-0.073, -0.025) | <0.001 |
| Omega-6 fatty acids | Ref. | -0.038 (-0.061, -0.014) | 0.003 |
| Polyunsaturated fatty acids | Ref. | -0.045 (-0.069, -0.022) | <0.001 |
| Monounsaturated fatty acids | Ref. | 0.042 (0.019, 0.065) | 0.001 |
| Saturated fatty acids | Ref. | 0.012 (-0.011, 0.035) | 0.353 |
| Linoleic acid | Ref. | -0.043 (-0.067, -0.019) | 0.001 |
| Docosahexaenoic acid | Ref. | -0.069 (-0.092, -0.046) | <0.001 |
| Alanine | Ref. | -0.026 (-0.051, -0.002) | 0.047 |
| Glutamine | Ref. | -0.065 (-0.090, -0.041) | <0.001 |
| Glycine | Ref. | -0.014 (-0.037, 0.009) | 0.277 |
| Histidine | Ref. | -0.039 (-0.064, -0.015) | 0.003 |
| Total concentration of branched-chain amino acids (leucine + isoleucine + valine) | Ref. | -0.002 (-0.025, 0.022) | 0.911 |
| Isoleucine | Ref. | 0.026 (0.002, 0.050) | 0.044 |
| Leucine | Ref. | 0.000 (-0.023, 0.023) | 0.995 |
| Valine | Ref. | -0.012 (-0.036, 0.011) | 0.331 |
| Phenylalanine | Ref. | 0.068 (0.044, 0.092) | <0.001 |
| Tyrosine | Ref. | -0.014 (-0.038, 0.010) | 0.302 |
| Glucose | Ref. | 0.020 (-0.003, 0.044) | 0.113 |
| Lactate | Ref. | -0.017 (-0.041, 0.008) | 0.222 |
| Pyruvate | Ref. | 0.024 (-0.001, 0.049) | 0.077 |
| Citrate | Ref. | -0.115 (-0.139, -0.091) | <0.001 |
| 3-Hydroxybutyrate | Ref. | -0.005 (-0.030, 0.020) | 0.724 |
| Acetate | Ref. | 0.000 (-0.024, 0.024) | 0.995 |
| Acetoacetate | Ref. | 0.027 (0.003, 0.052) | 0.039 |
| Acetone | Ref. | 0.013 (-0.011, 0.038) | 0.325 |
| Creatinine | Ref. | -0.068 (-0.087, -0.048) | <0.001 |
| Albumin | Ref. | -0.148 (-0.172, -0.124) | <0.001 |
| Glycoprotein acetyls | Ref. | 0.102 (0.080, 0.124) | <0.001 |
| Concentration of chylomicrons and extremely large VLDL particles | Ref. | 0.049 (0.027, 0.071) | <0.001 |
| Total lipids in chylomicrons and extremely large VLDL | Ref. | 0.045 (0.023, 0.068) | <0.001 |
| Phospholipids in chylomicrons and extremely large VLDL | Ref. | 0.048 (0.026, 0.071) | <0.001 |
| Cholesterol in chylomicrons and extremely large VLDL | Ref. | 0.041 (0.019, 0.064) | 0.001 |
| Cholesteryl esters in chylomicrons and extremely large VLDL | Ref. | 0.038 (0.016, 0.061) | 0.002 |
| Free cholesterol in chylomicrons and extremely large VLDL | Ref. | 0.045 (0.022, 0.068) | <0.001 |
| Triglycerides in chylomicrons and extremely large VLDL | Ref. | 0.047 (0.025, 0.069) | <0.001 |
| Concentration of very large VLDL particles | Ref. | 0.035 (0.013, 0.057) | 0.003 |
| Total lipids in very large VLDL | Ref. | 0.032 (0.010, 0.054) | 0.008 |
| Phospholipids in very large VLDL | Ref. | 0.031 (0.009, 0.054) | 0.010 |
| Cholesterol in very large VLDL | Ref. | 0.013 (-0.009, 0.036) | 0.291 |
| Cholesteryl esters in very large VLDL | Ref. | 0.001 (-0.023, 0.024) | 0.969 |
| Free cholesterol in very large VLDL | Ref. | 0.027 (0.004, 0.049) | 0.029 |
| Triglycerides in very large VLDL | Ref. | 0.040 (0.018, 0.062) | 0.001 |
| Concentration of large VLDL particles | Ref. | 0.025 (0.003, 0.048) | 0.040 |
| Total lipids in large VLDL | Ref. | 0.017 (-0.005, 0.040) | 0.164 |
| Phospholipids in large VLDL | Ref. | 0.027 (0.005, 0.050) | 0.024 |
| Cholesterol in large VLDL | Ref. | 0.008 (-0.015, 0.031) | 0.537 |
| Cholesteryl esters in large VLDL | Ref. | -0.003 (-0.027, 0.020) | 0.808 |
| Free cholesterol in large VLDL | Ref. | 0.020 (-0.003, 0.042) | 0.109 |
| Triglycerides in large VLDL | Ref. | 0.020 (-0.002, 0.043) | 0.096 |
| Concentration of medium VLDL particles | Ref. | -0.027 (-0.051, -0.003) | 0.037 |
| Total lipids in medium VLDL | Ref. | -0.021 (-0.045, 0.003) | 0.103 |
| Phospholipids in medium VLDL | Ref. | -0.030 (-0.054, -0.006) | 0.021 |
| Cholesterol in medium VLDL | Ref. | -0.060 (-0.084, -0.036) | <0.001 |
| Cholesteryl esters in medium VLDL | Ref. | -0.071 (-0.095, -0.047) | <0.001 |
| Free cholesterol in medium VLDL | Ref. | -0.040 (-0.064, -0.016) | 0.002 |
| Triglycerides in medium VLDL | Ref. | 0.012 (-0.010, 0.035) | 0.325 |
| Concentration of small VLDL particles | Ref. | 0.009 (-0.014, 0.033) | 0.481 |
| Total lipids in small VLDL | Ref. | 0.002 (-0.022, 0.025) | 0.911 |
| Phospholipids in small VLDL | Ref. | -0.020 (-0.044, 0.004) | 0.135 |
| Cholesterol in small VLDL | Ref. | -0.024 (-0.048, 0.000) | 0.063 |
| Cholesteryl esters in small VLDL | Ref. | -0.015 (-0.039, 0.009) | 0.247 |
| Free cholesterol in small VLDL | Ref. | -0.038 (-0.062, -0.014) | 0.003 |
| Triglycerides in small VLDL | Ref. | 0.037 (0.014, 0.060) | 0.003 |
| Concentration of very small VLDL particles | Ref. | -0.011 (-0.035, 0.013) | 0.404 |
| Total lipids in very small VLDL | Ref. | -0.008 (-0.032, 0.015) | 0.537 |
| Phospholipids in very small VLDL | Ref. | 0.005 (-0.019, 0.029) | 0.703 |
| Cholesterol in very small VLDL | Ref. | -0.041 (-0.064, -0.017) | 0.001 |
| Cholesteryl esters in very small VLDL | Ref. | -0.050 (-0.073, -0.027) | <0.001 |
| Free cholesterol in very small VLDL | Ref. | -0.015 (-0.039, 0.008) | 0.246 |
| Triglycerides in very small VLDL | Ref. | 0.056 (0.033, 0.080) | <0.001 |
| Concentration of IDL particles | Ref. | -0.061 (-0.085, -0.037) | <0.001 |
| Total lipids in IDL | Ref. | -0.069 (-0.092, -0.047) | <0.001 |
| Phospholipids in IDL | Ref. | -0.062 (-0.085, -0.038) | <0.001 |
| Cholesterol in IDL | Ref. | -0.08 (-0.103, -0.058) | <0.001 |
| Cholesteryl esters in IDL | Ref. | -0.081 (-0.103, -0.058) | <0.001 |
| Free cholesterol in IDL | Ref. | -0.077 (-0.100, -0.054) | <0.001 |
| Triglycerides in IDL | Ref. | 0.054 (0.030, 0.077) | <0.001 |
| Concentration of large LDL particles | Ref. | -0.048 (-0.072, -0.024) | <0.001 |
| Total lipids in large LDL | Ref. | -0.071 (-0.095, -0.048) | <0.001 |
| Phospholipids in large LDL | Ref. | -0.073 (-0.097, -0.049) | <0.001 |
| Cholesterol in large LDL | Ref. | -0.077 (-0.101, -0.054) | <0.001 |
| Cholesteryl esters in large LDL | Ref. | -0.072 (-0.096, -0.049) | <0.001 |
| Free cholesterol in large LDL | Ref. | -0.087 (-0.111, -0.064) | <0.001 |
| Triglycerides in large LDL | Ref. | 0.050 (0.027, 0.074) | <0.001 |
| Concentration of medium LDL particles | Ref. | -0.048 (-0.073, -0.024) | <0.001 |
| Total lipids in medium LDL | Ref. | -0.052 (-0.076, -0.028) | <0.001 |
| Phospholipids in medium LDL | Ref. | -0.053 (-0.077, -0.028) | <0.001 |
| Cholesterol in medium LDL | Ref. | -0.057 (-0.081, -0.033) | <0.001 |
| Cholesteryl esters in medium LDL | Ref. | -0.048 (-0.072, -0.024) | <0.001 |
| Free cholesterol in medium LDL | Ref. | -0.077 (-0.101, -0.052) | <0.001 |
| Triglycerides in medium LDL | Ref. | 0.048 (0.025, 0.071) | <0.001 |
| Concentration of small LDL particles | Ref. | -0.041 (-0.065, -0.017) | 0.001 |
| Total lipids in small LDL | Ref. | -0.047 (-0.071, -0.023) | <0.001 |
| Phospholipids in small LDL | Ref. | -0.045 (-0.069, -0.021) | 0.001 |
| Cholesterol in small LDL | Ref. | -0.055 (-0.079, -0.031) | <0.001 |
| Cholesteryl esters in small LDL | Ref. | -0.046 (-0.070, -0.022) | <0.001 |
| Free cholesterol in small LDL | Ref. | -0.073 (-0.097, -0.048) | <0.001 |
| Triglycerides in small LDL | Ref. | 0.046 (0.023, 0.069) | <0.001 |
| Concentration of very large HDL particles | Ref. | -0.038 (-0.059, -0.017) | 0.001 |
| Total lipids in very large HDL | Ref. | -0.034 (-0.055, -0.013) | 0.002 |
| Phospholipids in very large HDL | Ref. | -0.029 (-0.050, -0.008) | 0.009 |
| Cholesterol in very large HDL | Ref. | -0.043 (-0.063, -0.022) | <0.001 |
| Cholesteryl esters in very large HDL | Ref. | -0.044 (-0.065, -0.024) | <0.001 |
| Free cholesterol in very large HDL | Ref. | -0.035 (-0.056, -0.013) | 0.003 |
| Triglycerides in very large HDL | Ref. | 0.025 (0.000, 0.049) | 0.062 |
| Concentration of large HDL particles | Ref. | -0.041 (-0.061, -0.021) | <0.001 |
| Total lipids in large HDL | Ref. | -0.035 (-0.055, -0.016) | 0.001 |
| Phospholipids in large HDL | Ref. | -0.030 (-0.050, -0.010) | 0.005 |
| Cholesterol in large HDL | Ref. | -0.043 (-0.062, -0.023) | <0.001 |
| Cholesteryl esters in large HDL | Ref. | -0.044 (-0.064, -0.025) | <0.001 |
| Free cholesterol in large HDL | Ref. | -0.037 (-0.057, -0.017) | 0.001 |
| Triglycerides in large HDL | Ref. | 0.008 (-0.015, 0.032) | 0.537 |
| Concentration of medium HDL particles | Ref. | -0.033 (-0.054, -0.011) | 0.004 |
| Total lipids in medium HDL | Ref. | -0.025 (-0.046, -0.003) | 0.032 |
| Phospholipids in medium HDL | Ref. | -0.016 (-0.037, 0.006) | 0.194 |
| Cholesterol in medium HDL | Ref. | -0.038 (-0.059, -0.017) | 0.001 |
| Cholesteryl esters in medium HDL | Ref. | -0.039 (-0.060, -0.018) | 0.001 |
| Free cholesterol in medium HDL | Ref. | -0.035 (-0.056, -0.014) | 0.002 |
| Triglycerides in medium HDL | Ref. | 0.037 (0.013, 0.061) | 0.004 |
| Concentration of small HDL particles | Ref. | -0.040 (-0.063, -0.016) | 0.002 |
| Total lipids in small HDL | Ref. | -0.018 (-0.042, 0.005) | 0.157 |
| Phospholipids in small HDL | Ref. | -0.011 (-0.034, 0.012) | 0.404 |
| Cholesterol in small HDL | Ref. | -0.043 (-0.067, -0.019) | 0.001 |
| Cholesteryl esters in small HDL | Ref. | -0.046 (-0.070, -0.022) | <0.001 |
| Free cholesterol in small HDL | Ref. | -0.029 (-0.052, -0.005) | 0.023 |
| Triglycerides in small HDL | Ref. | 0.050 (0.028, 0.072) | <0.001 |

CI, confidence interval; HDL-C, high-density lipoprotein cholesterol; HDL, high-density lipoprotein; LDL, low-density lipoprotein; VLDL, very low-density lipoprotein; IDL, Intermediate Density Lipoprotein.

The models were adjusted for age, sex, ethnicity, educational attainment, occupational status, Townsend deprivation index, smoking status, alcohol consumption, healthy diet, regular exercise, sleep duration, body mass index, and waist circumference.

# **Supplementary Table S17. Associations of inflammatory markers with the risk of respiratory disease.**

| **Inflammation markers** | **Respiratory disease** | | **COPD** | | **Asthma** | | **Interstitial lung disease** | |
| --- | --- | --- | --- | --- | --- | --- | --- | --- |
|  | **HR (95% CI)** | **FDR** | **HR (95% CI)** | **FDR** | **HR (95% CI)** | **FDR** | **HR (95% CI)** | **FDR** |
| Leukocyte count | 1.53 (1.47, 1.60) | <0.001 | 2.61 (2.35, 2.90) | <0.001 | 1.40 (1.27, 1.54) | <0.001 | 2.06 (1.69, 2.53) | <0.001 |
| Neutrophil count | 1.33 (1.29, 1.37) | <0.001 | 1.86 (1.71, 2.02) | <0.001 | 1.17 (1.08, 1.26) | <0.001 | 1.63 (1.39, 1.91) | <0.001 |
| Neutrophil percentage | 1.19 (1.11, 1.26) | <0.001 | 1.21 (1.02, 1.44) | 0.039 | 0.87 (0.76, 1.00) | 0.069 | 1.30 (0.94, 1.80) | 0.139 |
| Monocyte count | 1.20 (1.17, 1.24) | <0.001 | 1.49 (1.39, 1.60) | <0.001 | 1.13 (1.06, 1.21) | <0.001 | 1.54 (1.34, 1.77) | <0.001 |
| Monocyte percentage | 0.99 (0.96, 1.02) | 0.458 | 0.96 (0.89, 1.03) | 0.305 | 0.98 (0.91, 1.05) | 0.563 | 1.14 (0.99, 1.33) | 0.097 |
| Lymphocyte count | 1.02 (0.99, 1.05) | 0.251 | 1.38 (1.27, 1.50) | <0.001 | 1.05 (0.97, 1.14) | 0.210 | 1.02 (0.88, 1.19) | 0.787 |
| Lymphocyte percentage | 0.73 (0.70, 0.75) | <0.001 | 0.72 (0.65, 0.79) | <0.001 | 0.81 (0.75, 0.89) | <0.001 | 0.60 (0.51, 0.71) | <0.001 |
| C-reactive protein | 1.13 (1.12, 1.15) | <0.001 | 1.26 (1.23, 1.30) | <0.001 | 1.10 (1.07, 1.13) | <0.001 | 1.36 (1.30, 1.43) | <0.001 |
| Platelet count | 1.11 (1.07, 1.16) | <0.001 | 1.26 (1.13, 1.41) | <0.001 | 1.25 (1.13, 1.38) | <0.001 | 0.96 (0.78, 1.17) | 0.707 |
| SII | 1.16 (1.13, 1.18) | <0.001 | 1.20 (1.14, 1.27) | <0.001 | 1.11 (1.05, 1.16) | <0.001 | 1.20 (1.09, 1.33) | <0.001 |
| NLR | 1.18 (1.15, 1.21) | <0.001 | 1.20 (1.12, 1.28) | <0.001 | 1.07 (1.01, 1.14) | 0.030 | 1.32 (1.17, 1.48) | <0.001 |
| PLR | 1.03 (1.01, 1.06) | 0.021 | 0.87 (0.81, 0.94) | <0.001 | 1.06 (0.99, 1.13) | 0.094 | 0.97 (0.85, 1.10) | 0.671 |
| LMR | 0.87 (0.85, 0.89) | <0.001 | 0.88 (0.83, 0.94) | <0.001 | 0.93 (0.87, 0.98) | 0.016 | 0.72 (0.64, 0.81) | <0.001 |

COPD, chronic obstructive pulmonary disease; HR, hazard ratio; CI, confidence interval; FDR, false discovery rate; SII, systemic immune-inflammation index; NLR, neutrophil-to-lymphocyte ratio; PLR, platelet-to-lymphocyte ratio; LMR, lymphocyte-to-monocyte ratio.

The models were adjusted for age, sex, ethnicity, educational attainment, occupational status, Townsend deprivation index, smoking status, alcohol consumption, healthy diet, regular exercise, sleep duration, body mass index, and waist circumference.

# **Supplementary Table S18. Associations of metabolites with the risk of respiratory disease (N=166,721).**

| **Metabolites** | **Respiratory disease** | | **COPD** | | **Asthma** | | **Interstitial lung disease** | |
| --- | --- | --- | --- | --- | --- | --- | --- | --- |
|  | **HR (95% CI)** | **FDR** | **HR (95% CI)** | **FDR** | **HR (95% CI)** | **FDR** | **HR (95% CI)** | **FDR** |
| Total cholesterol | 0.92 (0.91, 0.94) | <0.001 | 0.89 (0.86, 0.92) | <0.001 | 0.97 (0.94, 1.01) | 0.290 | 0.88 (0.82, 0.93) | <0.001 |
| Total cholesterol minus HDL-C | 0.93 (0.91, 0.94) | <0.001 | 0.88 (0.85, 0.91) | <0.001 | 0.98 (0.95, 1.01) | 0.312 | 0.89 (0.83, 0.94) | 0.001 |
| Remnant cholesterol (non-HDL, non-LDL -cholesterol) | 0.94 (0.92, 0.95) | <0.001 | 0.90 (0.87, 0.93) | <0.001 | 0.98 (0.95, 1.01) | 0.418 | 0.91 (0.86, 0.97) | 0.010 |
| VLDL cholesterol | 0.95 (0.94, 0.96) | <0.001 | 0.91 (0.87, 0.94) | <0.001 | 1.00 (0.97, 1.03) | 0.983 | 0.92 (0.86, 0.98) | 0.027 |
| Clinical LDL cholesterol | 0.92 (0.91, 0.93) | <0.001 | 0.87 (0.85, 0.90) | <0.001 | 0.97 (0.94, 1.00) | 0.155 | 0.88 (0.83, 0.94) | <0.001 |
| LDL cholesterol | 0.92 (0.91, 0.93) | <0.001 | 0.87 (0.84, 0.90) | <0.001 | 0.97 (0.94, 1.00) | 0.253 | 0.87 (0.81, 0.92) | <0.001 |
| HDL cholesterol | 0.97 (0.95, 0.98) | <0.001 | 1.02 (0.98, 1.07) | 0.294 | 0.98 (0.95, 1.02) | 0.536 | 0.91 (0.84, 0.99) | 0.038 |
| Total triglycerides | 0.98 (0.97, 0.99) | 0.010 | 0.97 (0.93, 1.00) | 0.098 | 1.04 (1.01, 1.08) | 0.089 | 0.94 (0.88, 1.01) | 0.112 |
| Triglycerides in VLDL | 0.98 (0.96, 0.99) | <0.001 | 0.95 (0.92, 0.99) | 0.019 | 1.04 (1.01, 1.08) | 0.110 | 0.93 (0.87, 0.99) | 0.057 |
| Triglycerides in LDL | 1.01 (0.99, 1.02) | 0.411 | 1.01 (0.98, 1.04) | 0.674 | 1.04 (1.01, 1.07) | 0.089 | 1.00 (0.94, 1.07) | 0.928 |
| Triglycerides in HDL | 1.01 (1.00, 1.03) | 0.050 | 1.03 (1.00, 1.07) | 0.093 | 1.05 (1.02, 1.09) | 0.032 | 0.99 (0.93, 1.06) | 0.785 |
| Total phospholipids in lipoprotein particles | 0.94 (0.93, 0.96) | <0.001 | 0.94 (0.91, 0.98) | 0.003 | 1.00 (0.97, 1.03) | 0.989 | 0.88 (0.83, 0.95) | 0.001 |
| Phospholipids in VLDL | 0.96 (0.95, 0.97) | <0.001 | 0.93 (0.89, 0.96) | <0.001 | 1.02 (0.99, 1.05) | 0.427 | 0.93 (0.87, 0.99) | 0.041 |
| Phospholipids in LDL | 0.92 (0.91, 0.94) | <0.001 | 0.88 (0.85, 0.91) | <0.001 | 0.97 (0.94, 1.00) | 0.253 | 0.88 (0.82, 0.94) | <0.001 |
| Phospholipids in HDL | 0.99 (0.97, 1.00) | 0.110 | 1.06 (1.02, 1.11) | 0.003 | 1.01 (0.97, 1.05) | 0.800 | 0.93 (0.86, 1.00) | 0.086 |
| Total esterified cholesterol | 0.92 (0.91, 0.93) | <0.001 | 0.89 (0.86, 0.92) | <0.001 | 0.97 (0.94, 1.01) | 0.259 | 0.87 (0.82, 0.93) | <0.001 |
| Cholesteryl esters in VLDL | 0.95 (0.93, 0.96) | <0.001 | 0.90 (0.87, 0.93) | <0.001 | 0.99 (0.96, 1.03) | 0.829 | 0.93 (0.87, 0.99) | 0.047 |
| Cholesteryl esters in LDL | 0.92 (0.91, 0.93) | <0.001 | 0.87 (0.84, 0.90) | <0.001 | 0.98 (0.95, 1.01) | 0.318 | 0.87 (0.81, 0.92) | <0.001 |
| Cholesteryl esters in HDL | 0.97 (0.95, 0.98) | <0.001 | 1.02 (0.98, 1.06) | 0.358 | 0.98 (0.94, 1.02) | 0.488 | 0.91 (0.84, 0.98) | 0.027 |
| Total free cholesterol | 0.93 (0.92, 0.94) | <0.001 | 0.89 (0.86, 0.92) | <0.001 | 0.98 (0.95, 1.01) | 0.357 | 0.89 (0.83, 0.95) | 0.001 |
| Free cholesterol in VLDL | 0.95 (0.94, 0.97) | <0.001 | 0.91 (0.88, 0.95) | <0.001 | 1.01 (0.98, 1.04) | 0.694 | 0.92 (0.86, 0.98) | 0.025 |
| Free cholesterol in LDL | 0.92 (0.91, 0.93) | <0.001 | 0.87 (0.84, 0.90) | <0.001 | 0.96 (0.93, 0.99) | 0.101 | 0.88 (0.82, 0.93) | <0.001 |
| Free cholesterol in HDL | 0.98 (0.96, 1.00) | 0.017 | 1.04 (1.00, 1.08) | 0.088 | 0.99 (0.95, 1.03) | 0.800 | 0.95 (0.88, 1.04) | 0.341 |
| Total lipids in lipoprotein particles | 0.94 (0.92, 0.95) | <0.001 | 0.91 (0.88, 0.95) | <0.001 | 1.00 (0.97, 1.03) | 0.966 | 0.88 (0.83, 0.94) | <0.001 |
| Total lipids in VLDL | 0.96 (0.95, 0.97) | <0.001 | 0.93 (0.90, 0.96) | <0.001 | 1.03 (0.99, 1.06) | 0.303 | 0.92 (0.86, 0.98) | 0.023 |
| Total lipids in LDL | 0.92 (0.91, 0.94) | <0.001 | 0.88 (0.85, 0.91) | <0.001 | 0.98 (0.95, 1.01) | 0.303 | 0.87 (0.82, 0.93) | <0.001 |
| Total lipids in HDL | 0.98 (0.96, 0.99) | 0.008 | 1.05 (1.01, 1.09) | 0.037 | 1.00 (0.96, 1.04) | 0.999 | 0.91 (0.85, 0.99) | 0.042 |
| Total concentration of lipoprotein particles | 0.94 (0.93, 0.96) | <0.001 | 0.96 (0.93, 1.00) | 0.071 | 1.00 (0.97, 1.04) | 1.000 | 0.84 (0.78, 0.90) | <0.001 |
| Concentration of VLDL particles | 0.96 (0.95, 0.97) | <0.001 | 0.92 (0.89, 0.95) | <0.001 | 1.01 (0.98, 1.04) | 0.638 | 0.94 (0.88, 1.00) | 0.083 |
| Concentration of LDL particles | 0.93 (0.92, 0.95) | <0.001 | 0.88 (0.85, 0.91) | <0.001 | 0.98 (0.95, 1.01) | 0.303 | 0.9 (0.85, 0.96) | 0.005 |
| Concentration of HDL particles | 0.95 (0.94, 0.97) | <0.001 | 0.99 (0.95, 1.02) | 0.497 | 1.00 (0.97, 1.04) | 0.895 | 0.85 (0.78, 0.91) | <0.001 |
| Average diameter for VLDL particles | 0.96 (0.95, 0.97) | <0.001 | 0.94 (0.90, 0.97) | 0.002 | 1.04 (1.00, 1.07) | 0.209 | 0.88 (0.82, 0.95) | 0.002 |
| Average diameter for LDL particles | 1.00 (0.98, 1.01) | 0.531 | 0.98 (0.95, 1.02) | 0.375 | 0.98 (0.95, 1.01) | 0.357 | 0.96 (0.90, 1.03) | 0.323 |
| Average diameter for HDL particles | 1.03 (1.01, 1.04) | 0.003 | 1.10 (1.06, 1.15) | <0.001 | 0.98 (0.94, 1.02) | 0.418 | 1.09 (1.01, 1.18) | 0.049 |
| Phosphoglycerides | 0.95 (0.94, 0.96) | <0.001 | 0.97 (0.93, 1.00) | 0.091 | 1.01 (0.98, 1.04) | 0.762 | 0.88 (0.82, 0.94) | 0.001 |
| Total cholines | 0.94 (0.93, 0.96) | <0.001 | 0.95 (0.92, 0.99) | 0.016 | 1.00 (0.97, 1.04) | 0.968 | 0.87 (0.82, 0.94) | 0.001 |
| Phosphatidylcholines | 0.95 (0.94, 0.96) | <0.001 | 0.96 (0.93, 1.00) | 0.057 | 1.01 (0.97, 1.04) | 0.800 | 0.87 (0.81, 0.94) | 0.001 |
| Sphingomyelins | 0.95 (0.93, 0.96) | <0.001 | 0.94 (0.91, 0.98) | 0.003 | 0.98 (0.95, 1.02) | 0.427 | 0.92 (0.86, 0.99) | 0.033 |
| Apolipoprotein B | 0.93 (0.92, 0.95) | <0.001 | 0.88 (0.85, 0.91) | <0.001 | 0.98 (0.95, 1.01) | 0.358 | 0.91 (0.85, 0.97) | 0.007 |
| Apolipoprotein A1 | 0.97 (0.96, 0.99) | <0.001 | 1.03 (0.99, 1.07) | 0.190 | 1.01 (0.97, 1.04) | 0.835 | 0.89 (0.82, 0.96) | 0.005 |
| Total fatty acids | 0.96 (0.95, 0.97) | <0.001 | 0.96 (0.93, 0.99) | 0.028 | 1.02 (0.99, 1.06) | 0.351 | 0.91 (0.85, 0.97) | 0.010 |
| Degree of unsaturation | 0.91 (0.90, 0.92) | <0.001 | 0.83 (0.80, 0.86) | <0.001 | 0.93 (0.90, 0.97) | 0.006 | 0.86 (0.80, 0.91) | <0.001 |
| Omega-3 fatty acids | 0.92 (0.91, 0.93) | <0.001 | 0.85 (0.82, 0.88) | <0.001 | 0.98 (0.95, 1.02) | 0.512 | 0.84 (0.79, 0.90) | <0.001 |
| Omega-6 fatty acids | 0.93 (0.92, 0.95) | <0.001 | 0.90 (0.87, 0.93) | <0.001 | 1.00 (0.96, 1.03) | 0.867 | 0.86 (0.81, 0.92) | <0.001 |
| Polyunsaturated fatty acids | 0.92 (0.91, 0.93) | <0.001 | 0.87 (0.84, 0.90) | <0.001 | 0.99 (0.96, 1.02) | 0.769 | 0.84 (0.79, 0.90) | <0.001 |
| Monounsaturated fatty acids | 0.99 (0.98, 1.00) | 0.147 | 1.01 (0.98, 1.05) | 0.493 | 1.04 (1.01, 1.07) | 0.115 | 0.97 (0.91, 1.04) | 0.454 |
| Saturated fatty acids | 0.98 (0.96, 0.99) | 0.001 | 0.99 (0.96, 1.03) | 0.756 | 1.04 (1.00, 1.07) | 0.148 | 0.94 (0.88, 1.00) | 0.078 |
| Linoleic acid | 0.94 (0.92, 0.95) | <0.001 | 0.89 (0.86, 0.92) | <0.001 | 0.99 (0.96, 1.02) | 0.756 | 0.87 (0.81, 0.93) | <0.001 |
| Docosahexaenoic acid | 0.92 (0.9, 0.93) | <0.001 | 0.83 (0.80, 0.87) | <0.001 | 0.97 (0.94, 1.01) | 0.257 | 0.83 (0.77, 0.89) | <0.001 |
| Alanine | 0.98 (0.97, 0.99) | 0.002 | 0.97 (0.94, 1.00) | 0.112 | 0.99 (0.96, 1.03) | 0.814 | 0.99 (0.93, 1.06) | 0.833 |
| Glutamine | 0.97 (0.96, 0.99) | <0.001 | 1.00 (0.96, 1.03) | 0.899 | 0.98 (0.95, 1.01) | 0.318 | 0.94 (0.89, 1.01) | 0.113 |
| Glycine | 1.00 (0.99, 1.01) | 0.930 | 1.02 (0.98, 1.06) | 0.358 | 1.01 (0.97, 1.04) | 0.814 | 0.93 (0.86, 1.00) | 0.092 |
| Histidine | 0.95 (0.94, 0.96) | <0.001 | 0.91 (0.88, 0.94) | <0.001 | 0.99 (0.96, 1.02) | 0.762 | 0.89 (0.83, 0.95) | 0.003 |
| Total concentration of branched-chain amino acids (leucine + isoleucine + valine) | 0.96 (0.95, 0.98) | <0.001 | 0.94 (0.91, 0.98) | 0.003 | 1.00 (0.97, 1.03) | 0.990 | 0.96 (0.90, 1.03) | 0.325 |
| Isoleucine | 0.98 (0.97, 0.99) | 0.005 | 0.97 (0.94, 1.00) | 0.106 | 1.01 (0.98, 1.05) | 0.606 | 0.97 (0.91, 1.04) | 0.466 |
| Leucine | 0.96 (0.95, 0.98) | <0.001 | 0.96 (0.92, 0.99) | 0.026 | 1.01 (0.97, 1.04) | 0.842 | 0.96 (0.90, 1.03) | 0.318 |
| Valine | 0.96 (0.95, 0.97) | <0.001 | 0.93 (0.90, 0.97) | <0.001 | 0.99 (0.96, 1.02) | 0.778 | 0.96 (0.90, 1.03) | 0.328 |
| Phenylalanine | 1.02 (1.01, 1.03) | 0.002 | 1.02 (0.98, 1.05) | 0.368 | 1.03 (1.00, 1.06) | 0.206 | 1.11 (1.06, 1.16) | <0.001 |
| Tyrosine | 0.99 (0.97, 1.00) | 0.063 | 1.02 (0.98, 1.05) | 0.384 | 1.02 (0.98, 1.05) | 0.503 | 1.13 (1.07, 1.20) | <0.001 |
| Glucose | 1.00 (0.99, 1.02) | 0.531 | 0.98 (0.95, 1.02) | 0.384 | 0.99 (0.96, 1.02) | 0.694 | 1.00 (0.94, 1.06) | 0.928 |
| Lactate | 1.02 (1.01, 1.04) | 0.001 | 1.06 (1.03, 1.10) | 0.001 | 1.02 (0.99, 1.05) | 0.358 | 0.99 (0.93, 1.05) | 0.780 |
| Pyruvate | 1.02 (1.00, 1.03) | 0.011 | 1.04 (1.01, 1.08) | 0.034 | 1.01 (0.97, 1.04) | 0.848 | 1.01 (0.95, 1.08) | 0.785 |
| Citrate | 0.98 (0.97, 0.99) | 0.003 | 0.97 (0.93, 1.00) | 0.086 | 0.93 (0.90, 0.96) | 0.002 | 1.02 (0.96, 1.09) | 0.567 |
| 3-Hydroxybutyrate | 1.03 (1.01, 1.04) | <0.001 | 1.05 (1.02, 1.08) | 0.003 | 0.98 (0.95, 1.01) | 0.421 | 1.03 (0.97, 1.09) | 0.455 |
| Acetate | 1.00 (0.99, 1.01) | 0.996 | 0.98 (0.94, 1.01) | 0.226 | 1.01 (0.98, 1.04) | 0.785 | 0.98 (0.91, 1.04) | 0.567 |
| Acetoacetate | 1.02 (1.01, 1.04) | <0.001 | 1.05 (1.02, 1.07) | 0.001 | 0.99 (0.96, 1.02) | 0.711 | 1.01 (0.96, 1.08) | 0.693 |
| Acetone | 1.02 (1.01, 1.04) | <0.001 | 1.04 (1.01, 1.07) | 0.011 | 0.99 (0.96, 1.02) | 0.778 | 0.99 (0.93, 1.06) | 0.812 |
| Creatinine | 1.03 (1.02, 1.05) | <0.001 | 0.98 (0.94, 1.02) | 0.307 | 1.03 (1.00, 1.07) | 0.253 | 1.08 (1.03, 1.14) | 0.007 |
| Albumin | 0.90 (0.89, 0.91) | <0.001 | 0.86 (0.83, 0.89) | <0.001 | 0.94 (0.91, 0.97) | 0.016 | 0.76 (0.71, 0.80) | <0.001 |
| Glycoprotein acetyls | 1.05 (1.03, 1.06) | <0.001 | 1.10 (1.06, 1.14) | <0.001 | 1.05 (1.02, 1.09) | 0.057 | 1.06 (0.99, 1.13) | 0.137 |
| Concentration of chylomicrons and extremely large VLDL particles | 1.00 (0.99, 1.01) | 0.925 | 1.00 (0.97, 1.04) | 0.920 | 1.04 (1.01, 1.08) | 0.089 | 0.98 (0.92, 1.04) | 0.567 |
| Total lipids in chylomicrons and extremely large VLDL | 1.00 (0.98, 1.01) | 0.561 | 1.00 (0.97, 1.03) | 0.951 | 1.04 (1.01, 1.08) | 0.089 | 0.97 (0.91, 1.04) | 0.517 |
| Phospholipids in chylomicrons and extremely large VLDL | 1.00 (0.99, 1.01) | 0.925 | 1.00 (0.97, 1.04) | 0.949 | 1.04 (1.01, 1.08) | 0.089 | 0.98 (0.91, 1.04) | 0.565 |
| Cholesterol in chylomicrons and extremely large VLDL | 0.99 (0.98, 1.01) | 0.466 | 0.99 (0.95, 1.02) | 0.535 | 1.04 (1.01, 1.08) | 0.089 | 0.98 (0.92, 1.05) | 0.634 |
| Cholesteryl esters in chylomicrons and extremely large VLDL | 0.99 (0.98, 1.01) | 0.464 | 0.98 (0.95, 1.02) | 0.365 | 1.04 (1.01, 1.07) | 0.101 | 0.99 (0.93, 1.06) | 0.812 |
| Free cholesterol in chylomicrons and extremely large VLDL | 1.00 (0.98, 1.01) | 0.547 | 1.00 (0.96, 1.03) | 0.836 | 1.04 (1.01, 1.08) | 0.089 | 0.97 (0.91, 1.04) | 0.474 |
| Triglycerides in chylomicrons and extremely large VLDL | 1.00 (0.98, 1.01) | 0.701 | 1.00 (0.97, 1.04) | 0.837 | 1.04 (1.01, 1.08) | 0.089 | 0.97 (0.91, 1.04) | 0.526 |
| Concentration of very large VLDL particles | 0.98 (0.97, 1.00) | 0.011 | 0.96 (0.93, 0.99) | 0.030 | 1.04 (1.00, 1.07) | 0.148 | 0.94 (0.88, 1.01) | 0.121 |
| Total lipids in very large VLDL | 0.98 (0.96, 0.99) | 0.002 | 0.95 (0.92, 0.99) | 0.018 | 1.04 (1.00, 1.07) | 0.148 | 0.94 (0.87, 1.00) | 0.087 |
| Phospholipids in very large VLDL | 0.98 (0.97, 0.99) | 0.003 | 0.95 (0.92, 0.99) | 0.016 | 1.03 (1.00, 1.07) | 0.210 | 0.94 (0.88, 1.01) | 0.137 |
| Cholesterol in very large VLDL | 0.97 (0.95, 0.98) | <0.001 | 0.93 (0.89, 0.96) | <0.001 | 1.02 (0.99, 1.05) | 0.384 | 0.93 (0.87, 1.00) | 0.076 |
| Cholesteryl esters in very large VLDL | 0.96 (0.95, 0.97) | <0.001 | 0.91 (0.88, 0.94) | <0.001 | 1.01 (0.98, 1.05) | 0.641 | 0.93 (0.87, 1.00) | 0.060 |
| Free cholesterol in very large VLDL | 0.97 (0.96, 0.99) | <0.001 | 0.95 (0.91, 0.98) | 0.004 | 1.03 (1.00, 1.06) | 0.253 | 0.94 (0.88, 1.01) | 0.119 |
| Triglycerides in very large VLDL | 0.99 (0.97, 1.00) | 0.046 | 0.97 (0.94, 1.00) | 0.112 | 1.04 (1.01, 1.08) | 0.101 | 0.94 (0.88, 1.01) | 0.116 |
| Concentration of large VLDL particles | 0.97 (0.96, 0.99) | <0.001 | 0.94 (0.90, 0.97) | 0.001 | 1.03 (1.00, 1.06) | 0.253 | 0.93 (0.87, 0.99) | 0.054 |
| Total lipids in large VLDL | 0.96 (0.95, 0.98) | <0.001 | 0.93 (0.89, 0.96) | <0.001 | 1.03 (0.99, 1.06) | 0.270 | 0.91 (0.85, 0.98) | 0.018 |
| Phospholipids in large VLDL | 0.97 (0.96, 0.99) | <0.001 | 0.94 (0.91, 0.97) | 0.002 | 1.03 (1.00, 1.07) | 0.236 | 0.93 (0.87, 1.00) | 0.060 |
| Cholesterol in large VLDL | 0.96 (0.95, 0.97) | <0.001 | 0.91 (0.88, 0.95) | <0.001 | 1.02 (0.98, 1.05) | 0.488 | 0.92 (0.86, 0.98) | 0.025 |
| Cholesteryl esters in large VLDL | 0.95 (0.94, 0.97) | <0.001 | 0.90 (0.87, 0.94) | <0.001 | 1.01 (0.98, 1.04) | 0.800 | 0.92 (0.86, 0.98) | 0.023 |
| Free cholesterol in large VLDL | 0.97 (0.95, 0.98) | <0.001 | 0.93 (0.89, 0.96) | <0.001 | 1.03 (0.99, 1.06) | 0.300 | 0.92 (0.86, 0.99) | 0.036 |
| Triglycerides in large VLDL | 0.97 (0.96, 0.98) | <0.001 | 0.93 (0.90, 0.97) | <0.001 | 1.03 (1.00, 1.06) | 0.253 | 0.91 (0.85, 0.98) | 0.017 |
| Concentration of medium VLDL particles | 0.94 (0.93, 0.95) | <0.001 | 0.89 (0.86, 0.92) | <0.001 | 0.99 (0.96, 1.03) | 0.848 | 0.90 (0.84, 0.96) | 0.004 |
| Total lipids in medium VLDL | 0.94 (0.93, 0.95) | <0.001 | 0.89 (0.86, 0.92) | <0.001 | 1 (0.97, 1.04) | 0.941 | 0.89 (0.83, 0.95) | 0.001 |
| Phospholipids in medium VLDL | 0.94 (0.93, 0.95) | <0.001 | 0.89 (0.86, 0.92) | <0.001 | 0.99 (0.96, 1.03) | 0.829 | 0.90 (0.84, 0.96) | 0.004 |
| Cholesterol in medium VLDL | 0.93 (0.92, 0.94) | <0.001 | 0.88 (0.85, 0.91) | <0.001 | 0.97 (0.94, 1.00) | 0.253 | 0.90 (0.85, 0.96) | 0.005 |
| Cholesteryl esters in medium VLDL | 0.93 (0.92, 0.94) | <0.001 | 0.88 (0.85, 0.91) | <0.001 | 0.96 (0.93, 1.00) | 0.116 | 0.91 (0.86, 0.97) | 0.010 |
| Free cholesterol in medium VLDL | 0.94 (0.92, 0.95) | <0.001 | 0.88 (0.85, 0.91) | <0.001 | 0.99 (0.96, 1.02) | 0.595 | 0.90 (0.84, 0.96) | 0.005 |
| Triglycerides in medium VLDL | 0.96 (0.95, 0.97) | <0.001 | 0.92 (0.89, 0.95) | <0.001 | 1.03 (0.99, 1.06) | 0.300 | 0.90 (0.84, 0.96) | 0.007 |
| Concentration of small VLDL particles | 0.96 (0.95, 0.98) | <0.001 | 0.92 (0.89, 0.95) | <0.001 | 1.02 (0.99, 1.05) | 0.379 | 0.93 (0.88, 1.00) | 0.072 |
| Total lipids in small VLDL | 0.96 (0.95, 0.97) | <0.001 | 0.91 (0.88, 0.95) | <0.001 | 1.02 (0.99, 1.05) | 0.418 | 0.92 (0.86, 0.98) | 0.028 |
| Phospholipids in small VLDL | 0.95 (0.93, 0.96) | <0.001 | 0.89 (0.86, 0.93) | <0.001 | 1.00 (0.97, 1.03) | 0.990 | 0.91 (0.85, 0.97) | 0.013 |
| Cholesterol in small VLDL | 0.95 (0.94, 0.96) | <0.001 | 0.90 (0.87, 0.93) | <0.001 | 1.00 (0.97, 1.03) | 0.948 | 0.92 (0.87, 0.99) | 0.031 |
| Cholesteryl esters in small VLDL | 0.95 (0.94, 0.97) | <0.001 | 0.91 (0.87, 0.94) | <0.001 | 1.00 (0.97, 1.03) | 0.944 | 0.94 (0.88, 1.00) | 0.072 |
| Free cholesterol in small VLDL | 0.94 (0.93, 0.95) | <0.001 | 0.88 (0.85, 0.92) | <0.001 | 0.99 (0.96, 1.02) | 0.665 | 0.91 (0.85, 0.97) | 0.008 |
| Triglycerides in small VLDL | 0.99 (0.97, 1.00) | 0.052 | 0.96 (0.93, 0.99) | 0.032 | 1.04 (1.01, 1.08) | 0.089 | 0.95 (0.89, 1.01) | 0.156 |
| Concentration of very small VLDL particles | 0.97 (0.96, 0.98) | <0.001 | 0.95 (0.92, 0.99) | 0.010 | 1.00 (0.97, 1.04) | 0.884 | 0.99 (0.93, 1.06) | 0.812 |
| Total lipids in very small VLDL | 0.97 (0.96, 0.99) | <0.001 | 0.96 (0.93, 0.99) | 0.026 | 1.01 (0.98, 1.04) | 0.814 | 0.99 (0.93, 1.06) | 0.800 |
| Phospholipids in very small VLDL | 0.98 (0.97, 1.00) | 0.021 | 0.97 (0.94, 1.00) | 0.093 | 1.01 (0.98, 1.04) | 0.649 | 1.01 (0.95, 1.08) | 0.812 |
| Cholesterol in very small VLDL | 0.96 (0.95, 0.97) | <0.001 | 0.94 (0.91, 0.97) | 0.001 | 0.98 (0.95, 1.02) | 0.514 | 0.98 (0.92, 1.04) | 0.560 |
| Cholesteryl esters in very small VLDL | 0.95 (0.94, 0.97) | <0.001 | 0.94 (0.90, 0.97) | <0.001 | 0.98 (0.95, 1.01) | 0.357 | 0.97 (0.91, 1.04) | 0.474 |
| Free cholesterol in very small VLDL | 0.97 (0.96, 0.98) | <0.001 | 0.95 (0.92, 0.98) | 0.005 | 1.00 (0.97, 1.03) | 0.999 | 0.99 (0.93, 1.06) | 0.812 |
| Triglycerides in very small VLDL | 1.01 (1.00, 1.03) | 0.077 | 1.01 (0.98, 1.05) | 0.581 | 1.05 (1.02, 1.08) | 0.057 | 1.01 (0.95, 1.08) | 0.812 |
| Concentration of IDL particles | 0.94 (0.93, 0.95) | <0.001 | 0.91 (0.88, 0.95) | <0.001 | 0.97 (0.94, 1.01) | 0.259 | 0.94 (0.88, 1.00) | 0.082 |
| Total lipids in IDL | 0.93 (0.92, 0.95) | <0.001 | 0.91 (0.88, 0.94) | <0.001 | 0.97 (0.94, 1.00) | 0.211 | 0.92 (0.86, 0.98) | 0.023 |
| Phospholipids in IDL | 0.94 (0.93, 0.95) | <0.001 | 0.91 (0.88, 0.94) | <0.001 | 0.97 (0.94, 1.00) | 0.253 | 0.93 (0.87, 1.00) | 0.066 |
| Cholesterol in IDL | 0.93 (0.92, 0.94) | <0.001 | 0.90 (0.87, 0.93) | <0.001 | 0.96 (0.93, 0.99) | 0.110 | 0.91 (0.85, 0.97) | 0.010 |
| Cholesteryl esters in IDL | 0.93 (0.92, 0.94) | <0.001 | 0.90 (0.87, 0.93) | <0.001 | 0.96 (0.93, 0.99) | 0.111 | 0.90 (0.85, 0.97) | 0.007 |
| Free cholesterol in IDL | 0.93 (0.92, 0.94) | <0.001 | 0.90 (0.87, 0.93) | <0.001 | 0.96 (0.93, 0.99) | 0.101 | 0.93 (0.87, 0.99) | 0.046 |
| Triglycerides in IDL | 1.02 (1.00, 1.03) | 0.021 | 1.03 (0.99, 1.06) | 0.144 | 1.04 (1.01, 1.08) | 0.089 | 1.03 (0.97, 1.10) | 0.466 |
| Concentration of large LDL particles | 0.94 (0.92, 0.95) | <0.001 | 0.88 (0.85, 0.91) | <0.001 | 0.98 (0.95, 1.01) | 0.274 | 0.90 (0.85, 0.96) | 0.006 |
| Total lipids in large LDL | 0.93 (0.91, 0.94) | <0.001 | 0.88 (0.85, 0.91) | <0.001 | 0.97 (0.94, 1.00) | 0.253 | 0.87 (0.82, 0.93) | <0.001 |
| Phospholipids in large LDL | 0.92 (0.91, 0.94) | <0.001 | 0.88 (0.85, 0.91) | <0.001 | 0.97 (0.94, 1.00) | 0.217 | 0.88 (0.83, 0.94) | <0.001 |
| Cholesterol in large LDL | 0.92 (0.91, 0.93) | <0.001 | 0.87 (0.84, 0.90) | <0.001 | 0.97 (0.94, 1.00) | 0.209 | 0.87 (0.81, 0.93) | <0.001 |
| Cholesteryl esters in large LDL | 0.92 (0.91, 0.93) | <0.001 | 0.87 (0.84, 0.90) | <0.001 | 0.97 (0.94, 1.00) | 0.253 | 0.86 (0.81, 0.92) | <0.001 |
| Free cholesterol in large LDL | 0.92 (0.91, 0.93) | <0.001 | 0.88 (0.85, 0.91) | <0.001 | 0.96 (0.93, 0.99) | 0.089 | 0.88 (0.82, 0.94) | <0.001 |
| Triglycerides in large LDL | 1.01 (1.00, 1.02) | 0.138 | 1.02 (0.98, 1.05) | 0.352 | 1.04 (1.01, 1.07) | 0.089 | 1.02 (0.95, 1.08) | 0.693 |
| Concentration of medium LDL particles | 0.93 (0.92, 0.94) | <0.001 | 0.87 (0.84, 0.90) | <0.001 | 0.98 (0.95, 1.01) | 0.384 | 0.90 (0.85, 0.96) | 0.005 |
| Total lipids in medium LDL | 0.93 (0.91, 0.94) | <0.001 | 0.87 (0.84, 0.90) | <0.001 | 0.98 (0.95, 1.01) | 0.458 | 0.87 (0.82, 0.93) | <0.001 |
| Phospholipids in medium LDL | 0.93 (0.91, 0.94) | <0.001 | 0.87 (0.84, 0.90) | <0.001 | 0.98 (0.95, 1.01) | 0.379 | 0.87 (0.82, 0.93) | <0.001 |
| Cholesterol in medium LDL | 0.92 (0.91, 0.93) | <0.001 | 0.87 (0.84, 0.90) | <0.001 | 0.98 (0.95, 1.01) | 0.379 | 0.87 (0.81, 0.92) | <0.001 |
| Cholesteryl esters in medium LDL | 0.93 (0.91, 0.94) | <0.001 | 0.87 (0.84, 0.90) | <0.001 | 0.99 (0.96, 1.02) | 0.562 | 0.87 (0.82, 0.93) | <0.001 |
| Free cholesterol in medium LDL | 0.92 (0.91, 0.93) | <0.001 | 0.86 (0.83, 0.89) | <0.001 | 0.97 (0.94, 1.00) | 0.136 | 0.87 (0.81, 0.92) | <0.001 |
| Triglycerides in medium LDL | 1.00 (0.99, 1.01) | 0.811 | 1.00 (0.96, 1.03) | 0.831 | 1.04 (1.01, 1.07) | 0.089 | 0.99 (0.93, 1.06) | 0.812 |
| Concentration of small LDL particles | 0.94 (0.92, 0.95) | <0.001 | 0.88 (0.85, 0.91) | <0.001 | 0.98 (0.95, 1.01) | 0.395 | 0.92 (0.86, 0.98) | 0.017 |
| Total lipids in small LDL | 0.93 (0.92, 0.94) | <0.001 | 0.88 (0.85, 0.91) | <0.001 | 0.98 (0.95, 1.01) | 0.357 | 0.89 (0.83, 0.95) | 0.001 |
| Phospholipids in small LDL | 0.93 (0.92, 0.95) | <0.001 | 0.88 (0.85, 0.91) | <0.001 | 0.97 (0.94, 1.00) | 0.253 | 0.91 (0.85, 0.96) | 0.006 |
| Cholesterol in small LDL | 0.93 (0.91, 0.94) | <0.001 | 0.87 (0.84, 0.90) | <0.001 | 0.98 (0.95, 1.01) | 0.282 | 0.88 (0.83, 0.94) | <0.001 |
| Cholesteryl esters in small LDL | 0.93 (0.92, 0.94) | <0.001 | 0.87 (0.84, 0.90) | <0.001 | 0.98 (0.95, 1.01) | 0.418 | 0.88 (0.83, 0.94) | 0.001 |
| Free cholesterol in small LDL | 0.92 (0.91, 0.93) | <0.001 | 0.87 (0.84, 0.90) | <0.001 | 0.96 (0.93, 0.99) | 0.091 | 0.88 (0.83, 0.94) | 0.001 |
| Triglycerides in small LDL | 1.00 (0.98, 1.01) | 0.619 | 0.99 (0.96, 1.02) | 0.500 | 1.04 (1.01, 1.07) | 0.089 | 0.98 (0.92, 1.04) | 0.567 |
| Concentration of very large HDL particles | 1.00 (0.99, 1.02) | 0.658 | 1.04 (1.00, 1.08) | 0.106 | 0.97 (0.93, 1.01) | 0.298 | 1.10 (1.02, 1.19) | 0.023 |
| Total lipids in very large HDL | 1.01 (1.00, 1.03) | 0.162 | 1.06 (1.01, 1.10) | 0.014 | 0.97 (0.93, 1.01) | 0.253 | 1.12 (1.04, 1.21) | 0.007 |
| Phospholipids in very large HDL | 1.02 (1.00, 1.04) | 0.016 | 1.08 (1.03, 1.12) | 0.001 | 0.97 (0.93, 1.01) | 0.303 | 1.14 (1.06, 1.23) | 0.002 |
| Cholesterol in very large HDL | 1.00 (0.99, 1.02) | 0.925 | 1.04 (0.99, 1.08) | 0.131 | 0.96 (0.92, 1.00) | 0.141 | 1.11 (1.03, 1.20) | 0.020 |
| Cholesteryl esters in very large HDL | 1.00 (0.98, 1.01) | 0.794 | 1.03 (0.99, 1.08) | 0.192 | 0.96 (0.92, 1.00) | 0.148 | 1.09 (1.01, 1.18) | 0.049 |
| Free cholesterol in very large HDL | 1.01 (1.00, 1.03) | 0.097 | 1.05 (1.01, 1.09) | 0.030 | 0.96 (0.93, 1.00) | 0.136 | 1.16 (1.08, 1.24) | <0.001 |
| Triglycerides in very large HDL | 1.01 (1.00, 1.02) | 0.106 | 1.02 (0.98, 1.05) | 0.357 | 1.04 (1.01, 1.07) | 0.103 | 1.03 (0.96, 1.09) | 0.509 |
| Concentration of large HDL particles | 1.00 (0.98, 1.02) | 0.964 | 1.06 (1.02, 1.11) | 0.013 | 0.98 (0.94, 1.02) | 0.418 | 1.04 (0.96, 1.13) | 0.466 |
| Total lipids in large HDL | 1.00 (0.99, 1.02) | 0.836 | 1.08 (1.03, 1.12) | 0.002 | 0.98 (0.94, 1.02) | 0.416 | 1.03 (0.95, 1.11) | 0.608 |
| Phospholipids in large HDL | 1.01 (0.99, 1.02) | 0.365 | 1.09 (1.05, 1.14) | <0.001 | 0.98 (0.95, 1.02) | 0.613 | 1.03 (0.95, 1.12) | 0.503 |
| Cholesterol in large HDL | 1.00 (0.98, 1.01) | 0.701 | 1.06 (1.01, 1.10) | 0.021 | 0.97 (0.93, 1.01) | 0.259 | 1.03 (0.95, 1.12) | 0.567 |
| Cholesteryl esters in large HDL | 0.99 (0.98, 1.01) | 0.589 | 1.05 (1.01, 1.10) | 0.033 | 0.97 (0.93, 1.01) | 0.253 | 1.02 (0.94, 1.11) | 0.634 |
| Free cholesterol in large HDL | 1.00 (0.99, 1.02) | 0.619 | 1.07 (1.03, 1.12) | 0.003 | 0.98 (0.94, 1.01) | 0.379 | 1.06 (0.98, 1.15) | 0.209 |
| Triglycerides in large HDL | 1.02 (1.01, 1.04) | 0.001 | 1.06 (1.02, 1.09) | 0.003 | 1.04 (1.01, 1.07) | 0.101 | 1.03 (0.97, 1.10) | 0.435 |
| Concentration of medium HDL particles | 0.98 (0.97, 1.00) | 0.018 | 1.05 (1.01, 1.09) | 0.032 | 1.01 (0.98, 1.05) | 0.655 | 0.90 (0.83, 0.97) | 0.017 |
| Total lipids in medium HDL | 0.98 (0.97, 1.00) | 0.020 | 1.05 (1.01, 1.09) | 0.015 | 1.02 (0.98, 1.05) | 0.561 | 0.89 (0.83, 0.96) | 0.007 |
| Phospholipids in medium HDL | 0.99 (0.97, 1.00) | 0.149 | 1.06 (1.02, 1.10) | 0.002 | 1.02 (0.99, 1.06) | 0.369 | 0.91 (0.84, 0.98) | 0.020 |
| Cholesterol in medium HDL | 0.98 (0.96, 0.99) | 0.002 | 1.04 (1.00, 1.08) | 0.071 | 1.00 (0.97, 1.04) | 0.956 | 0.89 (0.83, 0.96) | 0.007 |
| Cholesteryl esters in medium HDL | 0.98 (0.96, 0.99) | 0.003 | 1.04 (1.00, 1.08) | 0.071 | 1.00 (0.97, 1.04) | 0.966 | 0.89 (0.83, 0.96) | 0.007 |
| Free cholesterol in medium HDL | 0.98 (0.96, 0.99) | 0.008 | 1.04 (1.00, 1.08) | 0.055 | 1.01 (0.97, 1.04) | 0.829 | 0.91 (0.84, 0.98) | 0.033 |
| Triglycerides in medium HDL | 1.01 (1.00, 1.03) | 0.038 | 1.04 (1.01, 1.08) | 0.032 | 1.05 (1.02, 1.09) | 0.032 | 0.98 (0.92, 1.05) | 0.654 |
| Concentration of small HDL particles | 0.94 (0.93, 0.95) | <0.001 | 0.93 (0.90, 0.96) | <0.001 | 1.01 (0.98, 1.04) | 0.728 | 0.80 (0.75, 0.86) | <0.001 |
| Total lipids in small HDL | 0.95 (0.94, 0.97) | <0.001 | 0.97 (0.94, 1.01) | 0.114 | 1.03 (1.00, 1.06) | 0.253 | 0.82 (0.77, 0.88) | <0.001 |
| Phospholipids in small HDL | 0.97 (0.95, 0.98) | <0.001 | 1.00 (0.96, 1.03) | 0.861 | 1.03 (1.00, 1.07) | 0.186 | 0.84 (0.79, 0.90) | <0.001 |
| Cholesterol in small HDL | 0.94 (0.93, 0.95) | <0.001 | 0.93 (0.90, 0.97) | <0.001 | 1.01 (0.97, 1.04) | 0.807 | 0.80 (0.75, 0.86) | <0.001 |
| Cholesteryl esters in small HDL | 0.94 (0.92, 0.95) | <0.001 | 0.93 (0.90, 0.96) | <0.001 | 1.00 (0.97, 1.04) | 0.949 | 0.80 (0.75, 0.85) | <0.001 |
| Free cholesterol in small HDL | 0.95 (0.94, 0.97) | <0.001 | 0.96 (0.92, 0.99) | 0.028 | 1.02 (0.99, 1.05) | 0.395 | 0.85 (0.79, 0.91) | <0.001 |
| Triglycerides in small HDL | 1.01 (0.99, 1.02) | 0.443 | 1.00 (0.97, 1.04) | 0.886 | 1.06 (1.02, 1.09) | 0.032 | 0.96 (0.90, 1.03) | 0.370 |

COPD, chronic obstructive pulmonary disease; HR, hazard ratio; CI, confidence interval; FDR, false discovery rate; HDL-C, high-density lipoprotein cholesterol; HDL, high-density lipoprotein; LDL, low-density lipoprotein; VLDL, very low-density lipoprotein; IDL, Intermediate Density Lipoprotein.

The models were adjusted for age, sex, ethnicity, educational attainment, occupational status, Townsend deprivation index, smoking status, alcohol consumption, healthy diet, regular exercise, sleep duration, body mass index, and waist circumference.

# **Supplementary Table S19. Mediation proportion of probable sarcopenia in respiratory disease attributed to blood inflammatory markers.**

| **Inflammation markers** | **Direct effect (β) (95% CI)** | **FDR** | **Indirect effect (β) (95% CI)** | **FDR** | **Total effect (β) (95% CI)** | **FDR** | **Mediation proportion (%) (95% CI)** | **FDR** |
| --- | --- | --- | --- | --- | --- | --- | --- | --- |
| Leukocyte count | -9.03 (-10.09, -7.75) | <0.001 | -0.21 (-0.27, -0.15) | <0.001 | -9.24 (-10.29, -7.93) | <0.001 | 2.3 (1.6, 3.0) | <0.001 |
| Neutrophil count | -9.03 (-10.22, -7.88) | <0.001 | -0.19 (-0.25, -0.11) | <0.001 | -9.22 (-10.42, -8.06) | <0.001 | 2.0 (1.4, 2.7) | <0.001 |
| Neutrophil percentage | -9.15 (-10.23, -7.97) | <0.001 | -0.03 (-0.05, -0.01) | <0.001 | -9.18 (-10.25, -8.02) | <0.001 | 0.3 (0.1, 0.5) | <0.001 |
| Monocyte count | -9.07 (-10.22, -7.94) | <0.001 | -0.10 (-0.13, -0.06) | <0.001 | -9.17 (-10.31, -8.06) | <0.001 | 1.0 (0.6, 1.6) | <0.001 |
| Lymphocyte percentage | -9.05 (-10.44, -7.76) | <0.001 | -0.20 (-0.25, -0.14) | <0.001 | -9.25 (-10.63, -7.95) | <0.001 | 2.2 (1.4, 2.9) | <0.001 |
| C-reactive protein | -8.64 (-9.81, -7.51) | <0.001 | -0.76 (-0.85, -0.67) | <0.001 | -9.40 (-10.53, -8.27) | <0.001 | 8.1 (6.7, 9.6) | <0.001 |
| Platelet count | -9.19 (-10.44, -7.98) | <0.001 | -0.04 (-0.07, -0.03) | <0.001 | -9.23 (-10.48, -8.02) | <0.001 | 0.5 (0.3, 0.8) | <0.001 |
| SII | -8.92 (-10.19, -7.53) | <0.001 | -0.18 (-0.23, -0.12) | <0.001 | -9.09 (-10.37, -7.69) | <0.001 | 1.9 (1.5, 2.6) | <0.001 |
| NLR | -9.06 (-10.18, -7.93) | <0.001 | -0.13 (-0.18, -0.10) | <0.001 | -9.19 (-10.29, -8.06) | <0.001 | 1.5 (1.1, 2.0) | <0.001 |
| PLR | -9.25 (-10.26, -8.10) | <0.001 | -0.02 (-0.04, 0.00) | <0.001 | -9.26 (-10.27, -8.13) | <0.001 | 0.2 (0.0, 0.4) | 0.022 |
| LMR | -9.07 (-10.15, -7.49) | <0.001 | -0.09 (-0.13, -0.06) | <0.001 | -9.16 (-10.25, -7.59) | <0.001 | 1.0 (0.6, 1.5) | <0.001 |

CI, confidence interval; FDR, false discovery rate; SII, systemic immune-inflammation index; NLR, neutrophil-to-lymphocyte ratio; PLR, platelet-to-lymphocyte ratio; LMR, lymphocyte-to-monocyte ratio.

The models were adjusted for age, sex, ethnicity, educational attainment, occupational status, Townsend deprivation index, smoking status, alcohol consumption, healthy diet, regular exercise, sleep duration, body mass index, and waist circumference.

# **Supplementary Table S20. Mediation proportion of probable sarcopenia in COPD attributed to blood inflammatory markers.**

| **Inflammation markers** | **Direct effect (β) (95% CI)** | **FDR** | **Indirect effect (β) (95% CI)** | **FDR** | **Total effect (β)**  **(95% CI)** | **FDR** | **Mediation proportion (%) (95% CI)** | **FDR** |
| --- | --- | --- | --- | --- | --- | --- | --- | --- |
| Leukocyte count | -34.52 (-43.95, -23.35) | <0.001 | -1.62 (-2.11, -1.19) | <0.001 | -36.14 (-45.76, -24.70) | <0.001 | 4.5 (3.0, 7.1) | <0.001 |
| Neutrophil count | -34.75 (-45.38, -25.15) | <0.001 | -1.38 (-1.90, -0.85) | <0.001 | -36.13 (-46.95, -26.57) | <0.001 | 3.9 (2.5, 5.9) | <0.001 |
| Neutrophil percentage | -36.41 (-45.91, -26.23) | <0.001 | -0.12 (-0.29, -0.01) | 0.040 | -36.52 (-45.98, -26.43) | <0.001 | 0.3 (0.0, 0.6) | 0.051 |
| Monocyte count | -35.38 (-45.80, -25.71) | <0.001 | -0.74 (-1.07, -0.42) | <0.001 | -36.12 (-46.67, -26.56) | <0.001 | 2.0 (1.1, 3.3) | <0.001 |
| Lymphocyte percentage | -36.05 (-47.59, -24.82) | <0.001 | -0.70 (-1.01, -0.41) | <0.001 | -36.76 (-48.05, -25.56) | <0.001 | 2.0 (1.1, 3.4) | <0.001 |
| C-reactive protein | -32.74 (-43.58, -22.65) | <0.001 | -4.91 (-5.80, -4.20) | <0.001 | -37.65 (-48.63, -27.36) | <0.001 | 12.9 (9.6, 18.2) | <0.001 |
| Platelet count | -36.50 (-48.08, -26.52) | <0.001 | -0.33 (-0.52, -0.17) | <0.001 | -36.83 (-48.44, -26.78) | <0.001 | 1.0 (0.5, 1.8) | <0.001 |
| SII | -34.68 (-46.10, -22.89) | <0.001 | -0.77 (-1.16, -0.51) | <0.001 | -35.45 (-46.82, -23.58) | <0.001 | 2.1 (1.4, 3.4) | <0.001 |
| NLR | -36.02 (-46.13, -26.27) | <0.001 | -0.49 (-0.78, -0.27) | <0.001 | -36.51 (-46.74, -26.66) | <0.001 | 1.4 (0.8, 2.1) | <0.001 |
| PLR | -37.30 (-46.72, -28.42) | <0.001 | 0.27 (0.10, 0.51) | <0.001 | -37.02 (-46.29, -28.24) | <0.001 | — | — |
| LMR | -36.06 (-45.40, -22.29) | <0.001 | -0.28 (-0.49, -0.14) | <0.001 | -36.35 (-45.73, -22.53) | <0.001 | 0.8 (0.4, 1.5) | <0.001 |

COPD, chronic obstructive pulmonary disease; CI, confidence interval; FDR, false discovery rate; SII, systemic immune-inflammation index; NLR, neutrophil-to-lymphocyte ratio; PLR, platelet-to-lymphocyte ratio; LMR, lymphocyte-to-monocyte ratio.

The models were adjusted for age, sex, ethnicity, educational attainment, occupational status, Townsend deprivation index, smoking status, alcohol consumption, healthy diet, regular exercise, sleep duration, body mass index, and waist circumference.

# **Supplementary Table S21. Mediation proportion of probable sarcopenia in asthma attributed to blood inflammatory markers.**

| **Inflammation markers** | **Direct effect (β)**  **(95% CI)** | **FDR** | **Indirect effect (β) (95% CI)** | **FDR** | **Total effect (β)**  **(95% CI)** | **FDR** | **Mediation proportion (%) (95% CI)** | **FDR** |
| --- | --- | --- | --- | --- | --- | --- | --- | --- |
| Leukocyte count | -36.30 (-46.04, -24.73) | <0.001 | -0.58 (-0.83, -0.39) | <0.001 | -36.89 (-46.58, -25.19) | <0.001 | 1.6 (0.9, 2.6) | <0.001 |
| Neutrophil count | -36.47 (-47.38, -26.27) | <0.001 | -0.36 (-0.57, -0.18) | <0.001 | -36.82 (-47.82, -26.57) | <0.001 | 0.9 (0.5, 1.6) | <0.001 |
| Neutrophil percentage | -36.67 (-47.47, -25.48) | <0.001 | 0.10 (-0.02, 0.21) | 0.088 | -36.57 (-47.36, -25.38) | <0.001 | — | — |
| Monocyte count | -36.04 (-45.94, -26.11) | <0.001 | -0.25 (-0.39, -0.12) | <0.001 | -36.30 (-46.19, -26.35) | <0.001 | 0.6 (0.2, 1.3) | <0.001 |
| Lymphocyte percentage | -36.53 (-48.95, -25.25) | <0.001 | -0.45 (-0.69, -0.22) | <0.001 | -36.98 (-49.21, -25.74) | <0.001 | 1.3 (0.6, 2.4) | <0.001 |
| C-reactive protein | -35.57 (-46.26, -25.36) | <0.001 | -1.98 (-2.58, -1.43) | <0.001 | -37.55 (-47.97, -27.18) | <0.001 | 5.2 (3.5, 7.8) | <0.001 |
| Platelet count | -36.40 (-47.81, -25.70) | <0.001 | -0.33 (-0.51, -0.17) | <0.001 | -36.72 (-48.16, -25.96) | <0.001 | 1.0 (0.5, 1.9) | <0.001 |
| SII | -35.24 (-46.65, -22.72) | <0.001 | -0.45 (-0.76, -0.25) | <0.001 | -35.68 (-47.05, -23.11) | <0.001 | 1.2 (0.7, 2.3) | <0.001 |
| NLR | -36.42 (-46.17, -26.09) | <0.001 | -0.19 (-0.41, -0.01) | 0.049 | -36.60 (-46.42, -26.00) | <0.001 | 0.6 (0.1, 1.2) | 0.049 |
| PLR | -37.15 (-46.00, -27.02) | <0.001 | -0.12 (-0.31, 0.02) | 0.100 | -37.27 (-46.08, -27.18) | <0.001 | — | — |
| LMR | -36.13 (-46.16, -21.71) | <0.001 | -0.18 (-0.36, -0.06) | <0.001 | -36.31 (-46.31, -21.83) | <0.001 | 0.5 (0.1, 1.0) | <0.001 |

CI, confidence interval; FDR, false discovery rate; SII, systemic immune-inflammation index; NLR, neutrophil-to-lymphocyte ratio; PLR, platelet-to-lymphocyte ratio; LMR, lymphocyte-to-monocyte ratio.

The models were adjusted for age, sex, ethnicity, educational attainment, occupational status, Townsend deprivation index, smoking status, alcohol consumption, healthy diet, regular exercise, sleep duration, body mass index, and waist circumference.

# **Supplementary Table S22. Mediation proportion of probable sarcopenia in interstitial lung disease attributed to blood inflammatory markers.**

| **Inflammation markers** | **Direct effect (β) (95% CI)** | **FDR** | **Indirect effect (β) (95% CI)** | **FDR** | **Total effect (β)**  **(95% CI)** | **FDR** | **Mediation proportion (%) (95% CI)** | **FDR** |
| --- | --- | --- | --- | --- | --- | --- | --- | --- |
| Leukocyte count | -68.44 (-86.69, -46.52) | <0.001 | -1.20 (-1.66, -0.78) | <0.001 | -69.65 (-87.80, -47.96) | <0.001 | 1.8 (1.0, 2.8) | <0.001 |
| Neutrophil count | -68.12 (-90.70, -52.10) | <0.001 | -1.09 (-1.62, -0.59) | <0.001 | -69.22 (-92.06, -53.29) | <0.001 | 1.6 (1.0, 2.4) | <0.001 |
| Neutrophil percentage | -69.37 (-91.44, -49.37) | <0.001 | -0.16 (-0.50, 0.04) | 0.220 | -69.54 (-91.62, -49.64) | <0.001 | — | — |
| Monocyte count | -68.17 (-90.60, -49.21) | <0.001 | -0.82 (-1.25, -0.42) | <0.001 | -68.99 (-91.45, -50.16) | <0.001 | 1.1 (0.6, 2.0) | <0.001 |
| Lymphocyte percentage | -68.26 (-89.92, -46.54) | <0.001 | -1.10 (-1.68, -0.58) | <0.001 | -69.36 (-90.92, -47.38) | <0.001 | 1.7 (0.9, 2.8) | <0.001 |
| C-reactive protein | -59.59 (-83.06, -40.47) | <0.001 | -6.66 (-8.46, -5.23) | <0.001 | -66.25 (-90.14, -46.75) | <0.001 | 10.0 (7.4, 14.2) | <0.001 |
| Platelet count | -69.44 (-91.76, -52.92) | <0.001 | 0.07 (-0.17, 0.41) | 0.726 | -69.37 (-91.54, -52.90) | <0.001 | — | — |
| SII | -66.95 (-87.43, -44.88) | <0.001 | -0.79 (-1.44, -0.41) | <0.001 | -67.74 (-88.13, -45.55) | <0.001 | 1.2 (0.6, 2.1) | <0.001 |
| NLR | -68.97 (-90.70, -48.21) | <0.001 | -0.77 (-1.28, -0.37) | <0.001 | -69.74 (-91.61, -48.89) | <0.001 | 1.2 (0.6, 1.8) | <0.001 |
| PLR | -71.21 (-90.73, -52.70) | <0.001 | 0.06 (-0.24, 0.39) | 0.740 | -71.15 (-90.64, -52.72) | <0.001 | — | — |
| LMR | -68.88 (-87.11, -44.08) | <0.001 | -0.77 (-1.24, -0.44) | <0.001 | -69.65 (-88.07, -44.93) | <0.001 | 1.1 (0.5, 2.0) | <0.001 |

CI, confidence interval; FDR, false discovery rate; SII, systemic immune-inflammation index; NLR, neutrophil-to-lymphocyte ratio; PLR, platelet-to-lymphocyte ratio; LMR, lymphocyte-to-monocyte ratio.

The models were adjusted for age, sex, ethnicity, educational attainment, occupational status, Townsend deprivation index, smoking status, alcohol consumption, healthy diet, regular exercise, sleep duration, body mass index, and waist circumference.

# **Supplementary Table S23. Mediation proportion of probable sarcopenia in respiratory disease attributed to metabolites (N=166,721).**

| **Metabolites** | **Direct effect (β) (95% CI)** | **FDR** | **Indirect effect (β) (95% CI)** | **FDR** | **Total effect (β)**  **(95% CI)** | **FDR** | **Mediation proportion (%) (95% CI)** | **FDR** |
| --- | --- | --- | --- | --- | --- | --- | --- | --- |
| Total cholesterol | -9.01 (-10.54, -7.51) | <0.001 | -0.20 (-0.27, -0.14) | <0.001 | -9.21 (-10.79, -7.69) | <0.001 | 2.1 (1.5, 3.0) | <0.001 |
| Total cholesterol minus HDL-C | -9.07 (-10.61, -7.57) | <0.001 | -0.18 (-0.25, -0.11) | <0.001 | -9.24 (-10.77, -7.74) | <0.001 | 1.9 (1.2, 2.8) | <0.001 |
| Remnant cholesterol (non-HDL, non-LDL -cholesterol) | -9.06 (-10.62, -7.39) | <0.001 | -0.12 (-0.17, -0.08) | <0.001 | -9.18 (-10.74, -7.53) | <0.001 | 1.2 (0.8, 2.1) | <0.001 |
| Clinical LDL cholesterol | -9.07 (-10.89, -7.26) | <0.001 | -0.22 (-0.29, -0.15) | <0.001 | -9.29 (-11.09, -7.51) | <0.001 | 2.4 (1.5, 3.5) | <0.001 |
| LDL cholesterol | -8.92 (-10.63, -7.48) | <0.001 | -0.19 (-0.26, -0.14) | <0.001 | -9.11 (-10.85, -7.68) | <0.001 | 2.1 (1.5, 2.9) | <0.001 |
| HDL cholesterol | -9.32 (-11.01, -7.93) | <0.001 | -0.05 (-0.09, -0.02) | <0.001 | -9.37 (-11.08, -8.00) | <0.001 | 0.5 (0.3, 1.0) | <0.001 |
| Triglycerides in HDL | -9.20 (-10.84, -7.46) | <0.001 | -0.02 (-0.03, 0.00) | 0.022 | -9.22 (-10.87, -7.48) | <0.001 | — | — |
| Total phospholipids in lipoprotein particles | -9.24 (-10.44, -7.57) | <0.001 | -0.08 (-0.13, -0.04) | <0.001 | -9.32 (-10.50, -7.65) | <0.001 | 0.9 (0.4, 1.4) | <0.001 |
| Phospholipids in LDL | -8.93 (-10.38, -7.28) | <0.001 | -0.17 (-0.24, -0.11) | <0.001 | -9.10 (-10.51, -7.42) | <0.001 | 1.9 (1.2, 2.8) | <0.001 |
| Total esterified cholesterol | -8.96 (-10.25, -7.40) | <0.001 | -0.20 (-0.28, -0.16) | <0.001 | -9.16 (-10.48, -7.64) | <0.001 | 2.2 (1.6, 3.2) | <0.001 |
| Cholesteryl esters in VLDL | -9.29 (-10.89, -7.85) | <0.001 | -0.06 (-0.10, -0.01) | <0.001 | -9.35 (-10.97, -7.88) | <0.001 | 0.6 (0.1, 1.2) | <0.001 |
| Cholesteryl esters in LDL | -9.03 (-10.64, -7.67) | <0.001 | -0.17 (-0.24, -0.11) | <0.001 | -9.21 (-10.77, -7.85) | <0.001 | 1.9 (1.2, 2.7) | <0.001 |
| Cholesteryl esters in HDL | -9.37 (-10.97, -7.80) | <0.001 | -0.05 (-0.10, -0.02) | <0.001 | -9.43 (-11.03, -7.84) | <0.001 | 0.6 (0.2, 1.1) | <0.001 |
| Total free cholesterol | -9.12 (-10.97, -7.60) | <0.001 | -0.16 (-0.23, -0.11) | <0.001 | -9.28 (-11.15, -7.74) | <0.001 | 1.8 (1.2, 2.6) | <0.001 |
| Free cholesterol in LDL | -8.95 (-10.59, -7.20) | <0.001 | -0.24 (-0.31, -0.18) | <0.001 | -9.19 (-10.85, -7.47) | <0.001 | 2.5 (1.9, 3.6) | <0.001 |
| Free cholesterol in HDL | -9.25 (-10.82, -7.34) | <0.001 | -0.03 (-0.05, -0.01) | <0.001 | -9.28 (-10.86, -7.36) | <0.001 | 0.3 (0.1, 0.5) | <0.001 |
| Total lipids in lipoprotein particles | -9.00 (-10.71, -7.09) | <0.001 | -0.10 (-0.15, -0.05) | <0.001 | -9.10 (-10.82, -7.16) | <0.001 | 1.0 (0.5, 1.8) | <0.001 |
| Total lipids in LDL | -9.13 (-10.59, -7.52) | <0.001 | -0.17 (-0.24, -0.11) | <0.001 | -9.31 (-10.79, -7.65) | <0.001 | 1.9 (1.1, 2.8) | <0.001 |
| Total lipids in HDL | -9.23 (-10.72, -7.44) | <0.001 | -0.03 (-0.06, -0.01) | <0.001 | -9.26 (-10.75, -7.45) | <0.001 | 0.3 (0.1, 0.6) | <0.001 |
| Total concentration of lipoprotein particles | -9.19 (-10.65, -7.38) | <0.001 | -0.11 (-0.16, -0.06) | <0.001 | -9.30 (-10.77, -7.52) | <0.001 | 1.1 (0.6, 1.9) | <0.001 |
| Concentration of LDL particles | -9.05 (-10.89, -7.41) | <0.001 | -0.11 (-0.16, -0.05) | <0.001 | -9.16 (-11.00, -7.50) | <0.001 | 1.2 (0.6, 1.9) | <0.001 |
| Concentration of HDL particles | -9.21 (-11.04, -7.75) | <0.001 | -0.08 (-0.12, -0.04) | <0.001 | -9.28 (-11.12, -7.81) | <0.001 | 0.8 (0.5, 1.4) | <0.001 |
| Phosphoglycerides | -9.29 (-11.12, -7.88) | <0.001 | -0.05 (-0.09, -0.02) | <0.001 | -9.35 (-11.18, -7.94) | <0.001 | 0.5 (0.2, 1.0) | <0.001 |
| Total cholines | -9.19 (-11.00, -7.70) | <0.001 | -0.08 (-0.12, -0.05) | <0.001 | -9.26 (-11.09, -7.77) | <0.001 | 0.8 (0.5, 1.3) | <0.001 |
| Phosphatidylcholines | -9.24 (-10.96, -7.77) | <0.001 | -0.06 (-0.11, -0.03) | <0.001 | -9.30 (-11.02, -7.83) | <0.001 | 0.6 (0.3, 1.1) | <0.001 |
| Sphingomyelins | -9.14 (-10.95, -7.44) | <0.001 | -0.09 (-0.14, -0.06) | <0.001 | -9.23 (-11.05, -7.54) | <0.001 | 1.0 (0.6, 1.6) | <0.001 |
| Apolipoprotein B | -9.04 (-10.47, -7.55) | <0.001 | -0.11 (-0.18, -0.06) | <0.001 | -9.15 (-10.56, -7.65) | <0.001 | 1.2 (0.6, 2.2) | <0.001 |
| Apolipoprotein A1 | -9.27 (-10.63, -7.58) | <0.001 | -0.04 (-0.07, -0.01) | <0.001 | -9.31 (-10.65, -7.60) | <0.001 | 0.4 (0.1, 0.8) | <0.001 |
| Degree of unsaturation | -9.04 (-10.78, -7.34) | <0.001 | -0.28 (-0.37, -0.19) | <0.001 | -9.32 (-11.04, -7.65) | <0.001 | 3.1 (2.1, 4.3) | <0.001 |
| Omega-3 fatty acids | -9.10 (-10.70, -7.33) | <0.001 | -0.14 (-0.20, -0.07) | <0.001 | -9.24 (-10.90, -7.48) | <0.001 | 1.5 (0.8, 2.5) | <0.001 |
| Omega-6 fatty acids | -9.00 (-10.96, -7.00) | <0.001 | -0.08 (-0.13, -0.03) | <0.001 | -9.08 (-11.06, -7.08) | <0.001 | 0.9 (0.3, 1.6) | <0.001 |
| Polyunsaturated fatty acids | -9.12 (-10.78, -7.68) | <0.001 | -0.12 (-0.20, -0.05) | <0.001 | -9.24 (-10.94, -7.81) | <0.001 | 1.3 (0.6, 2.2) | <0.001 |
| Linoleic acid | -9.02 (-10.52, -7.22) | <0.001 | -0.09 (-0.14, -0.03) | <0.001 | -9.11 (-10.60, -7.33) | <0.001 | 1.0 (0.3, 1.6) | <0.001 |
| Docosahexaenoic acid | -9.03 (-10.64, -7.49) | <0.001 | -0.20 (-0.27, -0.13) | <0.001 | -9.23 (-10.84, -7.66) | <0.001 | 2.1 (1.5, 2.9) | <0.001 |
| Alanine | -9.33 (-11.32, -7.73) | <0.001 | -0.02 (-0.04, 0.00) | 0.061 | -9.35 (-11.34, -7.74) | <0.001 | 0.2 (0.0, 0.5) | 0.061 |
| Glutamine | -9.29 (-10.93, -7.90) | <0.001 | -0.05 (-0.08, -0.02) | <0.001 | -9.34 (-10.99, -7.94) | <0.001 | 0.5 (0.3, 0.9) | <0.001 |
| Histidine | -9.33 (-10.77, -7.66) | <0.001 | -0.06 (-0.10, -0.02) | <0.001 | -9.40 (-10.87, -7.73) | <0.001 | 0.7 (0.3, 1.3) | <0.001 |
| Phenylalanine | -9.28 (-10.69, -7.82) | <0.001 | -0.05 (-0.08, -0.02) | <0.001 | -9.33 (-10.73, -7.86) | <0.001 | 0.5 (0.2, 0.9) | <0.001 |
| Citrate | -9.08 (-10.88, -7.59) | <0.001 | -0.07 (-0.12, -0.02) | 0.022 | -9.15 (-10.95, -7.65) | <0.001 | 0.7 (0.2, 1.4) | 0.021 |
| Acetoacetate | -9.25 (-10.63, -7.89) | <0.001 | -0.02 (-0.04, 0.00) | 0.022 | -9.27 (-10.66, -7.91) | <0.001 | 0.2 (0.0, 0.5) | 0.021 |
| Albumin | -8.88 (-10.55, -7.47) | <0.001 | -0.52 (-0.61, -0.43) | <0.001 | -9.40 (-11.03, -7.98) | <0.001 | 5.6 (4.1, 7.2) | <0.001 |
| Glycoprotein acetyls | -9.15 (-10.85, -7.52) | <0.001 | -0.15 (-0.20, -0.10) | <0.001 | -9.30 (-10.97, -7.67) | <0.001 | 1.6 (1.0, 2.5) | <0.001 |
| Concentration of medium VLDL particles | -9.30 (-10.54, -7.94) | <0.001 | -0.06 (-0.10, -0.01) | 0.041 | -9.36 (-10.60, -8.00) | <0.001 | 0.6 (0.1, 1.1) | 0.041 |
| Phospholipids in medium VLDL | -9.18 (-10.55, -7.44) | <0.001 | -0.06 (-0.12, 0.00) | 0.081 | -9.24 (-10.59, -7.47) | <0.001 | 0.6 (0.0, 1.3) | 0.081 |
| Cholesterol in medium VLDL | -9.36 (-10.84, -7.37) | <0.001 | -0.14 (-0.19, -0.08) | <0.001 | -9.50 (-10.95, -7.52) | <0.001 | 1.5 (0.8, 2.1) | <0.001 |
| Cholesteryl esters in medium VLDL | -9.03 (-10.51, -7.38) | <0.001 | -0.17 (-0.23, -0.10) | <0.001 | -9.20 (-10.68, -7.51) | <0.001 | 1.9 (1.1, 2.6) | <0.001 |
| Free cholesterol in medium VLDL | -9.06 (-10.79, -7.44) | <0.001 | -0.09 (-0.14, -0.04) | <0.001 | -9.15 (-10.86, -7.53) | <0.001 | 0.9 (0.4, 1.5) | <0.001 |
| Free cholesterol in small VLDL | -9.24 (-11.22, -7.71) | <0.001 | -0.08 (-0.13, -0.03) | <0.001 | -9.32 (-11.31, -7.79) | <0.001 | 0.8 (0.3, 1.4) | <0.001 |
| Cholesterol in very small VLDL | -8.99 (-10.71, -7.32) | <0.001 | -0.06 (-0.09, -0.02) | <0.001 | -9.05 (-10.77, -7.37) | <0.001 | 0.6 (0.3, 1.0) | <0.001 |
| Cholesteryl esters in very small VLDL | -9.14 (-10.62, -7.36) | <0.001 | -0.08 (-0.12, -0.04) | <0.001 | -9.22 (-10.69, -7.45) | <0.001 | 0.8 (0.4, 1.3) | <0.001 |
| Concentration of IDL particles | -9.10 (-10.53, -7.48) | <0.001 | -0.13 (-0.18, -0.07) | <0.001 | -9.23 (-10.69, -7.57) | <0.001 | 1.3 (0.8, 2.0) | <0.001 |
| Total lipids in IDL | -8.97 (-10.73, -7.35) | <0.001 | -0.16 (-0.22, -0.11) | <0.001 | -9.12 (-10.92, -7.48) | <0.001 | 1.7 (1.1, 2.4) | <0.001 |
| Phospholipids in IDL | -9.16 (-11.20, -7.46) | <0.001 | -0.12 (-0.18, -0.07) | <0.001 | -9.28 (-11.32, -7.63) | <0.001 | 1.3 (0.8, 1.9) | <0.001 |
| Cholesterol in IDL | -9.14 (-10.61, -7.64) | <0.001 | -0.19 (-0.26, -0.14) | <0.001 | -9.34 (-10.85, -7.83) | <0.001 | 2.1 (1.4, 3.0) | <0.001 |
| Cholesteryl esters in IDL | -9.12 (-10.73, -7.66) | <0.001 | -0.20 (-0.27, -0.14) | <0.001 | -9.32 (-10.94, -7.83) | <0.001 | 2.1 (1.4, 3.0) | <0.001 |
| Free cholesterol in IDL | -9.17 (-10.74, -7.41) | <0.001 | -0.18 (-0.26, -0.13) | <0.001 | -9.34 (-10.91, -7.57) | <0.001 | 1.9 (1.3, 2.8) | <0.001 |
| Triglycerides in IDL | -9.44 (-11.07, -7.64) | <0.001 | -0.03 (-0.06, -0.01) | <0.001 | -9.47 (-11.11, -7.67) | <0.001 | 0.3 (0.1, 0.6) | <0.001 |
| Concentration of large LDL particles | -9.30 (-10.74, -8.13) | <0.001 | -0.10 (-0.15, -0.05) | <0.001 | -9.40 (-10.85, -8.22) | <0.001 | 1.0 (0.5, 1.7) | <0.001 |
| Total lipids in large LDL | -9.11 (-10.79, -7.60) | <0.001 | -0.18 (-0.25, -0.13) | <0.001 | -9.29 (-10.98, -7.77) | <0.001 | 2.0 (1.3, 2.7) | <0.001 |
| Phospholipids in large LDL | -9.07 (-10.57, -7.38) | <0.001 | -0.18 (-0.26, -0.12) | <0.001 | -9.26 (-10.76, -7.59) | <0.001 | 2.0 (1.3, 2.9) | <0.001 |
| Cholesterol in large LDL | -8.92 (-10.57, -7.58) | <0.001 | -0.20 (-0.28, -0.15) | <0.001 | -9.12 (-10.79, -7.78) | <0.001 | 2.2 (1.5, 3.2) | <0.001 |
| Cholesteryl esters in large LDL | -9.06 (-10.63, -7.34) | <0.001 | -0.19 (-0.26, -0.14) | <0.001 | -9.25 (-10.81, -7.53) | <0.001 | 2.1 (1.4, 3.2) | <0.001 |
| Free cholesterol in large LDL | -9.14 (-10.63, -7.67) | <0.001 | -0.24 (-0.32, -0.16) | <0.001 | -9.38 (-10.86, -7.90) | <0.001 | 2.6 (1.6, 3.5) | <0.001 |
| Concentration of medium LDL particles | -9.11 (-10.76, -7.38) | <0.001 | -0.12 (-0.18, -0.06) | <0.001 | -9.22 (-10.88, -7.48) | <0.001 | 1.3 (0.7, 2.0) | <0.001 |
| Total lipids in medium LDL | -9.14 (-10.68, -7.58) | <0.001 | -0.13 (-0.20, -0.06) | <0.001 | -9.27 (-10.81, -7.67) | <0.001 | 1.4 (0.7, 2.3) | <0.001 |
| Phospholipids in medium LDL | -9.31 (-10.87, -8.02) | <0.001 | -0.13 (-0.19, -0.07) | <0.001 | -9.44 (-11.01, -8.16) | <0.001 | 1.4 (0.8, 2.2) | <0.001 |
| Cholesterol in medium LDL | -9.07 (-10.43, -7.68) | <0.001 | -0.16 (-0.24, -0.10) | <0.001 | -9.22 (-10.61, -7.81) | <0.001 | 1.8 (1.0, 2.5) | <0.001 |
| Cholesteryl esters in medium LDL | -9.03 (-10.66, -7.25) | <0.001 | -0.12 (-0.18, -0.06) | <0.001 | -9.15 (-10.77, -7.40) | <0.001 | 1.3 (0.6, 2.2) | <0.001 |
| Free cholesterol in medium LDL | -8.96 (-10.41, -7.41) | <0.001 | -0.21 (-0.29, -0.13) | <0.001 | -9.17 (-10.64, -7.61) | <0.001 | 2.3 (1.3, 3.2) | <0.001 |
| Concentration of small LDL particles | -9.04 (-10.65, -7.47) | <0.001 | -0.09 (-0.13, -0.03) | <0.001 | -9.13 (-10.70, -7.58) | <0.001 | 1.0 (0.3, 1.5) | <0.001 |
| Total lipids in small LDL | -9.09 (-10.54, -7.46) | <0.001 | -0.11 (-0.17, -0.05) | <0.001 | -9.20 (-10.64, -7.58) | <0.001 | 1.2 (0.6, 1.9) | <0.001 |
| Phospholipids in small LDL | -9.08 (-10.68, -7.30) | <0.001 | -0.11 (-0.16, -0.06) | <0.001 | -9.18 (-10.80, -7.41) | <0.001 | 1.1 (0.6, 2.0) | <0.001 |
| Cholesterol in small LDL | -9.04 (-10.58, -7.71) | <0.001 | -0.14 (-0.22, -0.08) | <0.001 | -9.18 (-10.69, -7.85) | <0.001 | 1.5 (0.9, 2.4) | <0.001 |
| Cholesteryl esters in small LDL | -9.09 (-10.97, -7.42) | <0.001 | -0.11 (-0.17, -0.05) | <0.001 | -9.20 (-11.09, -7.54) | <0.001 | 1.2 (0.5, 2.0) | <0.001 |
| Free cholesterol in small LDL | -9.16 (-10.88, -7.27) | <0.001 | -0.19 (-0.28, -0.13) | <0.001 | -9.36 (-11.07, -7.44) | <0.001 | 2.0 (1.3, 3.1) | <0.001 |
| Concentration of medium HDL particles | -9.27 (-10.67, -7.63) | <0.001 | -0.02 (-0.04, 0.00) | 0.022 | -9.29 (-10.69, -7.66) | <0.001 | 0.2 (0.0, 0.5) | 0.021 |
| Total lipids in medium HDL | -9.26 (-10.77, -7.82) | <0.001 | -0.01 (-0.03, 0.00) | 0.022 | -9.28 (-10.78, -7.83) | <0.001 | 0.2 (0.0, 0.4) | 0.021 |
| Cholesterol in medium HDL | -9.35 (-10.85, -7.95) | <0.001 | -0.03 (-0.06, -0.01) | <0.001 | -9.38 (-10.88, -7.99) | <0.001 | 0.3 (0.1, 0.7) | <0.001 |
| Cholesteryl esters in medium HDL | -9.17 (-10.71, -7.28) | <0.001 | -0.03 (-0.06, -0.01) | <0.001 | -9.20 (-10.74, -7.31) | <0.001 | 0.3 (0.1, 0.6) | <0.001 |
| Free cholesterol in medium HDL | -9.24 (-10.76, -7.70) | <0.001 | -0.03 (-0.05, -0.01) | <0.001 | -9.27 (-10.77, -7.72) | <0.001 | 0.3 (0.1, 0.6) | <0.001 |
| Triglycerides in medium HDL | -9.32 (-11.16, -7.82) | <0.001 | -0.02 (-0.04, 0.00) | 0.041 | -9.34 (-11.18, -7.84) | <0.001 | 0.2 (0.0, 0.5) | 0.041 |
| Concentration of small HDL particles | -9.27 (-10.82, -7.88) | <0.001 | -0.08 (-0.14, -0.03) | <0.001 | -9.35 (-10.91, -7.95) | <0.001 | 0.9 (0.3, 1.4) | <0.001 |
| Cholesterol in small HDL | -9.14 (-10.43, -7.39) | <0.001 | -0.09 (-0.14, -0.04) | <0.001 | -9.23 (-10.53, -7.46) | <0.001 | 1.0 (0.4, 1.6) | <0.001 |
| Cholesteryl esters in small HDL | -9.20 (-11.16, -7.40) | <0.001 | -0.10 (-0.15, -0.06) | <0.001 | -9.30 (-11.26, -7.51) | <0.001 | 1.1 (0.6, 1.7) | <0.001 |
| Free cholesterol in small HDL | -9.29 (-10.72, -7.62) | <0.001 | -0.04 (-0.09, 0.00) | 0.041 | -9.33 (-10.73, -7.67) | <0.001 | 0.4 (0.0, 1.0) | 0.041 |
| Triglycerides in small HDL | -9.26 (-10.87, -7.42) | <0.001 | -0.01 (-0.03, 0.01) | 0.400 | -9.27 (-10.88, -7.42) | <0.001 | — | — |

CI, confidence interval; FDR, false discovery rate; HDL-C, high-density lipoprotein cholesterol; HDL, high-density lipoprotein; LDL, low-density lipoprotein; VLDL, very low-density lipoprotein; IDL, Intermediate Density Lipoprotein.

The models were adjusted for age, sex, ethnicity, educational attainment, occupational status, Townsend deprivation index, smoking status, alcohol consumption, healthy diet, regular exercise, sleep duration, body mass index, and waist circumference.

# **Supplementary Table S24. Mediation proportion of probable sarcopenia in COPD attributed to metabolites (N=166,721).**

| **Metabolites** | **Direct effect (β) (95% CI)** | **FDR** | **Indirect effect (β) (95% CI)** | **FDR** | **Total effect (β)**  **(95% CI)** | **FDR** | **Mediation proportion (%) (95% CI)** | **FDR** |
| --- | --- | --- | --- | --- | --- | --- | --- | --- |
| Total cholesterol | -38.33 (-52.76, -24.80) | <0.001 | -1.01 (-1.48, -0.67) | <0.001 | -39.34 (-53.76, -25.67) | <0.001 | 2.5 (1.5, 4.5) | <0.001 |
| Total cholesterol minus HDL-C | -38.11 (-51.69, -23.58) | <0.001 | -1.03 (-1.51, -0.60) | <0.001 | -39.15 (-52.97, -24.64) | <0.001 | 2.6 (1.4, 4.6) | <0.001 |
| Remnant cholesterol (non-HDL, non-LDL -cholesterol) | -38.17 (-52.50, -23.82) | <0.001 | -0.69 (-1.05, -0.37) | <0.001 | -38.86 (-53.24, -24.62) | <0.001 | 1.7 (0.9, 3.6) | <0.001 |
| Clinical LDL cholesterol | -38.72 (-55.69, -23.93) | <0.001 | -1.24 (-1.77, -0.80) | <0.001 | -39.96 (-56.80, -25.51) | <0.001 | 3.1 (1.7, 6.0) | <0.001 |
| LDL cholesterol | -37.06 (-52.59, -24.56) | <0.001 | -1.14 (-1.60, -0.77) | <0.001 | -38.21 (-53.82, -25.75) | <0.001 | 3.0 (1.9, 5.1) | <0.001 |
| HDL cholesterol | -40.91 (-55.29, -27.91) | <0.001 | 0.16 (-0.09, 0.39) | 0.212 | -40.75 (-55.00, -27.69) | <0.001 | — | — |
| Triglycerides in HDL | -39.47 (-54.64, -23.25) | <0.001 | -0.12 (-0.29, 0.01) | 0.112 | -39.59 (-54.88, -23.44) | <0.001 | — | — |
| Total phospholipids in lipoprotein particles | -39.97 (-50.53, -26.64) | <0.001 | -0.28 (-0.51, -0.09) | <0.001 | -40.26 (-50.75, -26.84) | <0.001 | 0.7 (0.2, 1.3) | <0.001 |
| Phospholipids in LDL | -37.25 (-50.73, -23.30) | <0.001 | -1.03 (-1.59, -0.60) | <0.001 | -38.28 (-51.62, -24.10) | <0.001 | 2.6 (1.5, 4.7) | <0.001 |
| Total esterified cholesterol | -37.69 (-49.65, -24.11) | <0.001 | -1.02 (-1.45, -0.72) | <0.001 | -38.70 (-51.13, -25.45) | <0.001 | 2.6 (1.7, 4.8) | <0.001 |
| Cholesteryl esters in VLDL | -40.45 (-54.22, -27.86) | <0.001 | -0.39 (-0.73, -0.08) | <0.001 | -40.84 (-54.78, -28.29) | <0.001 | 0.9 (0.2, 1.9) | <0.001 |
| Cholesteryl esters in LDL | -37.75 (-52.33, -25.44) | <0.001 | -1.04 (-1.56, -0.59) | <0.001 | -38.79 (-53.25, -26.66) | <0.001 | 2.6 (1.5, 4.7) | <0.001 |
| Cholesteryl esters in HDL | -41.62 (-58.36, -26.64) | <0.001 | 0.16 (-0.08, 0.39) | 0.196 | -41.46 (-58.22, -26.42) | <0.001 | — | — |
| Total free cholesterol | -38.89 (-54.09, -24.61) | <0.001 | -0.87 (-1.33, -0.55) | <0.001 | -39.76 (-54.83, -25.32) | <0.001 | 2.2 (1.2, 3.9) | <0.001 |
| Free cholesterol in LDL | -37.58 (-53.20, -23.30) | <0.001 | -1.39 (-1.87, -0.92) | <0.001 | -38.98 (-54.89, -25.25) | <0.001 | 3.4 (2.3, 6.1) | <0.001 |
| Free cholesterol in HDL | -40.00 (-54.81, -24.23) | <0.001 | 0.22 (0.01, 0.47) | 0.072 | -39.78 (-54.62, -23.97) | <0.001 | — | — |
| Total lipids in lipoprotein particles | -37.67 (-53.52, -20.94) | <0.001 | -0.47 (-0.82, -0.23) | <0.001 | -38.14 (-54.09, -21.23) | <0.001 | 1.1 (0.6, 2.9) | <0.001 |
| Total lipids in LDL | -38.95 (-53.02, -24.61) | <0.001 | -1.04 (-1.53, -0.63) | <0.001 | -39.99 (-54.23, -25.59) | <0.001 | 2.6 (1.6, 5.0) | <0.001 |
| Total lipids in HDL | -39.65 (-53.49, -24.25) | <0.001 | 0.19 (0.02, 0.41) | 0.025 | -39.45 (-53.32, -24.06) | <0.001 | — | — |
| Total concentration of lipoprotein particles | -40.02 (-53.60, -24.08) | <0.001 | -0.25 (-0.54, 0.02) | 0.092 | -40.27 (-53.89, -24.52) | <0.001 | — | — |
| Concentration of LDL particles | -38.10 (-54.12, -23.81) | <0.001 | -0.73 (-1.13, -0.36) | <0.001 | -38.83 (-54.71, -24.34) | <0.001 | 1.8 (0.9, 3.8) | <0.001 |
| Concentration of HDL particles | -39.91 (-57.26, -26.31) | <0.001 | -0.08 (-0.33, 0.12) | 0.614 | -39.99 (-57.21, -26.37) | <0.001 | — | — |
| Phosphoglycerides | -40.59 (-55.12, -27.99) | <0.001 | -0.12 (-0.31, 0.02) | 0.112 | -40.72 (-55.22, -28.03) | <0.001 | — | — |
| Total cholines | -39.22 (-52.49, -25.90) | <0.001 | -0.23 (-0.50, -0.06) | 0.025 | -39.45 (-52.76, -26.15) | <0.001 | 0.6 (0.2, 1.4) | 0.025 |
| Phosphatidylcholines | -39.92 (-54.39, -26.79) | <0.001 | -0.15 (-0.39, -0.01) | 0.025 | -40.08 (-54.43, -26.93) | <0.001 | — | — |
| Sphingomyelins | -38.96 (-55.21, -24.01) | <0.001 | -0.35 (-0.63, -0.14) | <0.001 | -39.32 (-55.48, -24.39) | <0.001 | 0.8 (0.3, 2.0) | <0.001 |
| Apolipoprotein B | -38.11 (-52.34, -25.18) | <0.001 | -0.73 (-1.19, -0.35) | <0.001 | -38.84 (-53.10, -25.84) | <0.001 | 1.8 (0.9, 4.0) | <0.001 |
| Apolipoprotein A1 | -40.62 (-52.57, -25.46) | <0.001 | 0.12 (-0.10, 0.35) | 0.231 | -40.50 (-52.22, -25.39) | <0.001 | — | — |
| Degree of unsaturation | -38.53 (-55.15, -23.74) | <0.001 | -2.05 (-2.76, -1.25) | <0.001 | -40.58 (-57.14, -25.97) | <0.001 | 5.0 (3.2, 8.9) | <0.001 |
| Omega-3 fatty acids | -38.36 (-54.71, -21.82) | <0.001 | -0.97 (-1.43, -0.48) | <0.001 | -39.33 (-55.88, -22.82) | <0.001 | 2.5 (1.2, 5.1) | <0.001 |
| Omega-6 fatty acids | -37.67 (-58.33, -19.82) | <0.001 | -0.48 (-0.78, -0.17) | <0.001 | -38.15 (-58.88, -20.34) | <0.001 | 1.2 (0.3, 2.7) | <0.001 |
| Polyunsaturated fatty acids | -38.36 (-52.75, -26.38) | <0.001 | -0.72 (-1.29, -0.29) | <0.001 | -39.07 (-53.73, -27.18) | <0.001 | 1.8 (0.7, 3.5) | <0.001 |
| Linoleic acid | -37.25 (-49.49, -21.42) | <0.001 | -0.57 (-0.93, -0.19) | <0.001 | -37.82 (-50.26, -22.11) | <0.001 | 1.4 (0.5, 3.1) | <0.001 |
| Docosahexaenoic acid | -37.90 (-51.66, -23.35) | <0.001 | -1.47 (-2.13, -0.95) | <0.001 | -39.37 (-53.36, -24.65) | <0.001 | 3.7 (2.5, 6.1) | <0.001 |
| Alanine | -40.69 (-59.01, -27.00) | <0.001 | -0.08 (-0.28, 0.03) | 0.196 | -40.76 (-59.05, -27.04) | <0.001 | — | — |
| Glutamine | -40.71 (-53.52, -28.07) | <0.001 | 0.01 (-0.23, 0.25) | 0.860 | -40.70 (-53.53, -28.00) | <0.001 | — | — |
| Histidine | -40.13 (-53.35, -25.61) | <0.001 | -0.42 (-0.78, -0.16) | <0.001 | -40.56 (-53.93, -26.08) | <0.001 | 1.0 (0.4, 2.5) | <0.001 |
| Phenylalanine | -40.52 (-53.56, -26.91) | <0.001 | -0.13 (-0.44, 0.12) | 0.373 | -40.65 (-53.64, -27.10) | <0.001 | — | — |
| Citrate | -38.75 (-54.71, -24.82) | <0.001 | -0.35 (-0.86, 0.14) | 0.212 | -39.10 (-55.19, -25.23) | <0.001 | — | — |
| Acetoacetate | -39.94 (-51.92, -27.99) | <0.001 | -0.14 (-0.29, -0.02) | 0.025 | -40.09 (-52.09, -28.14) | <0.001 | 0.3 (0.1, 0.8) | 0.025 |
| Albumin | -37.50 (-52.18, -24.05) | <0.001 | -2.59 (-3.44, -1.91) | <0.001 | -40.10 (-54.70, -26.36) | <0.001 | 6.9 (4.3, 10.3) | <0.001 |
| Glycoprotein acetyls | -38.96 (-53.98, -23.73) | <0.001 | -1.08 (-1.58, -0.62) | <0.001 | -40.04 (-54.90, -24.66) | <0.001 | 2.5 (1.5, 5.3) | <0.001 |
| Concentration of medium VLDL particles | -39.84 (-51.29, -27.98) | <0.001 | -0.38 (-0.74, -0.06) | 0.050 | -40.22 (-51.94, -28.40) | <0.001 | 0.9 (0.1, 1.7) | 0.05 |
| Phospholipids in medium VLDL | -39.19 (-50.85, -23.69) | <0.001 | -0.41 (-0.88, 0.01) | 0.092 | -39.60 (-51.38, -23.75) | <0.001 | 0.9 (0.0, 2.6) | 0.092 |
| Cholesterol in medium VLDL | -40.98 (-52.63, -23.74) | <0.001 | -0.88 (-1.30, -0.54) | <0.001 | -41.87 (-53.56, -24.84) | <0.001 | 2.1 (1.1, 3.9) | <0.001 |
| Cholesteryl esters in medium VLDL | -38.33 (-52.30, -23.80) | <0.001 | -1.06 (-1.49, -0.62) | <0.001 | -39.39 (-53.24, -24.97) | <0.001 | 2.7 (1.5, 4.7) | <0.001 |
| Free cholesterol in medium VLDL | -37.97 (-53.46, -23.17) | <0.001 | -0.58 (-0.94, -0.27) | <0.001 | -38.55 (-53.94, -23.82) | <0.001 | 1.5 (0.7, 3.1) | <0.001 |
| Free cholesterol in small VLDL | -39.53 (-56.58, -26.25) | <0.001 | -0.53 (-0.88, -0.19) | <0.001 | -40.05 (-57.19, -26.88) | <0.001 | 1.2 (0.5, 2.9) | <0.001 |
| Cholesterol in very small VLDL | -37.96 (-50.85, -23.40) | <0.001 | -0.30 (-0.53, -0.10) | <0.001 | -38.26 (-51.16, -23.50) | <0.001 | 0.7 (0.3, 1.7) | <0.001 |
| Cholesteryl esters in very small VLDL | -39.01 (-53.30, -23.59) | <0.001 | -0.38 (-0.69, -0.15) | <0.001 | -39.39 (-53.67, -24.07) | <0.001 | 1.0 (0.4, 2.0) | <0.001 |
| Concentration of IDL particles | -39.16 (-52.52, -24.98) | <0.001 | -0.64 (-1.02, -0.31) | <0.001 | -39.81 (-53.26, -25.66) | <0.001 | 1.6 (0.8, 3.1) | <0.001 |
| Total lipids in IDL | -37.95 (-52.92, -23.70) | <0.001 | -0.81 (-1.25, -0.43) | <0.001 | -38.76 (-53.67, -24.35) | <0.001 | 2.1 (1.1, 3.7) | <0.001 |
| Phospholipids in IDL | -39.32 (-55.68, -23.97) | <0.001 | -0.66 (-1.07, -0.30) | <0.001 | -39.97 (-56.33, -25.03) | <0.001 | 1.6 (0.8, 2.9) | <0.001 |
| Cholesterol in IDL | -39.7 (-52.65, -25.94) | <0.001 | -0.96 (-1.42, -0.60) | <0.001 | -40.66 (-53.62, -26.93) | <0.001 | 2.4 (1.3, 4.0) | <0.001 |
| Cholesteryl esters in IDL | -39.32 (-54.30, -26.36) | <0.001 | -0.99 (-1.52, -0.59) | <0.001 | -40.30 (-55.19, -27.25) | <0.001 | 2.4 (1.4, 4.0) | <0.001 |
| Free cholesterol in IDL | -39.68 (-54.38, -23.31) | <0.001 | -0.92 (-1.48, -0.55) | <0.001 | -40.59 (-55.37, -24.04) | <0.001 | 2.3 (1.2, 3.9) | <0.001 |
| Triglycerides in IDL | -41.59 (-58.19, -25.35) | <0.001 | -0.18 (-0.46, 0.02) | 0.072 | -41.77 (-58.35, -25.50) | <0.001 | — | — |
| Concentration of large LDL particles | -40.28 (-53.47, -28.81) | <0.001 | -0.67 (-1.11, -0.30) | <0.001 | -40.96 (-53.92, -29.41) | <0.001 | 1.6 (0.8, 3.1) | <0.001 |
| Total lipids in large LDL | -39.04 (-54.00, -26.61) | <0.001 | -1.06 (-1.49, -0.68) | <0.001 | -40.10 (-54.99, -27.43) | <0.001 | 2.7 (1.5, 4.3) | <0.001 |
| Phospholipids in large LDL | -38.93 (-53.60, -24.07) | <0.001 | -1.07 (-1.63, -0.65) | <0.001 | -40.01 (-54.52, -25.24) | <0.001 | 2.6 (1.6, 4.9) | <0.001 |
| Cholesterol in large LDL | -37.33 (-52.87, -25.84) | <0.001 | -1.21 (-1.82, -0.80) | <0.001 | -38.54 (-54.24, -26.82) | <0.001 | 3.2 (1.9, 5.2) | <0.001 |
| Cholesteryl esters in large LDL | -38.29 (-52.91, -23.55) | <0.001 | -1.16 (-1.67, -0.71) | <0.001 | -39.45 (-54.10, -24.67) | <0.001 | 2.9 (1.8, 5.6) | <0.001 |
| Free cholesterol in large LDL | -39.26 (-52.21, -26.16) | <0.001 | -1.37 (-1.98, -0.82) | <0.001 | -40.63 (-53.84, -27.24) | <0.001 | 3.4 (1.8, 5.5) | <0.001 |
| Concentration of medium LDL particles | -38.58 (-54.41, -22.59) | <0.001 | -0.78 (-1.28, -0.35) | <0.001 | -39.36 (-55.11, -23.26) | <0.001 | 2.0 (1.2, 3.4) | <0.001 |
| Total lipids in medium LDL | -38.72 (-51.46, -24.94) | <0.001 | -0.82 (-1.27, -0.41) | <0.001 | -39.54 (-52.22, -25.64) | <0.001 | 2.0 (0.9, 4.1) | <0.001 |
| Phospholipids in medium LDL | -40.34 (-55.29, -28.79) | <0.001 | -0.84 (-1.27, -0.41) | <0.001 | -41.19 (-55.98, -29.66) | <0.001 | 2.1 (1.1, 3.4) | <0.001 |
| Cholesterol in medium LDL | -38.18 (-52.72, -25.92) | <0.001 | -1.01 (-1.58, -0.54) | <0.001 | -39.19 (-54.13, -26.86) | <0.001 | 2.7 (1.4, 4.1) | <0.001 |
| Cholesteryl esters in medium LDL | -37.81 (-51.60, -22.85) | <0.001 | -0.77 (-1.19, -0.38) | <0.001 | -38.58 (-52.32, -23.61) | <0.001 | 1.9 (0.9, 4.1) | <0.001 |
| Free cholesterol in medium LDL | -37.60 (-51.28, -23.91) | <0.001 | -1.32 (-1.93, -0.79) | <0.001 | -38.93 (-52.73, -25.25) | <0.001 | 3.4 (1.7, 5.6) | <0.001 |
| Concentration of small LDL particles | -37.60 (-51.99, -24.59) | <0.001 | -0.58 (-0.94, -0.21) | <0.001 | -38.19 (-52.7, -25.19) | <0.001 | 1.5 (0.5, 3.0) | <0.001 |
| Total lipids in small LDL | -38.32 (-51.57, -23.83) | <0.001 | -0.71 (-1.12, -0.33) | <0.001 | -39.03 (-52.51, -24.66) | <0.001 | 1.8 (0.8, 3.4) | <0.001 |
| Phospholipids in small LDL | -38.09 (-53.20, -23.18) | <0.001 | -0.70 (-1.10, -0.36) | <0.001 | -38.79 (-54.18, -23.99) | <0.001 | 1.7 (0.8, 4.5) | <0.001 |
| Cholesterol in small LDL | -38.13 (-51.95, -26.69) | <0.001 | -0.90 (-1.50, -0.50) | <0.001 | -39.03 (-53.08, -27.35) | <0.001 | 2.3 (1.2, 3.8) | <0.001 |
| Cholesteryl esters in small LDL | -38.32 (-57.76, -24.29) | <0.001 | -0.71 (-1.12, -0.31) | <0.001 | -39.04 (-58.39, -24.97) | <0.001 | 1.8 (0.8, 3.5) | <0.001 |
| Free cholesterol in small LDL | -39.42 (-54.25, -22.90) | <0.001 | -1.22 (-1.82, -0.69) | <0.001 | -40.64 (-55.46, -24.28) | <0.001 | 2.9 (1.7, 5.6) | <0.001 |
| Concentration of medium HDL particles | -40.51 (-52.97, -26.71) | <0.001 | 0.17 (0.02, 0.36) | <0.001 | -40.33 (-52.68, -26.56) | <0.001 | — | — |
| Total lipids in medium HDL | -40.01 (-54.41, -27.12) | <0.001 | 0.15 (0.03, 0.30) | <0.001 | -39.86 (-54.20, -27.05) | <0.001 | — | — |
| Cholesterol in medium HDL | -40.96 (-53.22, -28.17) | <0.001 | 0.17 (-0.01, 0.36) | 0.092 | -40.79 (-53.02, -27.99) | <0.001 | — | — |
| Cholesteryl esters in medium HDL | -39.69 (-52.48, -22.78) | <0.001 | 0.18 (0.01, 0.43) | 0.025 | -39.51 (-52.35, -22.57) | <0.001 | — | — |
| Free cholesterol in medium HDL | -40.26 (-53.29, -26.94) | <0.001 | 0.17 (0.01, 0.40) | 0.025 | -40.09 (-53.07, -26.74) | <0.001 | — | — |
| Triglycerides in medium HDL | -40.56 (-56.42, -27.47) | <0.001 | -0.16 (-0.39, -0.02) | 0.025 | -40.71 (-56.59, -27.71) | <0.001 | 0.3 (0.0, 1.2) | 0.025 |
| Concentration of small HDL particles | -40.24 (-55.74, -28.17) | <0.001 | -0.34 (-0.65, -0.10) | <0.001 | -40.58 (-56.06, -28.45) | <0.001 | 0.9 (0.2, 1.5) | <0.001 |
| Cholesterol in small HDL | -38.89 (-51.10, -25.16) | <0.001 | -0.33 (-0.58, -0.11) | <0.001 | -39.23 (-51.49, -25.38) | <0.001 | 0.8 (0.3, 1.7) | <0.001 |
| Cholesteryl esters in small HDL | -39.60 (-57.58, -24.59) | <0.001 | -0.41 (-0.71, -0.15) | <0.001 | -40.01 (-57.94, -24.98) | <0.001 | 1.0 (0.4, 1.9) | <0.001 |
| Free cholesterol in small HDL | -40.76 (-53.11, -25.62) | <0.001 | -0.14 (-0.40, 0.00) | 0.072 | -40.90 (-53.20, -25.79) | <0.001 | 0.3 (0.0, 1.1) | 0.072 |
| Triglycerides in small HDL | -40.31 (-55.04, -24.29) | <0.001 | -0.02 (-0.22, 0.20) | 0.860 | -40.33 (-55.07, -24.41) | <0.001 | — | — |

COPD, chronic obstructive pulmonary disease; CI, confidence interval; FDR, false discovery rate; HDL-C, high-density lipoprotein cholesterol; HDL, high-density lipoprotein; LDL, low-density lipoprotein; VLDL, very low-density lipoprotein; IDL, Intermediate Density Lipoprotein.

The models were adjusted for age, sex, ethnicity, educational attainment, occupational status, Townsend deprivation index, smoking status, alcohol consumption, healthy diet, regular exercise, sleep duration, body mass index, and waist circumference.

# **Supplementary Table S25. Mediation proportion of probable sarcopenia in asthma attributed to metabolites (N=166,721).**

| **Metabolites** | **Direct effect (β)**  **(95% CI)** | **FDR** | **Indirect effect (β) (95% CI)** | **FDR** | **Total effect (β)**  **(95% CI)** | **FDR** | **Mediation proportion (%) (95% CI)** | **FDR** |
| --- | --- | --- | --- | --- | --- | --- | --- | --- |
| Total cholesterol | -39.69 (-52.89, -27.04) | <0.001 | -0.22 (-0.50, 0.07) | 0.323 | -39.91 (-53.21, -27.17) | <0.001 | — | — |
| Total cholesterol minus HDL-C | -39.84 (-52.63, -27.20) | <0.001 | -0.19 (-0.43, 0.05) | 0.387 | -40.03 (-52.79, -27.39) | <0.001 | — | — |
| Remnant cholesterol (non-HDL, non-LDL -cholesterol) | -39.64 (-52.58, -25.71) | <0.001 | -0.11 (-0.35, 0.12) | 0.456 | -39.75 (-52.71, -25.83) | <0.001 | — | — |
| Clinical LDL cholesterol | -40.06 (-56.26, -24.62) | <0.001 | -0.28 (-0.54, 0.00) | 0.235 | -40.34 (-56.35, -25.11) | <0.001 | — | — |
| LDL cholesterol | -38.98 (-52.80, -27.16) | <0.001 | -0.22 (-0.53, 0.01) | 0.265 | -39.21 (-53.03, -27.39) | <0.001 | — | — |
| HDL cholesterol | -40.80 (-54.37, -29.17) | <0.001 | -0.09 (-0.34, 0.11) | 0.659 | -40.89 (-54.44, -29.14) | <0.001 | — | — |
| Triglycerides in HDL | -39.59 (-53.67, -24.74) | <0.001 | -0.20 (-0.40, -0.04) | <0.001 | -39.79 (-53.94, -25.12) | <0.001 | 0.5 (0.1, 1.3) | <0.001 |
| Total phospholipids in lipoprotein particles | -40.81 (-50.45, -26.94) | <0.001 | 0.00 (-0.18, 0.19) | 0.913 | -40.81 (-50.44, -26.91) | <0.001 | — | — |
| Phospholipids in LDL | -38.78 (-51.91, -25.41) | <0.001 | -0.20 (-0.44, 0.04) | 0.319 | -38.98 (-52.02, -25.54) | <0.001 | — | — |
| Total esterified cholesterol | -39.08 (-50.50, -26.22) | <0.001 | -0.23 (-0.52, 0.02) | 0.265 | -39.31 (-50.80, -26.60) | <0.001 | — | — |
| Cholesteryl esters in VLDL | -41.13 (-53.86, -29.28) | <0.001 | -0.02 (-0.17, 0.09) | 0.903 | -41.15 (-53.94, -29.33) | <0.001 | — | — |
| Cholesteryl esters in LDL | -39.59 (-53.16, -28.03) | <0.001 | -0.17 (-0.41, 0.04) | 0.344 | -39.76 (-53.27, -28.18) | <0.001 | — | — |
| Cholesteryl esters in HDL | -41.38 (-56.39, -28.15) | <0.001 | -0.09 (-0.37, 0.13) | 0.584 | -41.47 (-56.52, -28.26) | <0.001 | — | — |
| Total free cholesterol | -40.36 (-55.27, -27.81) | <0.001 | -0.15 (-0.40, 0.08) | 0.456 | -40.51 (-55.40, -28.02) | <0.001 | — | — |
| Free cholesterol in LDL | -39.13 (-52.74, -24.95) | <0.001 | -0.38 (-0.70, -0.06) | 0.132 | -39.51 (-53.16, -25.66) | <0.001 | — | — |
| Free cholesterol in HDL | -39.86 (-54.07, -24.44) | <0.001 | -0.02 (-0.21, 0.14) | 0.903 | -39.89 (-54.04, -24.31) | <0.001 | — | — |
| Total lipids in lipoprotein particles | -38.99 (-53.62, -22.96) | <0.001 | 0.00 (-0.18, 0.15) | 0.960 | -39.00 (-53.73, -22.88) | <0.001 | — | — |
| Total lipids in LDL | -40.66 (-54.03, -27.16) | <0.001 | -0.18 (-0.41, 0.04) | 0.323 | -40.84 (-54.25, -27.35) | <0.001 | — | — |
| Total lipids in HDL | -39.85 (-52.54, -26.42) | <0.001 | 0.00 (-0.14, 0.15) | 0.960 | -39.85 (-52.56, -26.31) | <0.001 | — | — |
| Total concentration of lipoprotein particles | -40.22 (-53.00, -24.82) | <0.001 | -0.01 (-0.23, 0.24) | 0.923 | -40.23 (-53.00, -24.98) | <0.001 | — | — |
| Concentration of LDL particles | -39.64 (-55.22, -25.25) | <0.001 | -0.12 (-0.33, 0.03) | 0.387 | -39.76 (-55.35, -25.38) | <0.001 | — | — |
| Concentration of HDL particles | -40.23 (-56.15, -27.35) | <0.001 | 0.03 (-0.19, 0.21) | 0.903 | -40.20 (-55.98, -27.33) | <0.001 | — | — |
| Phosphoglycerides | -40.95 (-54.86, -29.83) | <0.001 | 0.03 (-0.08, 0.16) | 0.710 | -40.92 (-54.80, -29.79) | <0.001 | — | — |
| Total cholines | -40.14 (-54.41, -27.98) | <0.001 | 0.01 (-0.14, 0.20) | 0.960 | -40.13 (-54.47, -28.03) | <0.001 | — | — |
| Phosphatidylcholines | -40.65 (-55.43, -28.44) | <0.001 | 0.03 (-0.08, 0.21) | 0.759 | -40.62 (-55.26, -28.43) | <0.001 | — | — |
| Sphingomyelins | -39.90 (-54.86, -25.71) | <0.001 | -0.11 (-0.34, 0.05) | 0.430 | -40.01 (-54.89, -25.86) | <0.001 | — | — |
| Apolipoprotein B | -39.48 (-51.68, -26.63) | <0.001 | -0.13 (-0.34, 0.04) | 0.344 | -39.60 (-51.73, -26.81) | <0.001 | — | — |
| Apolipoprotein A1 | -40.56 (-51.64, -26.18) | <0.001 | 0.02 (-0.18, 0.23) | 0.903 | -40.54 (-51.56, -26.17) | <0.001 | — | — |
| Degree of unsaturation | -39.48 (-53.44, -25.50) | <0.001 | -0.72 (-1.06, -0.29) | <0.001 | -40.19 (-53.99, -26.43) | <0.001 | 1.8 (0.7, 3.5) | <0.001 |
| Omega-3 fatty acids | -39.91 (-55.08, -24.89) | <0.001 | -0.10 (-0.30, 0.06) | 0.614 | -40.01 (-55.42, -24.87) | <0.001 | — | — |
| Omega-6 fatty acids | -39.00 (-56.13, -22.32) | <0.001 | -0.02 (-0.17, 0.11) | 0.903 | -39.02 (-56.16, -22.34) | <0.001 | — | — |
| Polyunsaturated fatty acids | -40.17 (-54.11, -27.90) | <0.001 | -0.05 (-0.27, 0.25) | 0.756 | -40.22 (-54.23, -28.00) | <0.001 | — | — |
| Linoleic acid | -38.99 (-51.01, -24.77) | <0.001 | -0.04 (-0.17, 0.09) | 0.710 | -39.03 (-51.13, -24.92) | <0.001 | — | — |
| Docosahexaenoic acid | -39.56 (-53.00, -26.46) | <0.001 | -0.22 (-0.53, 0.05) | 0.323 | -39.77 (-53.39, -26.42) | <0.001 | — | — |
| Alanine | -40.75 (-57.60, -27.26) | <0.001 | -0.01 (-0.14, 0.11) | 0.903 | -40.76 (-57.58, -27.22) | <0.001 | — | — |
| Glutamine | -40.82 (-53.31, -29.37) | <0.001 | -0.15 (-0.38, 0.05) | 0.456 | -40.96 (-53.54, -29.56) | <0.001 | — | — |
| Histidine | -40.87 (-54.05, -27.19) | <0.001 | -0.03 (-0.19, 0.08) | 0.773 | -40.91 (-54.15, -27.28) | <0.001 | — | — |
| Phenylalanine | -40.57 (-52.58, -28.21) | <0.001 | -0.23 (-0.53, -0.01) | 0.202 | -40.81 (-52.70, -28.40) | <0.001 | — | — |
| Citrate | -38.32 (-53.86, -25.62) | <0.001 | -0.87 (-1.35, -0.44) | <0.001 | -39.19 (-54.47, -26.63) | <0.001 | 2.2 (0.8, 4.3) | <0.001 |
| Acetoacetate | -40.37 (-52.91, -29.30) | <0.001 | 0.03 (-0.04, 0.14) | 0.753 | -40.34 (-52.83, -29.26) | <0.001 | — | — |
| Albumin | -39.76 (-53.12, -27.76) | <0.001 | -0.96 (-1.51, -0.46) | <0.001 | -40.72 (-54.12, -28.66) | <0.001 | 2.6 (1.0, 4.3) | <0.001 |
| Glycoprotein acetyls | -39.61 (-53.63, -25.27) | <0.001 | -0.57 (-0.98, -0.24) | <0.001 | -40.18 (-54.02, -25.74) | <0.001 | — | — |
| Concentration of medium VLDL particles | -41.19 (-51.36, -30.39) | <0.001 | -0.01 (-0.14, 0.12) | 0.903 | -41.20 (-51.48, -30.39) | <0.001 | — | — |
| Phospholipids in medium VLDL | -40.45 (-51.01, -25.40) | <0.001 | -0.03 (-0.19, 0.07) | 0.835 | -40.49 (-51.02, -25.43) | <0.001 | — | — |
| Cholesterol in medium VLDL | -42.31 (-54.78, -26.33) | <0.001 | -0.17 (-0.40, 0.02) | 0.265 | -42.48 (-54.91, -26.66) | <0.001 | — | — |
| Cholesteryl esters in medium VLDL | -39.64 (-52.02, -26.14) | <0.001 | -0.29 (-0.57, -0.04) | 0.132 | -39.93 (-52.27, -26.26) | <0.001 | — | — |
| Free cholesterol in medium VLDL | -39.58 (-53.44, -25.42) | <0.001 | -0.06 (-0.22, 0.08) | 0.614 | -39.64 (-53.51, -25.55) | <0.001 | — | — |
| Free cholesterol in small VLDL | -40.73 (-56.38, -28.48) | <0.001 | -0.04 (-0.17, 0.09) | 0.705 | -40.77 (-56.46, -28.53) | <0.001 | — | — |
| Cholesterol in very small VLDL | -38.66 (-52.09, -24.40) | <0.001 | -0.07 (-0.23, 0.07) | 0.659 | -38.73 (-52.15, -24.47) | <0.001 | — | — |
| Cholesteryl esters in very small VLDL | -39.84 (-53.38, -24.83) | <0.001 | -0.12 (-0.35, 0.06) | 0.362 | -39.96 (-53.46, -25.05) | <0.001 | — | — |
| Concentration of IDL particles | -40.00 (-52.97, -25.77) | <0.001 | -0.18 (-0.43, 0.02) | 0.323 | -40.17 (-53.36, -25.88) | <0.001 | — | — |
| Total lipids in IDL | -38.75 (-54.00, -24.51) | <0.001 | -0.25 (-0.58, 0.00) | 0.235 | -39.00 (-54.32, -24.67) | <0.001 | — | — |
| Phospholipids in IDL | -40.33 (-54.85, -26.41) | <0.001 | -0.20 (-0.45, 0.01) | 0.323 | -40.53 (-55.02, -26.83) | <0.001 | — | — |
| Cholesterol in IDL | -40.50 (-51.69, -28.52) | <0.001 | -0.33 (-0.70, -0.03) | 0.132 | -40.83 (-52.07, -28.85) | <0.001 | — | — |
| Cholesteryl esters in IDL | -40.25 (-53.36, -27.91) | <0.001 | -0.34 (-0.69, -0.04) | 0.202 | -40.59 (-53.75, -28.32) | <0.001 | — | — |
| Free cholesterol in IDL | -40.87 (-54.48, -26.27) | <0.001 | -0.34 (-0.71, -0.02) | 0.235 | -41.22 (-54.75, -26.50) | <0.001 | — | — |
| Triglycerides in IDL | -41.56 (-56.46, -25.42) | <0.001 | -0.28 (-0.58, -0.10) | <0.001 | -41.84 (-56.57, -25.68) | <0.001 | — | — |
| Concentration of large LDL particles | -41.42 (-53.58, -31.56) | <0.001 | -0.12 (-0.32, 0.02) | 0.344 | -41.55 (-53.64, -31.72) | <0.001 | — | — |
| Total lipids in large LDL | -40.44 (-53.89, -28.30) | <0.001 | -0.20 (-0.47, 0.06) | 0.362 | -40.64 (-54.08, -28.44) | <0.001 | — | — |
| Phospholipids in large LDL | -40.08 (-53.52, -26.36) | <0.001 | -0.25 (-0.57, -0.02) | 0.202 | -40.33 (-53.62, -26.66) | <0.001 | — | — |
| Cholesterol in large LDL | -38.95 (-52.06, -27.96) | <0.001 | -0.27 (-0.63, 0.00) | 0.235 | -39.22 (-52.33, -28.25) | <0.001 | — | — |
| Cholesteryl esters in large LDL | -39.72 (-52.27, -25.56) | <0.001 | -0.23 (-0.48, 0.02) | 0.265 | -39.95 (-52.58, -25.73) | <0.001 | — | — |
| Free cholesterol in large LDL | -40.92 (-52.60, -29.04) | <0.001 | -0.41 (-0.82, -0.13) | 0.132 | -41.33 (-53.30, -29.39) | <0.001 | — | — |
| Concentration of medium LDL particles | -39.99 (-54.35, -25.39) | <0.001 | -0.11 (-0.29, 0.06) | 0.410 | -40.10 (-54.54, -25.52) | <0.001 | — | — |
| Total lipids in medium LDL | -40.41 (-53.02, -26.75) | <0.001 | -0.10 (-0.35, 0.09) | 0.516 | -40.50 (-53.04, -26.80) | <0.001 | — | — |
| Phospholipids in medium LDL | -41.65 (-55.06, -30.82) | <0.001 | -0.12 (-0.31, 0.06) | 0.456 | -41.77 (-55.09, -31.08) | <0.001 | — | — |
| Cholesterol in medium LDL | -39.91 (-53.15, -28.04) | <0.001 | -0.14 (-0.43, 0.08) | 0.430 | -40.04 (-53.27, -28.19) | <0.001 | — | — |
| Cholesteryl esters in medium LDL | -39.29 (-51.72, -25.09) | <0.001 | -0.09 (-0.26, 0.04) | 0.540 | -39.37 (-51.74, -25.11) | <0.001 | — | — |
| Free cholesterol in medium LDL | -39.26 (-51.52, -26.18) | <0.001 | -0.31 (-0.63, -0.04) | 0.132 | -39.57 (-51.77, -26.62) | <0.001 | — | — |
| Concentration of small LDL particles | -39.14 (-52.85, -26.45) | <0.001 | -0.08 (-0.25, 0.03) | 0.362 | -39.22 (-52.86, -26.65) | <0.001 | — | — |
| Total lipids in small LDL | -39.81 (-51.61, -25.56) | <0.001 | -0.09 (-0.29, 0.11) | 0.614 | -39.90 (-51.69, -25.66) | <0.001 | — | — |
| Phospholipids in small LDL | -39.45 (-53.23, -25.11) | <0.001 | -0.16 (-0.37, 0.00) | 0.235 | -39.61 (-53.44, -25.23) | <0.001 | — | — |
| Cholesterol in small LDL | -39.47 (-52.02, -28.58) | <0.001 | -0.15 (-0.38, 0.10) | 0.41 | -39.62 (-52.02, -28.70) | <0.001 | — | — |
| Cholesteryl esters in small LDL | -39.93 (-56.15, -25.70) | <0.001 | -0.08 (-0.25, 0.05) | 0.584 | -40.01 (-56.31, -25.71) | <0.001 | — | — |
| Free cholesterol in small LDL | -40.75 (-54.73, -24.91) | <0.001 | -0.33 (-0.67, -0.06) | 0.202 | -41.08 (-55.01, -25.26) | <0.001 | — | — |
| Concentration of medium HDL particles | -40.34 (-51.23, -27.37) | <0.001 | 0.05 (-0.06, 0.19) | 0.659 | -40.29 (-51.22, -27.32) | <0.001 | — | — |
| Total lipids in medium HDL | -39.98 (-52.75, -28.18) | <0.001 | 0.05 (-0.04, 0.16) | 0.659 | -39.93 (-52.73, -28.19) | <0.001 | — | — |
| Cholesterol in medium HDL | -40.83 (-52.55, -29.69) | <0.001 | 0.01 (-0.14, 0.17) | 0.953 | -40.82 (-52.55, -29.66) | <0.001 | — | — |
| Cholesteryl esters in medium HDL | -39.67 (-51.50, -23.42) | <0.001 | 0.02 (-0.13, 0.18) | 0.903 | -39.65 (-51.46, -23.37) | <0.001 | — | — |
| Free cholesterol in medium HDL | -40.14 (-52.41, -27.40) | <0.001 | 0.02 (-0.15, 0.17) | 0.896 | -40.12 (-52.26, -27.34) | <0.001 | — | — |
| Triglycerides in medium HDL | -40.44 (-56.53, -27.97) | <0.001 | -0.21 (-0.44, -0.06) | <0.001 | -40.64 (-56.81, -28.20) | <0.001 | 0.5 (0.2, 1.4) | <0.001 |
| Concentration of small HDL particles | -40.72 (-53.37, -28.72) | <0.001 | 0.04 (-0.09, 0.18) | 0.759 | -40.68 (-53.34, -28.63) | <0.001 | — | — |
| Cholesterol in small HDL | -39.74 (-51.50, -25.37) | <0.001 | 0.05 (-0.11, 0.21) | 0.756 | -39.69 (-51.36, -25.27) | <0.001 | — | — |
| Cholesteryl esters in small HDL | -40.20 (-54.92, -25.62) | <0.001 | 0.02 (-0.15, 0.20) | 0.903 | -40.18 (-54.81, -25.63) | <0.001 | — | — |
| Free cholesterol in small HDL | -40.71 (-52.92, -27.38) | <0.001 | 0.06 (-0.03, 0.23) | 0.456 | -40.65 (-52.75, -27.30) | <0.001 | — | — |
| Triglycerides in small HDL | -40.09 (-54.45, -24.69) | <0.001 | -0.33 (-0.55, -0.16) | <0.001 | -40.42 (-54.87, -25.00) | <0.001 | 0.8 (0.4, 1.8) | <0.001 |

CI, confidence interval; FDR, false discovery rate; HDL-C, high-density lipoprotein cholesterol; HDL, high-density lipoprotein; LDL, low-density lipoprotein; VLDL, very low-density lipoprotein; IDL, Intermediate Density Lipoprotein.

The models were adjusted for age, sex, ethnicity, educational attainment, occupational status, Townsend deprivation index, smoking status, alcohol consumption, healthy diet, regular exercise, sleep duration, body mass index, and waist circumference.

# **Supplementary Table S26. Mediation proportion of probable sarcopenia in interstitial lung disease attributed to metabolites (N=166,721).**

| **Metabolites** | **Direct effect (β)**  **(95% CI)** | **FDR** | **Indirect effect (β) (95% CI)** | **FDR** | **Total effect (β)**  **(95% CI)** | **FDR** | **Mediation proportion (%) (95% CI)** | **FDR** |
| --- | --- | --- | --- | --- | --- | --- | --- | --- |
| Total cholesterol | -60.70 (-89.81, -36.33) | <0.001 | -1.14 (-1.86, -0.55) | <0.001 | -61.84 (-91.08, -37.43) | <0.001 | 1.8 (0.8, 3.5) | <0.001 |
| Total cholesterol minus HDL-C | -60.02 (-88.70, -34.46) | <0.001 | -0.95 (-1.59, -0.40) | <0.001 | -60.97 (-90.13, -35.27) | <0.001 | 1.6 (0.7, 2.9) | <0.001 |
| Remnant cholesterol (non-HDL, non-LDL -cholesterol) | -60.23 (-89.12, -36.06) | <0.001 | -0.55 (-1.08, -0.10) | 0.026 | -60.78 (-89.77, -36.71) | <0.001 | 0.8 (0.2, 2.1) | 0.026 |
| Clinical LDL cholesterol | -61.48 (-98.84, -33.72) | <0.001 | -1.12 (-1.82, -0.48) | <0.001 | -62.61 (-100.19, -35.25) | <0.001 | 1.8 (0.7, 4.0) | <0.001 |
| LDL cholesterol | -59.21 (-87.61, -35.74) | <0.001 | -1.14 (-1.79, -0.64) | <0.001 | -60.35 (-88.89, -37.08) | <0.001 | 1.9 (1.0, 3.6) | <0.001 |
| HDL cholesterol | -64.10 (-95.23, -41.23) | <0.001 | -0.55 (-1.11, -0.08) | 0.026 | -64.65 (-95.74, -41.63) | <0.001 | 0.8 (0.2, 2.2) | 0.026 |
| Triglycerides in HDL | -61.98 (-90.72, -33.43) | <0.001 | 0.06 (-0.19, 0.35) | 0.789 | -61.92 (-90.63, -33.35) | <0.001 | — | — |
| Total phospholipids in lipoprotein particles | -62.95 (-81.66, -39.26) | <0.001 | -0.60 (-1.08, -0.20) | <0.001 | -63.55 (-82.34, -39.73) | <0.001 | 1.0 (0.3, 1.8) | <0.001 |
| Phospholipids in LDL | -58.61 (-82.98, -36.28) | <0.001 | -0.96 (-1.72, -0.37) | <0.001 | -59.57 (-83.64, -36.94) | <0.001 | 1.6 (0.7, 3.3) | <0.001 |
| Total esterified cholesterol | -59.17 (-81.14, -35.28) | <0.001 | -1.17 (-1.91, -0.67) | <0.001 | -60.34 (-82.72, -36.61) | <0.001 | 1.9 (1.0, 3.9) | <0.001 |
| Cholesteryl esters in VLDL | -64.67 (-93.45, -42.81) | <0.001 | -0.27 (-0.68, -0.01) | 0.048 | -64.94 (-93.70, -43.17) | <0.001 | 0.4 (0.0, 1.1) | 0.048 |
| Cholesteryl esters in LDL | -59.67 (-87.36, -35.21) | <0.001 | -1.06 (-1.71, -0.47) | <0.001 | -60.72 (-88.46, -35.96) | <0.001 | 1.7 (0.7, 3.3) | <0.001 |
| Cholesteryl esters in HDL | -65.79 (-98.75, -39.60) | <0.001 | -0.60 (-1.39, -0.17) | <0.001 | -66.39 (-99.46, -40.18) | <0.001 | 0.9 (0.3, 2.1) | <0.001 |
| Total free cholesterol | -61.78 (-90.13, -37.31) | <0.001 | -0.88 (-1.66, -0.37) | <0.001 | -62.66 (-91.04, -37.98) | <0.001 | 1.4 (0.6, 2.8) | <0.001 |
| Free cholesterol in LDL | -59.71 (-94.07, -35.84) | <0.001 | -1.29 (-1.98, -0.54) | <0.001 | -61.01 (-95.81, -38.02) | <0.001 | 2.1 (1.0, 3.9) | <0.001 |
| Free cholesterol in HDL | -61.95 (-91.07, -34.41) | <0.001 | -0.19 (-0.60, 0.12) | 0.29 | -62.14 (-90.99, -34.40) | <0.001 | — | — |
| Total lipids in lipoprotein particles | -58.60 (-90.79, -28.79) | <0.001 | -0.64 (-1.18, -0.24) | <0.001 | -59.24 (-91.46, -29.12) | <0.001 | 1.0 (0.4, 2.9) | <0.001 |
| Total lipids in LDL | -62.07 (-91.57, -36.13) | <0.001 | -1.04 (-1.74, -0.48) | <0.001 | -63.11 (-93.17, -37.11) | <0.001 | 1.6 (0.8, 3.6) | <0.001 |
| Total lipids in HDL | -61.38 (-87.30, -36.65) | <0.001 | -0.38 (-0.77, -0.07) | 0.048 | -61.76 (-87.75, -36.86) | <0.001 | 0.6 (0.1, 1.8) | 0.048 |
| Total concentration of lipoprotein particles | -63.04 (-89.04, -34.25) | <0.001 | -1.16 (-1.88, -0.48) | <0.001 | -64.20 (-90.24, -35.81) | <0.001 | 1.7 (0.7, 4.7) | <0.001 |
| Concentration of LDL particles | -61.01 (-93.39, -34.49) | <0.001 | -0.54 (-1.05, -0.18) | <0.001 | -61.55 (-93.83, -34.94) | <0.001 | 0.9 (0.3, 2.4) | <0.001 |
| Concentration of HDL particles | -62.68 (-93.43, -39.13) | <0.001 | -0.98 (-1.83, -0.49) | <0.001 | -63.65 (-94.33, -39.91) | <0.001 | 1.5 (0.7, 3.0) | <0.001 |
| Phosphoglycerides | -63.75 (-89.04, -39.84) | <0.001 | -0.46 (-0.92, -0.16) | <0.001 | -64.21 (-89.43, -40.44) | <0.001 | 0.7 (0.3, 1.6) | <0.001 |
| Total cholines | -60.70 (-85.11, -37.82) | <0.001 | -0.63 (-1.19, -0.26) | <0.001 | -61.34 (-86.04, -38.48) | <0.001 | 1.0 (0.5, 2.3) | <0.001 |
| Phosphatidylcholines | -62.65 (-90.81, -38.53) | <0.001 | -0.53 (-1.16, -0.23) | <0.001 | -63.18 (-91.41, -38.98) | <0.001 | 0.8 (0.3, 1.7) | <0.001 |
| Sphingomyelins | -61.33 (-95.35, -35.80) | <0.001 | -0.49 (-1.02, -0.13) | <0.001 | -61.82 (-95.69, -36.36) | <0.001 | 0.7 (0.2, 2.0) | <0.001 |
| Apolipoprotein B | -61.13 (-86.98, -36.02) | <0.001 | -0.56 (-1.11, -0.16) | <0.001 | -61.69 (-87.40, -36.73) | <0.001 | 0.9 (0.3, 2.3) | <0.001 |
| Apolipoprotein A1 | -64.14 (-90.60, -36.05) | <0.001 | -0.58 (-1.19, -0.16) | <0.001 | -64.72 (-91.24, -36.56) | <0.001 | 0.9 (0.2, 2.2) | <0.001 |
| Degree of unsaturation | -62.14 (-94.32, -36.71) | <0.001 | -1.67 (-2.52, -0.68) | <0.001 | -63.82 (-95.81, -38.80) | <0.001 | 2.7 (1.2, 5.3) | <0.001 |
| Omega-3 fatty acids | -61.56 (-91.02, -30.76) | <0.001 | -1.03 (-1.82, -0.45) | <0.001 | -62.59 (-92.41, -31.42) | <0.001 | 1.6 (0.7, 3.8) | <0.001 |
| Omega-6 fatty acids | -59.24 (-96.22, -26.69) | <0.001 | -0.63 (-1.14, -0.19) | <0.001 | -59.88 (-96.50, -27.17) | <0.001 | 1.0 (0.3, 2.5) | <0.001 |
| Polyunsaturated fatty acids | -60.06 (-89.22, -37.77) | <0.001 | -0.90 (-1.78, -0.30) | <0.001 | -60.96 (-90.55, -38.47) | <0.001 | 1.5 (0.5, 3.0) | <0.001 |
| Linoleic acid | -58.30 (-82.05, -32.03) | <0.001 | -0.65 (-1.15, -0.22) | <0.001 | -58.95 (-82.72, -32.75) | <0.001 | 1.1 (0.4, 2.6) | <0.001 |
| Docosahexaenoic acid | -60.88 (-92.11, -35.96) | <0.001 | -1.52 (-2.45, -0.85) | <0.001 | -62.40 (-93.79, -37.12) | <0.001 | 2.4 (1.5, 4.1) | <0.001 |
| Alanine | -64.24 (-95.45, -37.13) | <0.001 | -0.01 (-0.27, 0.26) | <0.001 | -64.25 (-95.52, -37.05) | <0.001 | — | — |
| Glutamine | -64.01 (-95.19, -37.99) | <0.001 | -0.38 (-0.88, 0.03) | 0.091 | -64.39 (-95.76, -37.95) | <0.001 | — | — |
| Histidine | -63.74 (-85.94, -38.96) | <0.001 | -0.50 (-1.03, -0.12) | <0.001 | -64.25 (-86.57, -39.52) | <0.001 | 0.8 (0.2, 2.0) | <0.001 |
| Phenylalanine | -64.67 (-91.00, -37.29) | <0.001 | -0.88 (-1.49, -0.47) | <0.001 | -65.55 (-91.82, -38.04) | <0.001 | 1.3 (0.7, 2.6) | <0.001 |
| Citrate | -61.44 (-89.44, -36.42) | <0.001 | 0.44 (-0.48, 1.37) | <0.001 | -61.00 (-88.26, -36.06) | <0.001 | — | — |
| Acetoacetate | -63.59 (-89.38, -41.67) | <0.001 | -0.05 (-0.24, 0.12) | 0.560 | -63.64 (-89.33, -41.62) | <0.001 | — | — |
| Albumin | -59.59 (-87.66, -34.87) | <0.001 | -4.87 (-6.62, -3.32) | <0.001 | -64.46 (-92.43, -38.77) | <0.001 | 8.0 (5.0, 12.4) | <0.001 |
| Glycoprotein acetyls | -61.93 (-86.27, -32.18) | <0.001 | -0.63 (-1.42, 0.15) | 0.091 | -62.56 (-87.00, -33.25) | <0.001 | — | — |
| Concentration of medium VLDL particles | -63.61 (-89.01, -41.78) | <0.001 | -0.32 (-0.83, -0.05) | 0.048 | -63.93 (-89.47, -42.03) | <0.001 | 0.5 (0.1, 1.0) | 0.048 |
| Phospholipids in medium VLDL | -62.83 (-86.51, -35.24) | <0.001 | -0.37 (-0.92, 0.01) | 0.091 | -63.20 (-86.91, -35.29) | <0.001 | 0.5 (0.0, 1.8) | 0.091 |
| Cholesterol in medium VLDL | -65.90 (-90.18, -39.01) | <0.001 | -0.66 (-1.21, -0.24) | <0.001 | -66.56 (-90.78, -40.09) | <0.001 | 1.0 (0.4, 2.1) | <0.001 |
| Cholesteryl esters in medium VLDL | -61.15 (-85.24, -36.30) | <0.001 | -0.73 (-1.37, -0.19) | <0.001 | -61.88 (-86.21, -36.98) | <0.001 | 1.2 (0.3, 2.4) | <0.001 |
| Free cholesterol in medium VLDL | -60.57 (-85.22, -32.21) | <0.001 | -0.47 (-0.82, -0.17) | <0.001 | -61.04 (-85.69, -32.77) | <0.001 | 0.8 (0.3, 1.7) | <0.001 |
| Free cholesterol in small VLDL | -62.75 (-91.37, -39.63) | <0.001 | -0.38 (-0.78, -0.06) | 0.026 | -63.13 (-91.95, -40.01) | <0.001 | 0.6 (0.1, 1.5) | 0.026 |
| Cholesterol in very small VLDL | -60.77 (-84.95, -33.98) | <0.001 | -0.09 (-0.41, 0.21) | 0.593 | -60.86 (-85.08, -33.97) | <0.001 | — | — |
| Cholesteryl esters in very small VLDL | -61.69 (-87.91, -35.18) | <0.001 | -0.15 (-0.56, 0.27) | 0.440 | -61.84 (-88.19, -35.52) | <0.001 | — | — |
| Concentration of IDL particles | -62.15 (-88.08, -35.64) | <0.001 | -0.42 (-0.96, -0.01) | 0.071 | -62.58 (-89.03, -36.07) | <0.001 | — | — |
| Total lipids in IDL | -59.88 (-90.31, -34.61) | <0.001 | -0.66 (-1.36, -0.12) | <0.001 | -60.54 (-90.93, -34.98) | <0.001 | 1.0 (0.2, 2.6) | <0.001 |
| Phospholipids in IDL | -62.54 (-92.99, -35.15) | <0.001 | -0.46 (-1.03, -0.01) | 0.048 | -62.99 (-93.21, -36.01) | <0.001 | — | — |
| Cholesterol in IDL | -63.49 (-87.02, -40.44) | <0.001 | -0.80 (-1.58, -0.21) | 0.026 | -64.29 (-87.98, -41.24) | <0.001 | 1.2 (0.3, 2.7) | 0.026 |
| Cholesteryl esters in IDL | -62.06 (-88.28, -39.74) | <0.001 | -0.89 (-1.74, -0.25) | 0.026 | -62.96 (-89.35, -40.38) | <0.001 | 1.4 (0.4, 2.7) | 0.026 |
| Free cholesterol in IDL | -64.05 (-95.50, -36.56) | <0.001 | -0.63 (-1.39, 0.05) | 0.071 | -64.68 (-96.54, -36.95) | <0.001 | 1.0 (-0.1, 2.4) | 0.071 |
| Triglycerides in IDL | -66.15 (-96.13, -33.84) | <0.001 | -0.21 (-0.68, 0.14) | 0.301 | -66.36 (-96.16, -33.97) | <0.001 | — | — |
| Concentration of large LDL particles | -63.85 (-88.29, -41.37) | <0.001 | -0.51 (-1.01, -0.16) | <0.001 | -64.36 (-88.47, -41.82) | <0.001 | 0.8 (0.3, 1.8) | <0.001 |
| Total lipids in large LDL | -62.12 (-86.97, -40.08) | <0.001 | -1.06 (-1.72, -0.48) | <0.001 | -63.18 (-87.97, -40.88) | <0.001 | 1.7 (0.8, 3.2) | <0.001 |
| Phospholipids in large LDL | -62.50 (-90.85, -36.01) | <0.001 | -1.06 (-1.88, -0.50) | <0.001 | -63.56 (-92.40, -36.85) | <0.001 | 1.6 (0.8, 3.3) | <0.001 |
| Cholesterol in large LDL | -59.27 (-86.41, -39.20) | <0.001 | -1.24 (-2.19, -0.63) | <0.001 | -60.51 (-87.59, -40.24) | <0.001 | 2.0 (1.0, 3.9) | <0.001 |
| Cholesteryl esters in large LDL | -59.87 (-84.81, -32.89) | <0.001 | -1.20 (-1.89, -0.58) | <0.001 | -61.07 (-86.21, -33.91) | <0.001 | 1.9 (0.9, 4.0) | <0.001 |
| Free cholesterol in large LDL | -63.22 (-90.33, -37.97) | <0.001 | -1.30 (-2.30, -0.62) | <0.001 | -64.53 (-92.04, -39.30) | <0.001 | 2.0 (0.8, 3.6) | <0.001 |
| Concentration of medium LDL particles | -62.26 (-93.54, -34.44) | <0.001 | -0.57 (-1.13, -0.13) | <0.001 | -62.83 (-94.18, -34.77) | <0.001 | 0.9 (0.3, 1.8) | <0.001 |
| Total lipids in medium LDL | -61.59 (-84.67, -36.42) | <0.001 | -0.82 (-1.39, -0.31) | <0.001 | -62.41 (-85.28, -37.38) | <0.001 | 1.3 (0.5, 3.0) | <0.001 |
| Phospholipids in medium LDL | -64.06 (-88.15, -43.80) | <0.001 | -0.84 (-1.47, -0.35) | <0.001 | -64.90 (-88.67, -44.59) | <0.001 | 1.3 (0.6, 2.4) | <0.001 |
| Cholesterol in medium LDL | -60.73 (-92.27, -37.44) | <0.001 | -0.98 (-1.73, -0.41) | <0.001 | -61.71 (-93.30, -38.46) | <0.001 | 1.6 (0.7, 2.9) | <0.001 |
| Cholesteryl esters in medium LDL | -59.78 (-85.04, -33.07) | <0.001 | -0.77 (-1.22, -0.29) | <0.001 | -60.56 (-85.66, -33.72) | <0.001 | 1.3 (0.5, 2.9) | <0.001 |
| Free cholesterol in medium LDL | -60.12 (-88.52, -36.02) | <0.001 | -1.28 (-2.13, -0.58) | <0.001 | -61.40 (-90.22, -37.77) | <0.001 | 2.1 (0.9, 3.7) | <0.001 |
| Concentration of small LDL particles | -59.52 (-87.85, -32.90) | <0.001 | -0.38 (-0.84, -0.10) | 0.026 | -59.91 (-87.96, -33.18) | <0.001 | 0.6 (0.2, 1.6) | 0.026 |
| Total lipids in small LDL | -60.48 (-84.31, -33.70) | <0.001 | -0.58 (-1.10, -0.16) | <0.001 | -61.06 (-85.09, -34.13) | <0.001 | 0.9 (0.3, 2.1) | <0.001 |
| Phospholipids in small LDL | -60.48 (-89.00, -33.15) | <0.001 | -0.56 (-1.08, -0.2) | <0.001 | -61.04 (-89.93, -33.47) | <0.001 | 0.9 (0.3, 2.5) | <0.001 |
| Cholesterol in small LDL | -60.17 (-86.04, -40.82) | <0.001 | -0.78 (-1.54, -0.32) | <0.001 | -60.95 (-86.73, -41.67) | <0.001 | 1.2 (0.6, 2.4) | <0.001 |
| Cholesteryl esters in small LDL | -61.07 (-97.94, -35.78) | <0.001 | -0.62 (-1.13, -0.18) | <0.001 | -61.70 (-98.53, -36.19) | <0.001 | 1.0 (0.3, 2.3) | <0.001 |
| Free cholesterol in small LDL | -63.46 (-90.12, -34.36) | <0.001 | -1.04 (-1.81, -0.37) | <0.001 | -64.51 (-91.40, -35.57) | <0.001 | 1.6 (0.6, 3.5) | <0.001 |
| Concentration of medium HDL particles | -63.48 (-89.06, -40.71) | <0.001 | -0.4 (-0.92, -0.09) | 0.026 | -63.88 (-89.49, -40.97) | <0.001 | 0.6 (0.1, 1.4) | 0.026 |
| Total lipids in medium HDL | -62.05 (-93.33, -37.55) | <0.001 | -0.32 (-0.63, -0.06) | <0.001 | -62.36 (-93.67, -37.83) | <0.001 | 0.5 (0.1, 1.2) | <0.001 |
| Cholesterol in medium HDL | -63.95 (-84.16, -40.77) | <0.001 | -0.55 (-1.15, -0.17) | <0.001 | -64.49 (-84.57, -41.34) | <0.001 | 0.8 (0.3, 1.9) | <0.001 |
| Cholesteryl esters in medium HDL | -62.98 (-90.54, -32.01) | <0.001 | -0.53 (-1.04, -0.13) | <0.001 | -63.51 (-91.20, -32.39) | <0.001 | 0.8 (0.2, 1.9) | <0.001 |
| Free cholesterol in medium HDL | -63.30 (-91.56, -40.63) | <0.001 | -0.43 (-0.90, -0.07) | <0.001 | -63.73 (-92.04, -41.03) | <0.001 | 0.7 (0.1, 1.5) | <0.001 |
| Triglycerides in medium HDL | -63.56 (-90.64, -40.45) | <0.001 | 0.11 (-0.26, 0.53) | 0.425 | -63.45 (-90.71, -40.54) | <0.001 | — | — |
| Concentration of small HDL particles | -63.38 (-94.43, -40.02) | <0.001 | -1.02 (-1.90, -0.41) | <0.001 | -64.41 (-95.34, -40.92) | <0.001 | 1.6 (0.5, 2.8) | <0.001 |
| Cholesterol in small HDL | -60.96 (-85.20, -34.98) | <0.001 | -1.07 (-1.85, -0.44) | <0.001 | -62.03 (-86.42, -35.58) | <0.001 | 1.7 (0.7, 3.3) | <0.001 |
| Cholesteryl esters in small HDL | -62.75 (-91.71, -36.54) | <0.001 | -1.26 (-2.12, -0.70) | <0.001 | -64.00 (-92.90, -37.85) | <0.001 | 1.9 (1.0, 3.6) | <0.001 |
| Free cholesterol in small HDL | -63.69 (-87.23, -37.42) | <0.001 | -0.53 (-1.24, -0.03) | 0.048 | -64.22 (-87.66, -38.00) | <0.001 | 0.7 (0.0, 2.3) | 0.048 |
| Triglycerides in small HDL | -64.18 (-92.81, -35.35) | <0.001 | 0.22 (-0.17, 0.69) | 0.301 | -63.96 (-92.23, -34.94) | <0.001 | — | — |

CI, confidence interval; FDR, false discovery rate; HDL-C, high-density lipoprotein cholesterol; HDL, high-density lipoprotein; LDL, low-density lipoprotein; VLDL, very low-density lipoprotein; IDL, Intermediate Density Lipoprotein.

The models were adjusted for age, sex, ethnicity, educational attainment, occupational status, Townsend deprivation index, smoking status, alcohol consumption, healthy diet, regular exercise, sleep duration, body mass index, and waist circumference.
